# Evolution of Transcriptomes in Early-Generation Hybrids of the Apomictic *Ranunculus auricomus* Complex (*Ranunculaceae*)

**DOI:** 10.3390/ijms232213881

**Published:** 2022-11-10

**Authors:** Claudia Paetzold, Birthe H. Barke, Elvira Hörandl

**Affiliations:** 1 Department of Botany and Molecular Evolution, Senckenberg Research Institute, 60325 Frankfurt am Main, Germany; 2 Department of Systematics, Biodiversity and Evolution of Plants (with Herbarium), University of Goettingen, 37073 Goettingen, Germany

**Keywords:** apospory, meiosis, sporogenesis, dN/dS ratios, angiosperms, hybrids

## Abstract

Hybridisation in plants may cause a shift from sexual to asexual seed formation (apomixis). Indeed, natural apomictic plants are usually hybrids, but it is still unclear how hybridisation could trigger the shift to apomixis. The genome evolution of older apomictic lineages is influenced by diverse processes such as polyploidy, mutation accumulation, and allelic sequence divergence. To disentangle the effects of hybridisation from these other factors, we analysed the transcriptomes of flowering buds from artificially produced, diploid F2 hybrids of the *Ranunculus auricomus* complex. The hybrids exhibited unreduced embryo sac formation (apospory) as one important component of apomixis, whereas their parental species were sexual. We revealed 2915 annotated single-copy genes that were mostly under purifying selection according to dN/dS ratios. However, pairwise comparisons revealed, after rigorous filtering, 79 genes under diversifying selection between hybrids and parents, whereby gene annotation assigned ten of them to reproductive processes. Four genes belong to the meiosis-sporogenesis phase (ASY1, APC1, MSP1, and XRI1) and represent, according to literature records, candidate genes for apospory. We conclude that hybridisation could combine novel (or existing) mutations in key developmental genes in certain hybrid lineages, and establish (together with altered gene expression profiles, as observed in other studies) a heritable regulatory mechanism for aposporous development.

## 1. Introduction

Natural hybridisation in flowering plants is a frequent phenomenon and may represent an important evolutionary force [1,2,3]. By merging previously diverged genomes, hybridisation creates genomic and phenotypic novelty already in the first hybrid generation. Plant hybrids do not necessarily exhibit sterility, but rather can express various degrees of fertility and may produce offspring. Whenever hybrids manage to produce at least some offspring, segregation in the F2 and following populations will create a high diversity of genotypes and phenotypes, which selection can act upon both intrinsically and extrinsically, sometimes even resulting in speciation [3,4]. Accordingly, selection pressure should be higher in newly formed hybrids than in parents. However, few studies exist on selection pressures in early generation hybrids in natural systems. Studies in sunflowers suggested that both intrinsic selection on fertility and extrinsic selection on phenotypic traits played a role in the establishment of hybrid lineages [5].

Hybridisation in plants is often connected to a shift in the mode of reproduction. Apomixis, i.e., asexual reproduction via asexually formed seeds [6,7], mostly appears in lineages that originated from hybrids of sexual progenitor species [7,8]. Apomixis appears in two main forms, gametophytic and sporophytic. The former is the relevant one for our study and involves two specific consecutive steps in female development: first, a bypass or alteration of meiosis, resulting in the formation of an unreduced gametophyte (here, out of a somatic cell, a pathway called apospory), and, second, the development of the unreduced egg cell without fertilisation (parthenogenesis). However, pollination and fertilisation of the nucleus of the central cell is often required for proper development of the endosperm, the nutritious tissue for the embryo (pseudogamy). Hence, pollen remains at least partly functional in most apomictic plants and undergoes meiotic development [9]. Apomixis is inheritable and under the control of separate Mendelian genetic factors for apospory and parthenogenesis in all the genera studied so far, and these processes can be uncoupled [10]. These Mendelian factors represent, usually, large, non-recombinant regions in the genome that are inherited as a linkage block [10]. The regulatory mechanisms of apomixis are complex and probably represent a deregulation of the sexual pathway [11,12]. Complex pathways for cell cycle control, hormonal pathways, RNA helicases, signal transduction, and even metabolic stage play a role in the expression of apomixis [12,13]. Epigenetic regulation of gene expression likely plays a major role [11,12]. Many efforts are targeted towards introducing apomixis into crops via genetic engineering [14].

The molecular background for the origins of apomixis in natural hybrids, however, remains unclear. One hypothesis suggests that hybridisation of different ecotypes with different developmental timing would result in asynchronous gene expression, and hence suppression of the sexual pathway [7]. In diploid apomictic hybrids of *Boechera*, expressed genes showed indeed signals of asynchrony, but also parent-of-origin effects and developmental stage-specific expression patterns [15,16]. However, chromosomal rearrangements could also play a role in apomixis [17,18,19,20]. Most natural apomictic plants are polyploids [8], which adds another level of complexity to regulatory mechanisms as genome duplication has many effects on genome structure and gene expression [21]. Recent reviews suggest that apomixis starts in diploid populations at low frequencies, and polyploidy probably has only an enhancing effect for the establishment of apomixis in populations [8,22,23]. However, in many polyploid model systems, the difficulty of disentangling the genomic features of hybridity, polyploidy, and stage-specific gene expression makes it difficult to pinpoint regulatory elements’ expression of apomixis [24]. 

The role of transcriptome evolution in hybrids for the expression of the apomictic developmental pathway is so far unclear. The classical idea that apomixis would be selected as an escape from meiotic disturbances and hybrid sterility [25] has received only equivocal support from more recent studies [12,26]. Hybridisation could have a “creative” role as sexual hybrids may produce novel genotypes and phenotypes via transgressive segregation in the F2 generation [27]. If such an early-generation hybrid expresses features of apomixis inherited from the F1 generation that would bypass the detrimental effects of hybridity on meiosis and fertilisation, then genes related to this trait would be expected to be under positive selection. Under this assumption, regulatory mechanisms for apospory and parthenogenesis would be positively selected. In the sexual parents, these genes would be expected to be either under purifying selection against negative mutations, or selectively neutral [28]. Selection studies rely on the commonly used ratios of non-synonymous versus synonymous substitutions in coding gene regions (dN/dS ratios). However, in natural, long-term established, and diverging asexual lineages, it is difficult to disentangle the long-term accumulation of deleterious mutations due to a lack of recombination and the reduced efficacy of purifying selection (Muller’s ratchet) [29] from positive selection on adaptive genes [30], as both processes will result in dN/dS ratios (ω) over one. Mutation accumulation has been observed in some established asexual plant lineages [31,32].

We use here the taxa of the Eurasian *Ranunculus auricomus* complex for a study of transcriptome evolution in early-generation hybrids. The complex comprises five sexual progenitor species distributed in Central and Southern Europe that diverged c. 0.7 Mill. years ago, and several polyploid apomictic lineages [33,34]. Phylogenomic and taxonomic analyses revealed the diploids *R. cassubicifolius* (including its close relative *R. carpaticola*, now regarded as conspecific) and *R. notabilis* as the genetically and morphologically most divergent lineages [35,36] and both occur allopatrically [36]. These species are obligate sexual outcrossers [37,38,39,40]. Naturally occurring polyploids of the complex are mostly apomictic lineages, with embryo sac formation starting from a somatic, unreduced cell of the nucellus (apospory) and parthenogenetic egg cell development [33,37,38]. Endosperm is usually formed after fertilisation of the central cell nucleus with one or both sperm nuclei (pseudogamy) [33,37,38]. Apospory is in the complex heritable and under the genetic control of a Mendelian “apospory factor” [37], but facultative, which means that sexual and aposporous ovules can be formed within the same flower (c. 20–40 ovules per flower). The apospory factor is allelic with dominant expression, similar as in other plants, and likely covers a large, non-recombinant genetic region [10]. However, this region and the underlying genes are not yet characterised in *Ranunculus*. Natural apomictic hexaploid hybrids and sexual species of the *R. auricomus* complex differ in gene expression profiles within the ovules in hundreds of genes, whereby genes related to the meiosis and gametogenesis stage were mostly influenced by transgressive effects, but also by ploidy and parent-of-origin effects [24]. SNP analysis of the transcriptomes of these hexaploid apomictic lineages (thereafter called established natural apomicts throughout the manuscript) suggested an age of c. 70,000 years, but revealed no genome-wide mutation accumulation in dN/dS ratios [30], probably due to facultative sexuality and sufficient purifying selection [41]. Nevertheless, some genes with high dN/dS values (outliers > 1) diversified between sexual and apomictic lineages, and included nine genes related to reproductive development in the meiosis and gametogenesis stage [30]. However, in that study, it was not possible to discriminate negative, accumulated mutations from putative gain-of-function mutations related to apospory. 

Previous crossing experiments of the *R. cassubicifolius/carpaticola* lineage with *R. notabilis* revealed a diploid F1 generation with spontaneous appearance of apospory in low frequencies (means of c. 11%) [38]. Parthenogenesis, however, was not yet observed in these diploid plants. Further intercrossing of these F1 plants revealed a sexually formed, diploid F2 that exhibited increasing proportions of aposporous embryo sac formation up to 30% [42]. These experiments confirmed the heritability of genetic factors of apospory via haploid gametes, and showed allelic dosage effects of control factors on the expression of apospory [37]. We use here individuals of this F2 generation and the parental sexual species to analyse the initial evolution of transcriptomes in hybrids (Supplementary Table S1). 

By using experimentally produced, diploid F2 hybrids from controlled crosses, we can rule out many side effects that would appear in natural apomictic lineages: first, we can directly compare the transcriptomes of known parental species/hybrids; second, we can exclude the effects of polyploidy, as well as the subgenome dominance effects of one parental genome over time [34]; third, we would, in a second-generation hybrid, neither expect an effect of Muller’s ratchet nor of random drift on transcriptomes. By sequencing transcriptomes of flowering buds in all stages before anthesis, and a rigorous filtering approach to overcome the biases of dN/dS ratios [43], we focus here on genes diversifying between sexual parents and aposporous F2 hybrids. We want to address the following questions: (1) Do we find genes in diploid aposporous F2 hybrids that diverge from their sexual parents? (2) Are these genes related to reproductive development and apospory? (3) How do our findings compare to genes with elevated dN/dS ratios in established natural apomictic lineages of the complex [30]? Based on these results, we will discuss hypothetical scenarios for the evolution of apomixis in natural hybrids.

## 2. Results

### 2.1. Transcriptome Data

Illumina sequencing produced an average of 3.78 (2.75–5.61) million 250 bp paired-end reads. Trimming removed an average of 0.89% (0.21–1.37) of reads. The number of assembled contigs per sample ranged between 33,233 (C4) and 25,501 (P15(02)), with an average of 29,061. Contig lengths are comparable between samples (Table 1).

**Figure 1:**
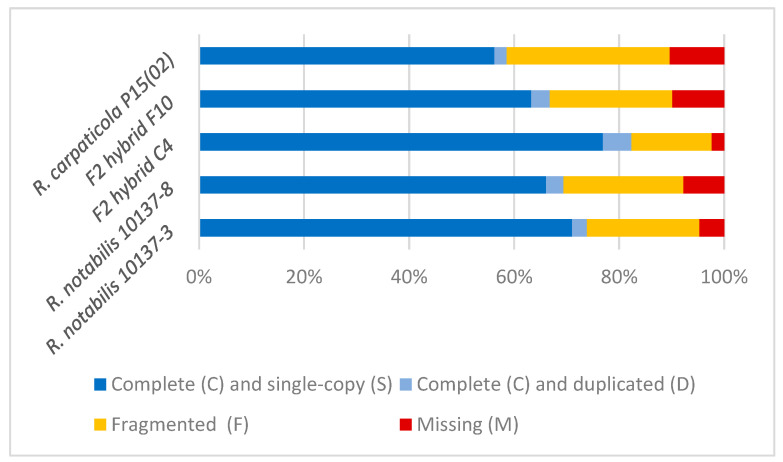
Summary of Benchmarking Universal Single-Copy Orthologues (BUSCO) analysis results for the assembled transcriptomes of the five studied individuals against the Viridiplantae BUSCO dataset. Total number of analysed BUSCOs: 425. Complete BUSCOS (C) [=Complete and single-copy BUSCOs (S) + Complete and duplicated BUSCOs (D)], Fragmented BUSCOs (F), Missing BUSCOs (M).

For all of the investigated samples, the majority of the 425 BUSCOS included in the Viridiplantae dataset were found to be present and single-copy (Figure 1). The F2 hybrid C4 shows the highest number of recovered BUSCOs and the lowest number of missing ones, while *R. carpaticola* P15(02) shows the lowest number of recovered BUSCOs and the highest number of missing ones. However, overall the results of BUSCO are very comparable amongst the five samples and illustrate that a substantial number of known single-copy benchmark genes could be recovered in the samples. 

ProteinOrtho identified 93,082 coding sequences, of which 4170 are putatively single-copy in the five samples. From these, 1254 could either not be aligned over all samples, or could not be functionally annotated. A further 408 were identified as putative non-spermatophyte genes and removed. Of the remaining 2507 contigs (Supplementary Table S2), trimming to remove alignments with less than 30 contiguous amino acids overlapping across all samples resulted in 1514 contigs, which were subjected to dN/dS ratio estimation.

The majority of analysed contigs showed sequence variability both between and within the sample groups, i.e., the hybrids or the parental taxa (Supplementary Figure S1). A relatively small number of genes are 100% identical within the three parental taxa or the two F2 hybrid samples, respectively. Sequence variability within the parental samples is, in equal measure, a consequence of variability between the two species, *R. carpaticola* and *R. notabilis*, as well as due to variability within the two *R. notabilis* individuals (Supplementary Figure S1). Reconstruction of minimum hybridisation networks from the ML gene trees of the 1514 aligned contigs resulted in a set of six minimum networks (Supplementary Figure S2). All networks infer two separate hybridisation events. However, either of the hybrids is recognised as the product of a hybridisation event in three networks, and another two each as a parental taxon.

The percentage of heterozygous sites across loci varies by a factor of up to ten (Supplementary Table S3). The two parental taxa, *R. notabilis* (10137-8) and *R. carpaticola* (P12(02)), possess the highest (2.66%) and lowest average percentage (1.87%) of heterozygous sites across loci. However, the F2 hybrid specimens show similar average percentages, with C4 showing a higher average percentage (2.58%) than F10 (1.96%).

### 2.2. dN/dS Ratios and Genes under Diversifying Selection in Parent–Hybrid Comparisons

**Figure 2:**
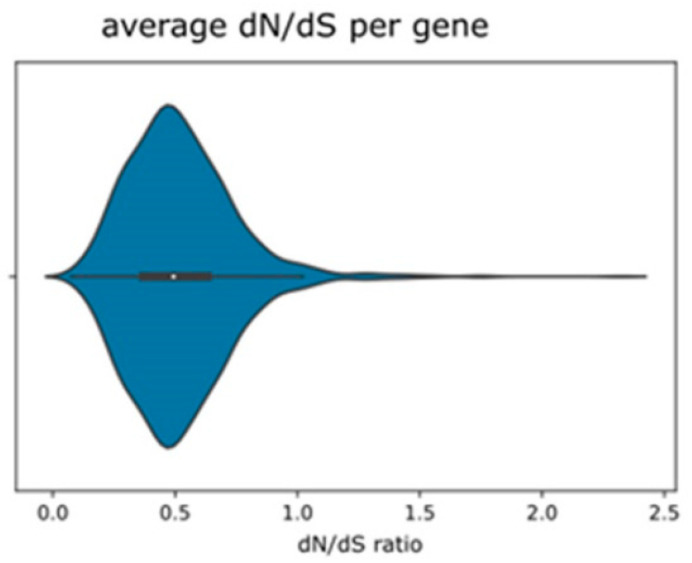
Violin plot depicting average dN/dS ratios across putative single-copy loci over all samples as inferred by Nej–Gojobori algorithm using Bioperl.

Average dN/dS ratios are generally low (Figure 2), with the majority of loci presenting ratios < 1. However, a small number of loci are inferred to be under average diversifying selection (Figure 2, Supplementary Table S4). Pairwise comparison of Nej–Gojobori dN/dS ratios (Supplementary Figure S3 and Table S5) reveals that the majority of loci in each pair are subject to no or to purifying selection; some loci show a strong signal for diversifying selection between the parental and the F2 taxon. In addition, boxplots illustrating all ten pairwise dN/dS ratios, especially compared between C4 and the *R. notabilis* individuals, show higher upper quartiles compared to purely parental or hybrid pairs (Supplementary Figure S3). Filtering out the genes affected by high parental divergence and segregation in the F2, intraspecific polymorphisms, and high divergence between the two hybrid samples, eliminated altogether 240 loci (see Table 2 and Section 4). From the remaining loci, the great majority (1171) had, in the parent–hybrid comparison, dN/dS values equal to or below 1.0, i.e., were under purifying selection. From the 98 with dN/dS >1.0, we revealed a subset of 79 genes that could be annotated and can be regarded to carry true substitutions under diversifying selection between hybrids and parents (average ω > 1.0; Table 2; Supplementary Table S5.

### 2.3. Gene Annotation Related to Reproduction

**Figure 3:**
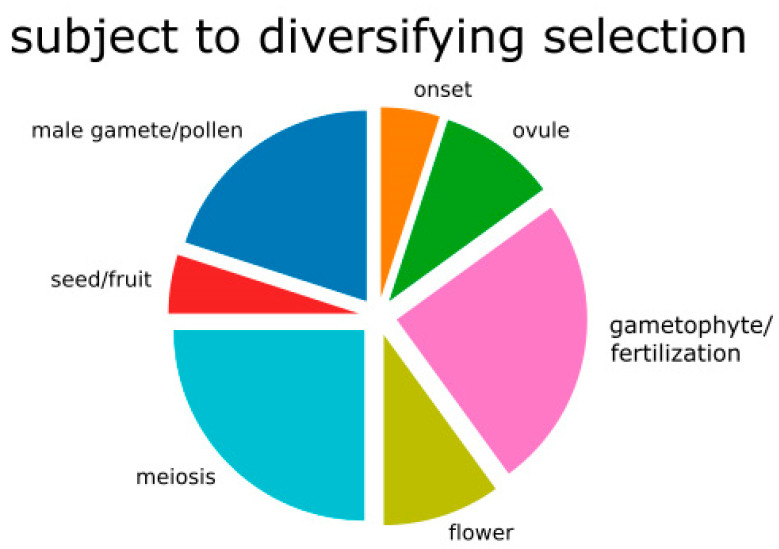
Pie chart of genes involved in reproduction and inferred to be under diversifying selection in the F2 hybrids compared to the parental samples. “Onset” means genes involved in onset of reproductive process.

Amongst the 2915 aligned contigs, 368 are functionally linked to reproduction (Supplementary Figure S4 and Table S6), representing genes active in all aspects of the reproductive process (Supplementary Table S6 and Figure S5). Among the 79 genes that were filtered for diversifying selection in the F2 hybrid–parent comparisons, a total of 10 (20 functional GO terms) are related to reproduction (Table 3), encompassing all stages of the reproductive process. Sorting of GO terms according to the five main reproductive stages (Table 3) revealed that two (10%) belong to flower and ovule formation, five (25%) to meiosis, five (25%) to gametophyte development (one of them to embryo sac development), one (5%) to the mature embryo sac and seed development stage, two (10%) to ovule development, and four (20%) to male functions (Figure 3).

## 3. Discussion

Here, we analyse, for the first time, the transcriptomes of a synthetic aposporous F2 hybrid generation compared to their parents, with a focus on changes in genes related to the mode of reproduction. Previous studies on the genome evolution of asexual plants used established, natural asexual hybrid lineages, in which the side effects of polyploidy and long-term processes such as allelic sequence divergence, mutation accumulation, and different ecological adaptations influence genome evolution [30,31,32]. By analysing transcriptomes over all reproductive developmental stages in flower buds, we intended to obtain insights into genetic changes in the evolutionary transition phase from sexual to asexual reproduction.

### 3.1. Factors Influencing Selection Regimes and dN/dS Values

In angiosperms, genome-wide selection regimes are influenced by recombination rates, genome size, and architecture, but also the effective population size [28]. Higher recombination rates increase, in general, the efficacy of purifying selection, while genome size is negatively correlated to recombination rates. The diploid sexual species in *R. auricomus* have a quite large genome (c. 610 MB; [44]), implying that recombination rates would be expected at the lower end of angiosperms [28]. Since a complete reference genome for *Ranunculus* is missing so far, further conclusions cannot be drawn in this respect. However, our filtering for orthologues (see Section 4) resulted in a similar number of recovered orthologues compared to other, broadly sampled angiosperms [45]. Analyses of dN/dS ratios are a classical method to study genome evolution. These ratios are potentially biased by small sample sizes, underestimating the amount of unfixed polymorphisms in parental populations, and by low numbers of generations, resulting in overestimates of dN/dS values [43]. In sexual hybrids, some false positive substitution calls could be due to fixed divergent polymorphisms of the parents that are combined in the F1 and segregate in the F2 [46]. Mendelian segregation of our F2 hybrids was proven earlier by morphological and molecular markers [47]. Comparison of sequence variability within the F2 hybrids and the parental samples, respectively, illustrated that this might be the case here (Supplementary Figure S1). Inference of hybridisation networks resulted in six minimal hybridization networks [48] with different network topologies. However, algorithms aiming at the reconstruction of hybrid–parent combinations from molecular or gene tree data currently share this phenomenon due to the complexity of the problem [49,50]. Moreover, Mendelian segregation in the F2 influences the relationships of samples. Nevertheless, the inferred hybridisation networks generally infer a reticulate relationship between the F2 hybrid samples and the three samples originating from the same wild population as the expired parental individuals. 

We reduced polymorphisms by the rigorous filtering and removal of divergent interspecific loci, which represented a considerable fraction (9%) of all loci (Table 2). We further reduced parental polymorphisms by removing divergent loci between the *R. notabilis* samples, but these represented only c. 5% of all loci (Table 2, Supplementary Figure S1). Although some polymorphisms may have remained in the final dataset, the proportions of these are probably very low. The hybrid–hybrid comparison reduced strongly divergent loci between the two hybrids, as these are not of interest for a shared trait (apospory). By choosing the conservative counting Nej–Gojobori algorithm, we avoided potential overestimates of substitution rates, as reported for model-based algorithms [51]. Results can, to some extent, be further compared to the transcriptome evolution of established natural apomictic *R. auricomus* lineages ([30]). With this conservative approach, we may have missed single sites with true substitutions between hybrids and parents. Nevertheless, we could narrow down a subset of ten genes with signals of diversifying selection between parents and F2 hybrids, and an apparent relationship with reproductive development. This descriptive approach could be a fast and cheap approach to identify candidate genes for further functional studies in natural apomictic model systems, for which mutant lines and reference genomes are not available.

### 3.2. Transcriptome Evolution between Hybrids and Parents Related to Apospory 

Our study revealed that purifying selection is the strongest force in sexual parents, both compared to other selection regimes within species and compared to the hybrids. Purifying selection against deleterious mutations is expected to be predominant in sexual species, in which recombination can efficiently purge the genome of harmful mutations [28]. In the F2 hybrids, purifying selection is also active, as this generation was still produced by sexual fertilisation (i.e., without parthenogenesis) [42], and hence showed Mendelian segregation patterns [47]. These results are also in accordance with previous transcriptome studies that revealed predominantly purifying selection for the same parental species and established natural apomicts [30]. However, in contrast to the established, natural apomictic lineages, we did not find elevated levels of heterozygosity in the F2 hybrids compared to the parents. The established natural apomicts exhibited allelic sequence divergence (Meselson effects [52]) in the polyploid genomes [30]. Percentages of heterozygous sites of sexual species were in the range of previous estimates for *R. auricomus* [33]. Considering the ten genes under diversifying selection in the parent–hybrid comparison linked to the reproductive process, half of them belong to meiosis–gametophyte stages, which are potentially important for the discrimination of sexual or aposporous development. Contig_4136 represents the meiosis-specific protein ASYNAPTIC 1 (ASY1), which is involved in chiasma assembly and homologous chromosome pairing at meiosis, and it is also required for DMC1-mediated interhomologue recombination [53]. Strikingly, the respective GO term was also found among the genes with outlier dN/dS ratios in the established natural apomictic lineages of *Ranunculus auricomus* [30] (Table 3). In a gene expression study on diploid *Boechera*, ASY1 was found to be downregulated in apomictic plants, probably due to global DNA methylation changes exclusively in the apomicts [53]. A second diversified meiosis gene found here was X-RAY-INDUCED 1. XRI1 is essential for male and female meiosis, and xri1 mutants display disrupted meiosis with strong chromosome fragmentation [54]. Thirdly, APC1 is part of the anaphase-promoting complex, which is involved in mitosis and plays a role during gametogenesis and embryogenesis; *Arabidopsis* mutants for this gene exhibited aborted ovules and seeds [55], a condition that was also observed in our F2 hybrids [42]. A remarkably diversified gene of the sporogenesis stage was the leucine-rich repeat receptor protein kinase MULTIPLE SPOROCYTES (MSP1), which plays important roles in restricting the number of cells entering into male and female sporogenesis [56]. In rice and maize, msp1 mutants show relaxed control over sporocyte number restriction, resulting in multiple megaspores, and it was suggested that aposporous initials originate from these cells [57]. The gene is also involved in cell specification during the development of anthers. The GO terms 0009554: megasporogenesis and GO:0009556 microsporogenesis were both found also in the outlier genes of natural apomicts of *R. auricomus* [30] (Table 3). The finding of elevated dN/dS ratios in two genes in both F2 hybrids and in 70,000-year-old established natural apomicts support a hypothesis that a combination of mutations for sporogenesis (such as msp1) and dysfunctions of key meiosis genes (e.g., asy1) could be essential components for establishing apospory as a heritable trait. Competition of the megaspore with the aposporous initial cell, a neighbouring somatic cell that starts somatic embryo sac formation in parallel, might cause the final degeneration of the megaspore [58]. Six genes under diversifying selection in the hybrid–parent comparison are functionally annotated as transcription factors (172, 713, 3193, 3769, 3783, and 4129). Transcription factors are regularly observed to be under differential gene expression between apomicts and hybrids [12,24,59,60]. 

Among the genes under purifying selection in the hybrids, we found three genes related to GO term 0009556: microsporogenesis (contigs 3938, 3741, and 1905), which was also found in the established natural apomict *R. auricomus,* but among the outliers under diversifying selection [30]. Pollen development is not essentially altered in apomictic plants. The microgametophyte is meiotically reduced and remains functional in pseudogamous apomicts to fertilise the polar nuclei. However, increased rates of disturbances and the production of malformed pollen are regularly observed in *R. auricomus* [26,38] and in other genera [18,20,61]. The discrepancy between young and old aposporous lineages in dN/dS values for genes involved in pollen generation might be related to different ploidy levels. In the diploid F2 hybrids, the male haploid gametophyte would be under strong purifying selection as many genes are expressed [62], whereas, in the hexaploids, the reduced (triploid) pollen could carry mutated alleles to a higher extent as these would be masked from purifying selection by functional unmutated alleles [63]. For these genes, we suppose that indeed mutation accumulation over time (Muller’s ratchet) resulted in elevated dN/dS ratios in the hexaploids, without having a function for apospory. We found some genes under purifying selection that were reported in gene expression studies on sexual and apomictic *Boechera*, e.g., Cullin3A, the meiosis gene DYAD, and SGS3 (SUPPRESSOR OF GENE SILENCING 3) [64]. Interestingly, we did not find genes under selection that were found to be differentially expressed in a stage-specific microarray analysis of apomictic hexaploid *R. auricomus* lineages and their sexual parents [24]. Differential expression can rely on epigenetic control mechanisms or mutations in promoter regions, and would not necessarily require non-synonymous substitutions in exons. We further did not find any gene under selection reported for the artificial MiMe mutant system in *Arabidopsis* [14,65], suggesting that natural apospory evolved via a different genetic control mechanism.

We did not observe any of the known genes responsible for parthenogenesis to be under selection [12,66,67]. Since we sampled in the bud stage, they might have been not yet expressed, although the precocious development of apomictic ovules before anthesis has been reported for some genera [68,69,70]. However, the lack of genes related to parthenogenesis is also in accordance with the reproductive phenotypes of the F2 generation, which produced only c. 2% of apomictic seeds, and otherwise only sexually formed seeds [42]. Adding the parthenogenesis component would be essential to prevent further segregation and to inherit the apospory factor as a linkage block, as it appears in established apomictic *R. auricomus* lineages [37].

### 3.3. Scenarios for the De Novo Evolution of Apomixis in Diploid Hybrids

Our results corroborate previous findings that apospory, a major component of apomixis, originates in diploid plants [8] and can be inherited by haploid pollen [42,63]. However, the heritable factor for apospory [37] appears to be complex and probably includes a certain combination of mutations in key developmental genes. For the spontaneous emergence of apospory in F1 hybrids and the enrichment of apospory in F2 hybrids, different scenarios can be envisioned: non-synonymous substitutions emerged in key meiosis–sporogenesis genes in the F1 as novel alleles in the heterozygous condition, resulting in low proportions of aposporous ovules [38]. The F1 × F1 cross produced a segregating F2 generation with variable proportions of apospory and allelic dosage effects [26]. Alternatively, such mutations in key genes could have been already present as (undetected) polymorphisms in the sexual parental populations, but dispersed on different individuals/species, and therefore they remained without effect on the reproductive pathway of the respective sexual genotypes. Our filtering approach for parental polymorphisms was probably incomplete with the low number of samples used, and hence such polymorphisms might appear in the final loci of our parent–hybrid comparisons. A “lucky hybridisation event” of parental genotypes carrying these mutations combined these polymorphisms in the F1, resulting in the first appearances of apospory. Crosses of aposporous F1s produced transgressive segregants in the F2 with increased proportions of aposporous ovules. Further population genetic studies and mutagenesis tests would be required to discriminate between these hypotheses, but they are not mutually exclusive. Both scenarios would be in line with the rarity of the spontaneous emergence of apospory in natural hybrid systems. A third possibility would be the infectious origins of apospory by the pollen of polyploids carrying alleles for apospory, as observed in *Boechera* [63]. This scenario is unlikely in natural *R. auricomus* populations, because natural apomicts are mostly tetraploids and would produce diploid pollen with apospory factors fertilising haploid eggs of the diploid plants. The expected offspring would be triploid, but natural triploids are very rare in this complex [33].

In all scenarios, altered gene expression profiles of hybrids with ectopic or asynchronous gene expression under epigenetic control probably provide the systemic background fostering the aposporous pathway and the suppression of the default sexual pathway [11,13,53]. This epigenetic background is sensitive to environmental conditions, as the proportions of sexual ovule formation can be increased in facultative aposporous plants by abiotic stress [13,60,71,72]. However, it is an open question whether stress-induced epigenetic changes and gene expression profiles alone can constitute the heritability and long-term stability of apospory under natural conditions. Non-synonymous substitutions in key meiosis–sporogenesis genes, as traced here, might be required for the transgenerational transmission of apospory. Polyploidisation could play an important role by providing a better buffering of environmental stress, thereby downregulating the sexual pathway and hence indirectly establishing aposporous development in natural systems [72,73,74]. 

## 4. Materials and Methods

### 4.1. Plant Material

In order to study transcriptomic data, three wild *Ranunculus* parent plants and two synthetic F2 hybrids were chosen as plant material. Parent plants were collected from wild *Ranunculus* populations and were described as natural, allogamous diploid *R. carpaticola* and *R. notabilis* (Supplementary Table S1). *R. carpaticola* is nowadays regarded taxonomically as conspecific with *R. cassubicifolius* [35], but we keep here the original names as used in [26,42]. These plants were found to reproduce sexually with meiotic embryo sac formation (polygonum type development) and fertilisation of egg cells [38]. Using *R. carpaticola* and *R. notabilis* plants, manual crossing experiments were performed, which resulted in homoploid F1 plants with means of c. 11% aposporous ovules, 67% sexual ovules with functional megaspores, and the rest aborted ovules [38]. These F1 plants in turn served as parent plants for a second hybrid generation, which was produced between 2010 and 2012 (F, J plants; Supplementary Table S1; details in [42]). Genotyping, cytotyping, and morphological studies of this F2 showed that all plants were diploids, originated from sexual outcrossing, and markers segregated in a Mendelian fashion [42,47]. All plants formed aposporous ovules (with varying proportions), indicating that apospory factors were inherited from their parents [42]. From this F2 hybrid progeny, two individuals from hybrid classes F10 × F7 and J22 × J24 were selected that had been identified as diploids with high proportions of aposporous ovules (23 and 26%, respectively) [42]. In the present study, the original parent plants (see in [38]) could not be analysed due to their passing. Instead, between 2011 and 2018, *Ranunculus* individuals were recollected from the original *R. carpaticola* and *R. notabilis* populations [26]. All plants used in this study (Supplementary Table S1) were grown outdoors in pots in the old botanical garden of the Albrecht-von-Haller Institute for Plant Science at the University of Goettingen, Germany, under identical climatic and soil conditions. During development of flowering shoots and the collection of buds, plants were grown in climate growth chambers at equal, standardised conditions, as described [42]. 

### 4.2. RNA Extraction, Library Preparation, and Sequencing

Approximately 100 mg *Ranunculus* flower buds in all sizes were collected to represent all developmental stages from meiosis to the seed formation stage (before fertilisation) [30]. Buds were pooled and immediately frozen in liquid nitrogen. RNA extraction was subsequently done using the RNeasy Plant Mini Kit (Qiagen GmbH, Hilden, Germany), applying the manufacturer’s instructions, and total RNA concentration was determined with the Qubit 2.0 fluorometer (Invitrogen, Fisher Scientific GmbH, Schwerte, Germany). Library preparations and sequencing of the *Ranunculus* samples were done by the Integrative Genomics Core Unit of the University of Goettingen using an Illumina HiSeq 2500 platform (Illumina Inc., San Diego, CA, USA). Samples were pooled equimolar and sequenced paired-end, generating 250 bp reads.

### 4.3. Read Processing, De Novo Assembly, and Data Processing

The quality of the raw reads was evaluated with FASTQC v0.11.9 (https://www.bioinformatics.babraham.ac.uk/projects/fastqc/), while adapter sequence removal and quality trimming were done using the “palindrome mode” and the “sliding window quality filtering option” of TRIMMOMATIC version 0.36 [75]. Adapter sequence clipping was performed conservatively (ILLUMINACLIP: adapters.fa: 2: 30: 10: 8: keepBothReads) and accompanied by quality control (LEADING: 25 and TRAILING: 25; SLIDINGWINDOW: 8: 25). Trimmed reads with a length below 90 bp were removed (MINLEN: 90).

Due to the absence of a reference genome, trimmed *Ranunculus* reads were de novo assembled individually per sample using rnaSPAdes [76], which is suitable for reference-based as well as for de novo transcriptome assemblies. The tool has shown its predominance in terms of isoform detection, successful gene assembly, and read-length-based k-mer size calculations, while at the same time generating low ratios of misassemblies and duplication [76]. BUSCO version 3.0.2 was subsequently applied to analyse the quality of the *Ranunculus* transcriptomes by checking the completeness and fragmentation rate of a “core” of single-copy orthologous genes of data package “Viridiplantae” [77]. 

### 4.4. Hybridisation Networks

As the parental individuals of the F2 samples expired prior to the beginning of this study, we evaluated the relationships between the three individuals representing the parental populations and the two F2 hybrids by hybridisation networks. Maximum likelihood gene trees were generated from the alignments used for dN/dS analysis using RAxML-NG [78]. Minimum hybridisation networks were computed using the gene trees as input and the autumn algorithm [79] implemented in Dendroscope 3 [80].

### 4.5. Analyses of Loci under Selection (dN/dS Ratio Analyses)

Orthologous open reading frames in the consensus sequences of assembled contigs were identified using ProteinOrtho v6.0.14 [81], which implements reciprocal best alignment heuristics to identify orthologous single-copy regions. The output was curated to retain only putative single-copy orthologues recovered in all five individuals. Transdecoder v5.5.0 (https://github.com/TransDecoder) was used to identify the longest open reading frame (ORF) per consensus sequence, discarding any sequences shorter than 300 bp (100 AA). For each putative orthologue, the identifier representing the gene in the respective samples was collected from the ProtheinOrtho output and the assorted ORF protein sequence written to a fasta file using a custom python script (ProtheinOrtho2Fasta.py). Sequences were aligned using MAFFT v7.304 [82] with the options adjust direction, localpair, and maxiterate 100, and back-translated using RevTrans v1.4 [83]. As Transdecoder reports cds sequences including stop codons, but RevTrans fails when they are present, stop codons were removed (remove_stops.py) prior to back-translation.

A majority rule consensus sequence excluding gaps represented each gene for functional annotation using Trinotate v3.2.1 (https://github.com/Trinotate/Trinotate.github.io) with BLASTP [84] against the UniProt Database (2021_11 release; https://www.uniprot.org). Contigs annotated as non-spermatophyte were removed as putative contaminants [85]. Reference genes matching different contigs were identified as putative paralogues and the contigs were removed. Contigs matching multiple reference genes were assessed for overlap. If the mapped fragments of the reference genes overlapped, the contig was removed. If no overlap was detected, the contig was included and subjected to further analysis.

To estimate the heterozygosity of each locus, for each sample, the trimmed Illumina reads were mapped to the consensus sequences of individual loci. The *mpileup* command as implemented in SAMtools v.1.9 [86] was used to extract the base calls of mapped reads per position. The information was passed to a custom python script that counted the number of homozygous or heterozygous positions in the sequence of a locus. A position was considered heterozygous if the minor base call was recovered in at least 10% of the read depth to exclude erroneous base calls. Accordingly, the percentage of heterozygous sites per locus and sample was assessed. 

To evaluate whether DNA sequences were variable within the two groups (F2 hybrids and parental representatives), we compared sequences within the groups for each alignment, assessing whether sequences were identical or not. The results were compared between groups and, in addition, differentiating within the parental representatives (Venn diagrams, Supplementary Figure S1).

### 4.6. Filtering dN/dS Ratios

To estimate general dN/dS ratios, we identified the longest in-frame, ungapped section of at least 30 AA in the alignment across all samples for each single-copy locus. We calculated average dN/dS ratios per locus, as well as dN/dS ratios, for all sequence pairs using the Bioperl Bio:Align:Statistics module [87], employing the Jukes–Cantor substitution model and the Nej–Gojobori algorithm. To avoid division with a zero in the equation in cases where there were either no synonymous or no non-synonymous substitutions detected, we added a small constant of 0.01 to each estimated value [46]. The dN/dS values for all 1514 loci for all ten pairwise comparisons are shown in Supplementary Figure S3 and were used to set thresholds for further filtering steps (Table 2). To avoid considering divergent loci in the parental species that would be heterozygous in the F1 and segregate in the F2 hybrids, resulting in false positive substitutions in parent–hybrid comparisons, we removed all loci with a dN/dS value > 1.0 in the parent–parent comparison. Furthermore, we removed all loci with a dN/dS value > 1.0 between the two *R. notabilis* samples to reduce parental intraspecific polymorphisms. Finally, we removed all genes with a dN/dS value > 4.0 in the hybrid–hybrid comparison to narrow down to genes that were shared by both hybrid samples (as apospory is a shared trait). From this subset, we selected all genes with dN/dS > 1.0 in hybrid–parent comparisons, i.e., representing best the actually diversifying loci between sexual parents and aposporous F2 (79 annotated genes; Supplementary Table S5). 

### 4.7. Gene Ontology

For genes inferred to be under positive selection in hybrids compared to parents, Gene Ontology (GO) was assessed. GO terms listed in the Trinotate annotation do not comprise the entire trajectory across the GO tree for a given term, but instead several of the lowest level entries uniquely identifying a gene’s ontology for all of the three main categories (biological process, molecular function, cellular component). These entries permit the traversal of the hierarchy backwards towards the highest levels to obtain a complete ontology for any given gene. A custom script (GO_breakdown.py) using the package goatools [88] implemented this process of obtaining the GO terms from the Trinotate annotation, assorting them into the three overall categories and obtaining the higher-level terms from the Gene Ontology database (2021_11 release) per gene and summarising results. All scripts can be found in the GitHub repository: https://github.com/ClaudiaPaetzold/MEME_sum_and_annot.git.

In addition, we collected GO terms linked to reproduction, specifically GO:0048229: gametophyte development, GO:0000003: reproduction, GO:0022414: reproductive process, GO:1903046: meiotic cell cycle process, GO:0048236: plant-type sporogenesis, and GO:0000741: karyogamy, including all their respective child terms, and investigated whether these were present in the sets of genes under selection. We assigned the subset of GO terms related to reproduction into five stage-specific categories: (1) flower and ovule formation, (2) meiosis, (3) embryo sac development and gametogenesis, (4) embryo and seed formation, and (5) male functions (pooled over stages).

## Figures and Tables

**Table 1:** Summary of Illumina sequencing, quality control of reads, and assembly for the five *Ranunculus* samples herein.

	*R. notabilis* 10137-3	*R. notabilis* 10137-8	*R. carpaticola* P15(02)	F2 Hybrid C4	F2 Hybrid F10
Number of raw reads	3,878,575	3,868,199	2,754,320	5,610,385	2,797,829
Number of trimmed reads	3,750,852	3,815,142	2,724,406	4,487,452	2,774,750
Number of assembled contigs	28,520	30,262	25,501	33,233	27,789
Average contig length (bp)	812.59	778.60	756.48	823.97	765.97
Longest contig (bp)	10,473	10,008	11,019	11,019	7131
Shortest contig (bp)	300	300	300	300	300

**Table 2:** Results of the filtering of loci according to dN/dS values for parental divergence and intraspecific and inter-hybrid divergence. The remaining loci were filtered for parent–hybrid dN/dS < 1.0 and finally for annotated loci.

Levels of dN/dS Values of 1514 Loci in Pairwise Comparisons	No. Loci Filtered	%
Parental divergence (average of 2 pairs, dN/dS > 1.0)	139	9.18%
Intraspecific polymorphism in *R. notabilis* (1 pair, dN/dS > 1.0)	81	5.35%
Hybrid–hybrid divergence (1 pair, dN/dS > 4.0)	20	1.32%
Parent–hybrid comparison (average of 6 pairs, dN/dS < 1.0)	1176	77.68%
Parent–hybrid comparison not annotated (average of 6 pairs, dN/dS > 1.0)	19	1.25%
Parent–hybrid comparison annotated (average of 6 pairs, dN/dS > 1.0)	79	5.22%
Total no. of loci	1514	100.00%

**Table 3:** Functional annotation of putative single-copy genes subjected to diversifying selection in pairwise comparisons between the F2 hybrid and the parental individuals, which are linked to reproduction with the coordinates of the coded proteins in the transcript. The functional annotation based on UniProt is given with the symbol and organism of the reference gene, and the most general (top-level) GO term(s) for their functional involvement in the reproductive process. Reference organism: ARATH: *Arabidopsis thaliana*. ORYSJ: *Oryza sativa japonica.* GO terms also found by [30] under strong diversifying selection between apomictic and sexual *R. auricomus* lineages are marked in bold.

Contig	Protein Coordinates	Symbol	Function	GO Association
contig_1127	1–978 [+]	THA8_ARATH	THYLAKOID ASSEMBLY 8, chloroplastic	
				GO:0009793 embryo development ending in seed dormancy
contig_1423	1–921 [+]	XRI1_ARATH	X-ray-induced transcript 1	
				GO:0007143 female meiotic nuclear division
				GO:0007140 male meiotic nuclear division
				GO:0009555 pollen development
contig_145	1524–3197 [+]	APC1_ARATH	Anaphase-promoting complex subunit 1	
				GO:0009793 embryo development ending in seed dormancy
				GO:0009553 embryo sac development
				GO:0007091 metaphase/anaphase transition of mitotic cell cycle
				GO:0048481 plant ovule development
contig_213	2–1033 [+]	ADT4_ARATH	ADP-ATP carrier protein ER-ANT1	
				GO:0048316 seed development
contig_3193	242–916 [+]	DIV_ANTMA	Transcription factor DIVARICATA	
				GO:0009908 flower development
contig_3535	242–916 [+]	MSP1_ORYSJ	Leucine-rich repeat receptor protein kinase MSP1	
				GO:0048658 anther wall tapetum development
				**GO:0009554 megasporogenesis**
				**GO:0009556 microsporogenesis**
contig_3826	1–384 [+]	NOC4_ARATH	Protein NUCLEOLAR COMPLEX ASSOCIATED 4	
				GO:0009793 embryo development ending in seed dormancy
contig_4136	1–1857 [+]	ASY1_ARATH	Meiosis-specific protein ASY1	
				**GO:0007129 homologous chromosome pairing at meiosis**
contig_52	919–3012 [+]	SYVM2_ARATH	Valine—tRNA ligase, chloroplastic/mitochondrial	
				GO:0009793 embryo development ending in seed dormancy
contig_583	1–1032 [+]	SC15B_ARATH	Exocyst complex component SEC15B	
				GO:0060321 acceptance of pollen
				GO:0009846 pollen germination
				GO:0009860 pollen tube growth

## Data Availability

Raw Fastq files for all five samples are available on the SRA archive under BioProject Number PRJNA870679. All custom scripts are available in the GitHub repository: https://github.com/ClaudiaPaetzold/dndsRanunculus.git.

## Supplementary Materials

The following supporting information can be downloaded at: https://www.mdpi.com/article/10.3390/ijms232213881/s1.
