# Supplementary material for: Evolution of Transcriptomes in Early-Generation Hybrids of the Apomictic Ranunculus auricomus Complex (Ranunculaceae)
Source: Int J Mol Sci. 2022 Nov 10;23(22):13881. doi: 10.3390/ijms232213881 (PMC9697309; doi:10.3390/ijms232213881)
Supplement: Supplementary file 1 [file ijms-23-13881-s001.zip › Supplemental Table S3_heterozygosity.pdf]

Supplemental Table S3. Heterozygosity. Percentage of heterozygous sites per locus for the ingroup samples including the average across loci for each sample. Loci inferred to be subject to diversifying selection in pairwise comparisons and which a linked to the reproductive process are written in bold.

	10137-3	10137-8	C4-21	F10-F7	P15-02
contig_1009	1.10294118	1.63562017	0.94868478	1.63511188	1.0989011
contig_1010	1.77595628	3.94557823	3.42935528	3.2	1.4986376
contig_1011	3.30788804	1.36612022	2.08978328	4.78527607	4.03726708
contig_1012	1.44478844	3.19917441	2.99500832	1.54798762	2.06398349
contig_1017	1.30597015	3.6163522	2.59128386	2.56124722	2.55839822
contig_1019	1.10062893	2.58136925	3.36538462	1.4527845	6.34920635
contig_101	1.03780578	0.89485459	1.58572844	1.45038168	0.57915058
contig_1020	1.55677656	3.08810173	3.09859155	5.20340587	1.79584121
contig_1021	2.17687075	4.21768707	5.68475452	1.9047619	1.10192837
contig_1022	1.78343949	3.25925926	0.59435364	2.81007752	0
contig_1024	1.73796791	2.06666667	1.93333333	2.49140893	2.3364486
contig_1025	0.98253275	3.29087049	2.71398747	4.80349345	3.64145658
contig_1028	1.39470014	2.10772834	4.31654676	2.65095729	2.95950156
contig_1029	0.9118541	1.35951662	1.88964475	0.52521008	2.18181818
contig_102	1.01088647	2.57048093	1.47286822	3.58422939	2.41874528
contig_1030	6.81818182	8.38574423	7.94520548	8.55762595	7.1230982
contig_1031	1.38476756	1.77613941	1.45489816	1.69875425	1.89530686
contig_1032	0.62780269	2.32350313	3.22997416	1.87332739	1.25786164
contig_1033	2.00174064	2.82331512	2.41666667	1.06899903	2.76953511
contig_1036	2.03804348	2.23004695	2.69953052	1.83968463	0.75566751
contig_1038	1.44927536	3.80147835	2.15384615	2.04678363	1.65530672
contig_1039	1.96687371	2.89855072	1.53597413	1.80084746	1.89054726
contig_1046	1.55251142	2.65567766	1.92307692	1.07816712	4.3040293
contig_1047	1.37875101	3	2.97188755	2.14067278	0.81900082
contig_1049	1.63132137	1.29783694	1.31276666	1.67029775	2.06540448
contig_104	1.11464968	1.47579693	1.06508876	2.8340081	1.00294985
contig_1050	0.50055617	1.76948828	1.67247387	1.94741967	0.97431355
contig_1051	1.26582278	3.49794239	1.26315789	0.9771987	1.3584117
contig_1053	1.81623932	1.80555556	2.96896086	1.91256831	1.34874759
contig_1055	0.55161545	3.06242638	4.11522634	3.69369369	1.01483216
contig_1059	1.17056856	1.68970814	1.83946488	3.05010893	0.98743268
contig_105	7.45957225	8.42639594	4.07040704	6.24315444	7.74448841
contig_1061	2.98742138	1.21212121	3.09050773	2.66903915	0.66666667
contig_1062	0.47233468	1.91256831	0.89041096	1.19047619	1.58415842
contig_1064	0.76230076	2.48962656	1.09439124	1.65289256	1.77304965
contig_1069	4.63768116	3.27613105	3.19444444	3.13479624	4.2042042
contig_1077	2.55962769	2.56410256	3.95189003	3.13199105	1.04347826
contig_1079	1.78671133	2.95924065	2.5083612	1.64690382	0.94602115
contig_107	1.53349475	2.23684211	1.6762703	3.1120332	0.66666667
contig_1083	2.90909091	2.55591054	4.11826822	6.21118012	4.66360856
contig_1084	0.62794349	1.88124633	1.58610272	1.86615187	1.10847189
contig_1085	0.73359073	3.56435644	1.90912562	1.37352156	0.43236566
contig_1086	0.68153656	1.26662445	1.26502214	1.69172932	0.81607031
contig_1087	0.80758427	0.97919217	1.42810776	0.39196472	1.23745819

contig_1088	0.72992701	2.23577236	3.80517504	1.89035917	2.33393178
contig_1089	1.03244838	2.35042735	3.08056872	0.89552239	1.64056674
contig_1092	1.57325467	3.15068493	2.00980392	2.2971652	1.42576205
contig_1093	1.62381597	2.77777778	2.96296296	1.82046453	1.34874759
contig_1094	1.1687363	0.89047195	1.72488141	1.51187905	1.3739546
contig_1095	2.41545894	3.73216246	2.88153682	4.42477876	2.22929936
contig_1096	2.86677909	1.85185185	2.72669626	0.98146129	1.44654088
contig_1098	0.95144357	1.76211454	0.96153846	0.68027211	0.74753653
contig_10	1.11706881	2.36886633	1.6286645	1.09519798	0.91012514
contig_1102	3.05232558	5.26315789	3.29457364	1.2802276	1.56657963
contig_1103	1.03668262	1.51515152	1.75019889	1.03668262	1.59509202
contig_1104	1.01936799	2.13414634	2.63424519	1.13821138	1.21334681
contig_1105	1.6	1.38539043	1.37142857	7.05882353	0.9184845
contig_1106	0.95367847	1.94444444	2.141527	1.5625	2.7938343
contig_1108	0.61383929	1.88583078	1.3740458	1.84710108	1.25074449
contig_110	0.99750623	1.33333333	1.77777778	1.31826742	1.13507378
contig_1110	0.59737157	1.20082816	1.64	1.11642743	0.77720207
contig_1111	1.02432778	2.8340081	2.20492866	1.72642762	2.19966159
contig_1114	0.43103448	1.99409158	1.12781955	2.18579235	0.95735422
contig_1115	1.64319249	2.04220558	2.15924426	1.3582343	1.49812734
contig_1118	3.80313199	1.78117048	4.16107383	0.89186176	3.47448426
contig_1119	2.20750552	3.45080764	3.010279	2.33609646	2.29885057
contig_1121	1.23886953	1.74621653	1.75195943	1.04543627	1.02150538
contig_1124	2.15189873	2.78128951	1.25698324	0.63131313	0.90909091
contig_1125	1.92165558	4.19426049	2.28782288	5.01474926	0.73349633
contig_1127	1.84397163	2.02177294	3.54609929	2.13068182	3.29436769
contig_1128	3.07692308	4.38931298	5.67375887	3.10077519	2.30769231
contig_112	2.09380235	1.22377622	3.10663308	2.26700252	2.10260723
contig_1130	0.71942446	2.01660735	1.72839506	2.56081946	3.48837209
contig_1131	2.35507246	3.5440613	3.97470641	1.46118721	1.73482032
contig_1136	0.70175439	1.32013201	2.7027027	0.86805556	0.38610039
contig_1137	3.93939394	4.54545455	5.35279805	4.89296636	4.54545455
contig_1138	5.02392344	3.3492823	4.69371519	2.71132376	0.95465394
contig_1139	7.85659802	8.23798627	8.23798627	8.82800609	8.00915332
contig_113	1.31421744	1.81639666	1.19474313	1.60726765	1.92455735
contig_1140	3.35195531	2.43445693	2.24299065	1.73410405	1.35135135
contig_1141	0.98461538	0.50314465	1.41829394	1.14966126	0.55096419
contig_1142	1.00502513	3.78710338	1.20603015	1.5060241	0.71283096
contig_1143	1.79487179	2.31958763	3.36787565	4.92227979	3.36787565
contig_1144	0.74294205	1.38682516	0.89153046	1.63447251	0.64388311
contig_1146	0.71081182	1.01275319	0.9734182	0.63837777	1.06434446
contig_1147	1.53110048	1.6424214	1.55148096	0.43165468	0.60947023
contig_1149	3.76344086	3.79403794	3.22580645	2.95698925	3.25203252
contig_114	1.51515152	1.43084261	2.41545894	0.19607843	1.43084261
contig_1151	1.89437428	2.73828331	2.15538847	1.60481444	0.65261044
contig_1155	3.03030303	3.33333333	4.8714479	3.03951368	3.14842579
contig_1156	1.18343195	1.98537095	1.03652517	1.75534833	2.07202763
contig_1159	1.34357006	3.28638498	2.85714286	2.28571429	2.47619048
contig_115	1.38955438	2.73512476	1.94741967	0.7183908	6.82800346

contig_1161	2.39410681	2.9143898	3.62318841	3.26086957	2.36794171
contig_1162	0.80606923	1.18539592	1.56472262	1.60662123	0.82484231
contig_1163	1.05263158	3.43796712	2.84005979	1.34529148	2.24887556
contig_1165	1.12517581	2.25035162	1.54711674	1.96905767	1.96078431
contig_1166	0.76992754	1.50204825	0.99682827	1.25581395	0.86956522
contig_1168	1.22204289	1.76515733	1.07009096	0.68859985	0.76785257
contig_116	1.09503324	1.47798742	1.07482431	0.88282504	1.48560817
contig_1170	2.44565217	1.7699115	4.83870968	1.88172043	1.07238606
contig_1173	4.76190476	3.23383085	3.50877193	4.72636816	4.97512438
contig_1174	1.59680639	3.59281437	4.16666667	1.58730159	2.77777778
contig_1175	1.10650069	2.22222222	2.7027027	0.61443932	1.74672489
contig_1179	2.35690236	3.03030303	3.76811594	3.20945946	2.82392027
contig_1180	1.45867099	2.59319287	2.43111831	1.29449838	1.61812298
contig_1183	1.55195682	2.0242915	1.75438596	1.01214575	0.47201618
contig_1185	0.81607031	1.69491525	1.4438167	1.4438167	0.81607031
contig_1186	0.26730821	1.19885327	1.04278075	1.79738562	0.80792286
contig_1187	10.3626943	10.9730849	12.1649485	12.4681934	11.9496855
contig_118	1.8627451	1.8018018	1.96850394	2.52742012	2.17948718
contig_1191	1.40913508	2.21260221	1.87654321	2.37203496	2.75132275
contig_1192	1.44462279	2.24	2.08333333	2.39616613	1.3559322
contig_1195	2.47252747	2.91005291	3.41207349	2.88713911	2.36842105
contig_1197	2.08333333	2.60610573	2.55892256	0.62849162	2.42424242
contig_1199	1.15979381	2.20673635	2.10526316	1.07526882	0.84541063
contig_11	2.73584906	1.81979583	1.87553282	2.7173913	1.46137787
contig_1200	2.17765043	3.13238771	2.83687943	0.9163803	1.66093929
contig_1201	1.61812298	2.14884696	1.81229773	0.94339623	1.20734908
contig_1207	0.92449923	1.55563071	1.55925156	1.99724518	2.36686391
contig_1208	3.41726619	4.40044004	4.42583732	3.13225058	3.63636364
contig_120	1.14155251	2.85996055	1.54162384	1.31264916	1.55382908
contig_1211	7.21393035	6.3670412	5.26315789	5.00610501	1.98830409
contig_1216	0.43252595	1.78381257	1.09684323	0.92929293	1.03550296
contig_1217	1.86125212	3.33333333	2.81923715	2.51968504	1.32231405
contig_1218	1.44144144	2.34234234	2.3465704	1.64271047	1.62162162
contig_1219	3.14685315	4.07925408	2.27531286	3.03030303	2.91970803
contig_121	4.10677618	5.27497194	2.49221184	2.66875981	2.97029703
contig_1220	1.92926045	2.35798499	1.82795699	0.64308682	0.98360656
contig_1223	0.6655574	1.38657793	0.88740987	1.10926234	0.72102052
contig_1224	2.77275467	2.65435661	3.78531073	1.57004831	1.07526882
contig_1229	2.94715447	5.10855683	3.47923681	1.91158901	2.74122807
contig_1231	7.38738739	7.05244123	6.88405797	7.74774775	5.04504505
contig_1232	1.1778563	1.57004831	1.75438596	1.17370892	2.38948626
contig_1233	2.21187427	3.28638498	9.00321543	1.12485939	0.58892815
contig_1234	1.74029451	2.43243243	2.02898551	0.66137566	1.43415906
contig_1235	1.64670659	2.31689088	1.87125749	1.56950673	2.58215962
contig_1237	1.27226463	0.94857713	1.4242116	2.13823968	3.28358209
contig_1238	0.90865615	1.63447251	1.08964834	0.39741679	0.89463221
contig_1240	5.76441103	5.68475452	7.07892596	6.46464646	1.90796857
contig_1241	0.77808901	0.93370682	0.62266501	1.7571885	1.12866817
contig_1242	4.67032967	2.39680426	2.26666667	1.22116689	2.17096336

contig_1244	2.39520958	3.79241517	3.99201597	2.59481038	2.99401198
contig_1247	2.21861472	3.19559229	3.61380798	1.08225108	2.62438491
contig_1249	3.46153846	2.59259259	1.96905767	1.10701107	1.10041265
contig_1250	1.71428571	2.87356322	3.98860399	3.7037037	2.19435737
contig_1251	1.2987013	0.57803468	0.43731778	1.04821803	1.7366136
contig_1252	2.86576169	4.97737557	4.05405405	1.2012012	2.14067278
contig_1253	3.51421189	4.77642276	5.75835476	3.3106961	3.43213729
contig_1256	1.96969697	3.7037037	4.07239819	2.26586103	0.75528701
contig_1257	0.51502146	1.28755365	0.8583691	0.43972151	1.28902893
contig_1258	3.04232804	3.17460317	3.57142857	2.54350736	2.124834
contig_1259	1.99004975	2.19560878	2.29226361	2.11480363	1.12359551
contig_1261	0.92470277	1.35135135	2.04230489	0.88996764	0.78563412
contig_1262	1.90114068	3.33778371	4.19753086	1.63043478	1.94444444
contig_1263	1.89003436	2.86532951	3.09278351	2.07253886	2.24525043
contig_1265	3.09789343	2.4968789	2.734375	1.64141414	0.65104167
contig_1266	1.22199593	2.21327968	2.04498978	0.8988764	0.41237113
contig_1267	1.39958013	2.87316048	2.10526316	1.42215569	0.67114094
contig_1268	1.16110305	1.62002946	1.70212766	1.04947526	0.8
contig_126	1.36518771	2.99901672	2.68656716	0.6385696	1.52542373
contig_1272	3.117506	3.40632603	2.87769784	1.65484634	2.15827338
contig_1274	0.84557187	0.99103351	1.08286252	0.62409986	0.41570439
contig_1275	2.04429302	2.09545984	1.98135198	3.15052509	2.96208531
contig_1277	2.89435601	6.55270655	4.98442368	2.65486726	4.27728614
contig_1278	0.28422549	1.07879925	1.64088139	1.05973025	0.72498792
contig_127	1.75879397	3.67892977	3.97849462	1.44144144	2.24867725
contig_1281	1.25984252	3.34672021	1.88679245	2.78884462	3.67346939
contig_1282	1.9525802	5.1558753	2.6874116	0.93457944	3.68020305
contig_1290	0.8974359	3.74677003	3.47222222	3.26797386	1.25
contig_1295	1.8018018	1.21396055	1.95530726	1.36778116	0.76452599
contig_1298	1.83150183	2.53748558	2.29357798	1.72839506	1.73482032
contig_1299	3.53817505	2.97951583	2.79329609	2.52252252	1.47058824
contig_129	0.75390415	1.83776933	1.73292559	1.76565008	1.53508772
contig_1300	0.87719298	3.67647059	4.19847328	1.74216028	0.60790274
contig_1301	1.05263158	2.60972716	3.21805956	0.63492063	2.07223209
contig_1302	1.14068441	1.76100629	2.3989899	1.25786164	1.12781955
contig_1304	0.93374833	1.46780303	1.10619469	0.8355321	0.66401062
contig_1305	1.51946819	2.1319797	3.49702381	2.24948875	0.6833713
contig_1308	4.61309524	1.92592593	5.8224163	6.35400908	6.42201835
contig_130	3.62158167	6.28415301	3.50727117	2.71398747	2.17717718
contig_1310	1.51653018	1.61896502	3.24532453	1.54177124	1.81518152
contig_1311	0.8782936	1.86335404	0.88832487	1.85414091	2.25988701
contig_1312	4.76190476	10.6606607	5.3652968	1.17096019	5.78512397
contig_1313	0.65320665	2.01302546	1.71394799	1.30331754	0.82889284
contig_1318	3.04878049	1.96581197	2.22222222	0.94017094	1.36752137
contig_1320	1.19760479	2.7203482	2.0687354	1.0707635	0.43454644
contig_1323	1.87265918	3.1835206	2.43445693	2.62172285	2.43445693
contig_1324	2.29885057	2.50626566	3.04568528	3.1372549	1.64158687
contig_1325	1.67958656	2.85006196	2.71940667	1.50943396	2.49671485
contig_1326	2.62793914	2.76625173	2.21300138	2.35131397	2.21300138

contig_132	0.93632959	6.89655172	2.78086763	4.93119266	7.34966592
contig_1331	0.57471264	0.93599034	0.54380665	0.44692737	1.08378491
contig_1333	1.29982669	1.53452685	2.73738238	1.03896104	3.39393939
contig_1337	0.74801309	0.79476391	0.88578089	0.6097561	1.21552127
contig_1338	1.34680135	1.90796857	2.69360269	1.31108462	1.62601626
contig_1339	2.56410256	3.41880342	4.19161677	2.56410256	2.77777778
contig_133	2.40811153	4.81481481	9.88593156	3.07298335	5.32212885
contig_1341	1.70940171	2.05128205	1.70940171	1.88034188	1.22591944
contig_1342	1.9969278	3.67553866	3.37941628	2.35439901	4.04040404
contig_1345	3.31890332	3.49854227	2.5	1.59883721	2.71084337
contig_1347	0.96491228	1.35964912	0.92105263	1.81691125	0.56131261
contig_1348	1.32370638	1.48975791	1.14320096	1.94003527	1.32450331
contig_1349	12.7272727	14.0096618	13.5135135	10.8108108	12.9268293
contig_1351	1.10650069	1.79806362	1.6	1.96078431	2.27920228
contig_1352	2.24299065	2.33174351	3.1377899	0.86520947	1.46551724
contig_1355	2.20125786	3.77358491	3.77358491	1.42405063	3.30188679
contig_1356	2.21300138	3.18118949	3.06834031	1.38312586	2.45310245
contig_1358	3.87096774	2.29885057	2.44252874	0.92250923	1.73160173
contig_1359	0.72320842	1.27811861	1.43149284	1.89161554	1.38461538
contig_135	0.83022001	2.94985251	0.50590219	0.99789916	2.37226277
contig_1361	0.969163	1.4379085	1.56903766	1.5971606	1.58227848
contig_1362	2.5998143	1.70068027	3.07429547	2.21774194	1.84659091
contig_1363	1.20634921	2.81345566	1.51202749	3.37150127	1.09965636
contig_1365	2.69151139	4.10509031	4.43349754	1.18443316	2.09339775
contig_1366	1.60714286	1.76125245	3.99305556	0.70546737	1.2987013
contig_1367	2.21169036	2.42792109	2.75761974	3.07219662	3.1152648
contig_136	3.33333333	3.09901738	1.32939439	3.60934183	1.59509202
contig_1370	0.90957731	1.12359551	0.90957731	0.53504548	0.74946467
contig_1371	2.76679842	2.13618158	2.89308176	0.79260238	2.88220551
contig_1372	1.32275132	1.64021164	1.37566138	0.69002123	1.63719712
contig_1374	3.22580645	3.085554	2.94117647	1.73796791	2.38095238
contig_1377	2.43902439	2.28571429	2.01096892	2.69749518	5.5028463
contig_1378	2.29276896	5.88235294	5.82010582	4.09982175	1.62601626
contig_1379	0.83333333	0.57388809	1.97268589	1.48809524	1.68776371
contig_137	1.86011905	2.80991736	2.30141054	2.58112094	1.30081301
contig_1382	1.95473251	3.12185297	2.72536688	2.62054507	4.05797101
contig_1383	0.73138298	1.21446315	0.84257206	1.0955414	0.98522167
contig_1387	1.03578154	1.44578313	1.34874759	1.13475177	0.88845015
contig_1389	4.16666667	7.37704918	7.07964602	3.79403794	1.80878553
contig_138	0.20408163	0.4494382	1.26715945	1.34680135	1.0940919
contig_1393	0.1424276	0.93141406	1.02636981	0.46544934	1.28700129
contig_1394	1.40986908	0.95669688	0.65458207	1.20845921	0.91139241
contig_1395	2.11382114	2.12765957	3.08943089	2.45098039	2.31788079
contig_1398	2.03160271	2.7027027	4.05405405	1.12866817	3.9039039
contig_139	2.70498732	2.90697674	3.76068376	2.28813559	2.26950355
contig_13	7.08401977	2.43055556	5.57532622		2.65251989
contig_1400	0.8517228	1.09151973	0.92592593	1.01641908	0.69670228
contig_1401	0.60015004	1.22530633	4.72618155	1.02525631	3.81395349
contig_1404	0.66666667	1.38461538	1.23685838	1.64355419	0.41025641

contig_1406	1.26182965	3.26487625	1.62218734	1.72684458	1.57728707
contig_1407	0.96818811	4.70430108	5.53250346	3.0428769	1.2465374
contig_1408	0.12437811	1.19205298	1.80505415	2.05562273	1.21212121
contig_1409	1.31386861	3.21637427	3.34788937	2.7696793	3.45303867
contig_140	1.52505447	3.48047538	2.03327172	1.68776371	0.85910653
contig_1412	3.00429185	3.00429185	3.29041488	1.57367668	4.00572246
contig_1413	1.57021086	2.08333333	2.24315837	1.3016158	0.53859964
contig_1416	1.74672489	4.08163265	2.91120815	1.73796791	2.19619327
contig_1418	1.57480315	4.34782609	4.26008969	3.04154303	2.42305174
contig_141	0.61396777	2.67857143	1.64056674	2.3653088	0.69444444
contig_1421	2.417962	0.81168831	0.91991342	1.91152376	1.30010834
**contig_1423**	**1.34378499**	**1.97802198**	**2.87929125**	**1.99556541**	**0.51457976**
contig_1424	1.21546961	2.24466891	3.07692308	1.25142207	1.46878825
contig_1428	1.64102564	0.87719298	1.82025028	1.81043663	1.24293785
contig_142	0.95396101	1.20980579	1.05519481	1.44470712	0.98784195
contig_1430	1.54211151	3.67734282	3.19148936	1.70940171	0.38560411
contig_1431	1.5576324	2.22222222	1.40625	2.02492212	1.92837466
contig_1432	1.36986301	1.05421687	1.92592593	1.47783251	1.67427702
contig_1435	2.29885057	3.59195402	2.77777778	1.86781609	2.29885057
contig_1436	2.06812652	2.43753809	2.24242424	1.39103555	1.68471721
contig_143	0.7079646	0.97465887	2.11081794	1.26682502	0.52910053
contig_1440	1.70212766	4.11160059	2.99145299	1.75438596	3.28849028
contig_1441	1.73199635	1.74165457	1.39664804	1.80995475	1.02389078
contig_1448	2.40334378	1.96353436	2.30769231	0.8372093	2.12014134
contig_1451	1.46699267	1.93103448	3.40136054	1.36798906	1.2244898
contig_1452	2.38095238	3.44827586	2.60047281	2.93333333	1.72839506
contig_1453	0.68277311	1.00697134	1.26614987	1.26368997	0.50707232
contig_1454	3.9800995	4.36241611	4	4	2.83806344
contig_1456	2.34972678	2.46305419	2.29885057	2.1369863	2.29257642
contig_1458	2.23463687	4.21245421	3.61445783	3.23232323	1.30111524
contig_1459	1.9138756	5.58213716	2.3923445	2.8708134	3.66826156
**contig_145**	**1.10529378**	**5.16473731**	**1.79399938**	**2.55220418**	**1.00791937**
contig_1460	2.72988506	2.49406176	1.11248455	1.31894484	1.36239782
contig_1461	2.38379023	1.61691542	2.72614622	1.99004975	1.62703379
contig_1464	1.2802276	1.65016502	1.70454545	1.04314841	0.9009009
contig_1465	0.88202867	1.45985401	0.87336245	1.56622404	0.76726343
contig_1466	2.48366013	2.72108844	1.90735695	2.04081633	1.24137931
contig_1467	1.2195122	2.16606498	2.03873598	1.54958678	3.57142857
contig_1468	1.54958678	1.61616162	1.41414141	1.328125	1.26262626
contig_1469	0.92592593	1.19825708	1.52505447	1.30861505	1.19825708
contig_146	2.13523132	3.79746835	1.91780822	1.45190563	0.52128584
contig_1470	2.53333333	2.34234234	3.06306306	2.65848671	2.52252252
contig_1471	11.9601329	15.1666667	14.0961857	14.8900169	13.2996633
contig_1474	8.50574713	7.5862069	8.27586207	8.73563218	7.37327189
contig_1478	1.13821138	1.78861789	1.57266811	1.62601626	0.92140921
contig_147	1.50784077	2.5	3.19803198	2.56410256	2.87921348
contig_1480	3.22580645	4.53752182	5.42635659	2.75080906	2.55255255
contig_1483	4.21369451	2.63951735	3.46907994	3.09200603	1.72910663
contig_1485	1.67064439	3.00925926	3.08056872	5.16431925	2.11764706

contig_1486	0.87064677	2.17256363	1.86219739	2.83524904	1.19271814
contig_1488	1.32978723	1.11642743	1.5625	1.3318535	0.75716604
contig_1489	1.38408304	2.65957447	2.46045694	2.4822695	1.8018018
contig_1491	1.88014101	1.6509434	1.08024691	2.13740458	0.98159509
contig_1493	0.90735435	1.33715377	1.52817574	1.7669532	0.5730659
contig_1495	2.76301807	2.52469813	2.69005848	2.06297503	1.29032258
contig_1496	1.78571429	2.88065844	3.29218107	1.12359551	2.47252747
contig_1499	1.66666667	2.50544662	2.2875817	2.17864924	1.4973262
contig_1503	1.62361624	2.50481696	2.35988201	1.839303	2.26666667
contig_1505	3.45285525	6.28415301	4.71976401	3.41880342	2.31884058
contig_1506	2.66159696	1.3064133	2.60972716	1.18623962	0.60532688
contig_1507	2.26876091	3.4904014	2.96684119	2.96684119	2.09424084
contig_1509	1.65016502	2.63543192	2.14067278	2.60366442	1.16144019
contig_1510	0.81855389	2.00601805	2.03685742	0.72639225	1.179941
contig_1511	0.54024851	1.49812734	1.87265918	1.44539615	0.91397849
contig_1513	1.57367668	1.52598951	1.52380952	1.18539592	0.87463557
contig_1514	1.85185185	2.18855219	2.86195286	2.35690236	2.02020202
contig_1515	2.40673887	2.45901639	2.32240437	0.85470085	1.22100122
contig_1518	2.29591837	3.66161616	2.90771176	0.63694268	1.26262626
contig_1519	3.41463415	2.84757119	1.62601626	0.78308536	3.5440613
contig_151	0.7497657	2.51471375	2.1515435	2.83636364	1.08892922
contig_1520	0.73099415	1.21832359	1.26705653	0.34229829	1.31578947
contig_1521	8.21018062	6.40394089	6.5681445	6.41447368	6.07553366
contig_1525	1.81611805	2.60770975	2.46516613	2.40437158	1.93401593
contig_1526	1.12233446	2.91806958	3.47923681	2.24719101	2.58136925
contig_1527	1.66191833	2.41820768	2.72536688	1.05485232	1.87409899
contig_152	1.5503876	1.56054931	1.8974566	1.55393053	0.82417582
contig_1530	2.01342282	3.47003155	3.15457413	1.67189133	2.21052632
contig_1531	1.32890365	2.77777778	2.33333333	2.5462963	0.80091533
contig_1532	0.97465887	3.41023069	2.52469813	1.78571429	0.43431053
contig_1533	1.22265122	3.42598578	2.5128866	1.41935484	4.94699647
contig_1534	2.01196302	1.20177103	1.06778087	1.4893617	1.48947098
contig_1535	0.98434004	2.37136465	1.5212528	0.40268456	1.29753915
contig_1536	2.25225225	2.85285285	2.1021021	1.5	1.35802469
contig_1537	3.48923534	2.55474453	1.88370188	1.46788991	2.19123506
contig_1538	2.76564774	3.49344978	3.34788937	2.76564774	2.76564774
contig_1539	1.5742642	1.50581793	1.43737166	0.75290897	0.6844627
contig_153	1.59867696	1.06680213	0.92783505	0.81727963	1.10880111
contig_1540	2.12569316	3.41151386	1.20336943	3.145917	0.3006012
contig_1546	1.31729668	1.64870006	1.61476355	0.85763293	0.9044658
contig_1548	1.11336032	0.99009901	2.17216412	1.83852918	1.91082803
contig_1549	7.91896869	8.05860806	7.55064457	5.893186	8.10313076
contig_154	1.15340254	1.16086235	1.55482815	2.38095238	1.64338537
contig_1552	2.12234707	2.15462611	2.3030303	0.62656642	1.16731518
contig_1553	6.33802817	6.25	7.17299578	4.50070323	5.49295775
contig_1555	6.76691729	6.88806888	7.08955224	5.91194969	6.28930818
contig_1556	2.22222222	2.35294118	2.11360634	2.09150327	2.09150327
contig_1558	2.3923445	2.71132376	3.66826156	2.8708134	1.59489633
contig_1562	2.08333333	4.09924488	5	2.45098039	2.12527964

contig_1563	0.70335126	2.02020202	0.77805078	1.18670886	0.57330057
contig_1566	1.02389078	1.02389078	1.82025028	1.97674419	0.89186176
contig_1567	4.39040865	3.48943985	3.9974819	2.15866163	4.04040404
contig_1568	1.69491525	2.33333333	2.06489676	2.76816609	1.93401593
contig_1570	0.69637883	1.11420613	1.71773445	0.78922934	0.69637883
contig_1571	0.96818811	2.63157895	3.18118949	1.12517581	1.65975104
contig_1572	1.07279693	1.6091954	1.37931034	1.67810831	0.88300221
contig_1573	1.29990715	2.47678019	2.22929936	2.19435737	1.34158927
contig_1578	2.07522698	3.63164721	5.42635659	1.81582361	1.43229167
contig_1579	0.81806283	1.92173305	1.2464046	1.19047619	0.97914006
contig_157	0.82346886	1.41601563	1.59651669	0.68906115	0.76982294
contig_1580	1.50927124	1.30378096	1.61353798	1.98979592	2.77529096
contig_1581	1.05212817	1.53256705	1.48325359	0.90909091	0.47415837
contig_1584	0.89786756	1.40388769	4.30939227	1.13378685	3.41880342
contig_1585	2.72108844	3.15315315	3.85487528	2.49433107	2.49433107
contig_1586	1.85185185	3.08641975	2.4691358	1.54320988	2.00617284
contig_1587	2.11373931	3.25255102	1.52801358	1.2745098	2.18778487
contig_1589	0.53361793	3.85542169	6.23188406	5.21276596	0.96269555
contig_158	0.69686411	1.5503876	1.74418605	0	3.50690755
contig_1590	1.31086142	2.124834	4.3699187	1.12130479	1.39082058
contig_1591	1.6995614	2.48033878	0.98738343	1.36147039	1.73041894
contig_1592	1.70940171	3.51405622	3.28014184	2.248394	1.7565872
contig_1597	1.30853994	1.64899882	1.70187793	1.11567821	1.39616056
contig_1598	2.02020202	3.87205387	3.7037037	3.7037037	1.51515152
contig_1599	0.75839653	2.77777778	2.64741276	1.67973124	0.94043887
contig_15	2.89968652	2.63157895	1.59883721	2.19966159	3.28947368
contig_1600	1.63069544	2.93843284	1.49812734	1.85676393	1.37931034
contig_1602	2.54237288	3.24858757	2.54596888	3.10734463	3.10734463
contig_1603	1.90023753	2.14285714	2.84697509	2.41545894	2.03106332
contig_1606	0.86805556	2.54372019	1.68195719	1.04477612	0.68728522
contig_1608	2.00421941	1.29032258	0.94936709	0.8501594	0.95238095
contig_1610	2.24867725	2.51322751	2.77777778	1.85185185	1.98412698
contig_1611	1.40056022	1.28755365	1.1827957	1.79018976	2.06140351
contig_1612	1.70603675	1.96850394	1.57480315	0.78740157	1.44356955
contig_1616	2.67857143	3.125	2.97619048	2.67857143	2.67857143
contig_1619	1.38380138	1.46699267	1.77612557	1.50958792	0.73710074
contig_1620	2.11442786	2.73631841	3.10945274	1.61691542	2.48756219
contig_1621	2.47578041	4.19354839	4.28724544	2.15285253	1.73347779
contig_1623	1.08843537	1.51761518	1.33149679	1.14626318	0.73159579
contig_1624	1.76245211	2.48397436	1.99826238	0.94562648	1.29171152
contig_1627	1.17252931	1.50753769	1.6722408	0.5027933	0.78168621
contig_1628	1.16959064	2.47349823	1.87416332	1.80995475	1.4541387
contig_162	0.87082729	2.13017751	2.20264317	1.04166667	1.25
contig_1630	0.58522312	1.64644714	1.51111111	0.81135903	1.58286778
contig_1631	1.86403509	2.38095238	1.70250896	1.72413793	1.19176598
contig_1634	5.86419753	2.77777778	3.54938272	5.09259259	2.77777778
contig_1636	0.89285714	6.55509066	3.66197183	2.8831563	1.53609831
contig_1639	6.70955882	5.58069382	6.34920635	5.59352063	5.35444947
contig_163	0.86542622	2.38095238	2.66259275	1.82106097	1.2295082

contig_1640	1.09041345	0.7269423	0.99954566	0.54545455	0.90991811
contig_1643	2.14629873	1.96592398	2.05061082	0.56719023	1.30947185
contig_1644	0.80738178	1.86615187	1.2455516	1.06761566	0.71174377
contig_1645	0.98176718	2.57142857	4.36681223	1.10344828	1.99146515
contig_1647	3.60824742	1.41242938	3.22580645	4.66472303	1.37221269
contig_1649	1.12847222	1.73611111	2.03993056	0.65104167	0.56423611
contig_1650	1.40721196	2.18579235	1.63934426	1.36612022	1.0936432
contig_1652	1.42118863	2.22634508	2.55905512	0.4109589	1.56971376
contig_1653	1.81043663	3.01075269	2.68240343	3.5483871	1.6286645
contig_1654	4.79452055	3.65296804	3.19634703	2.72108844	3.18906606
contig_1656	1.49253731	2.42424242	3.75757576	2.66666667	2.06060606
contig_1657	1.34228188	1.87018702	2.37288136	1.43964563	1.43014301
contig_1658	1.21816168	3.16301703	1.58150852	2.28070175	1.03092784
contig_1659	1.23809524	2.27703985	2.268431	1.79924242	1.44329897
contig_1662	1.2202208	1.66954519	1.84225676	1.15141048	0.86355786
contig_1664	1.88261351	4.5751634	3.26797386	3.16248637	1.66297118
contig_1665	2.06349206	3.22164948	2.22222222	2.3923445	1.10497238
contig_1666	1.66453265	4.38842204	2.95040804	1.89701897	1.48148148
contig_1667	1.02040816	1.58353127	1.80115274	1.26475548	2.50260688
contig_1668	1.55279503	1.96078431	3.93313668	1.37566138	0.94339623
contig_1669	1.93370166	3.19042871	3.5480859	2.72045028	3.41207349
contig_166	1.3867877	1.35658915	1.22282609	1.09990834	1.44927536
contig_1670	1.53846154	2.05128205	1.54958678	2.45614035	2.25641026
contig_1671	1.21212121	1.06060606	2.12121212	1.06060606	1.81818182
contig_1673	11.2418301	12.0622568	11.9164619	11.4583333	10.3538663
contig_1674	1.20761728	1.20761728	1.2987013	1.06679035	1.61290323
contig_1675	0.53821313	2.04301075	3.11827957	2.68817204	1.13895216
contig_1676	0.93046033	3.08716707	1.40485313	1.15979381	1.49118843
contig_167	3.41880342	2.56410256	4.27807487	2.73631841	1.40280561
contig_1681	0.4408274	1.3903018	0.4408274	0.3051882	0.6442862
contig_1684	0.71343639	3.25644505	3.99061033	2.68907563	1.62094763
contig_1685	2.24	1.16861436	2.64026403	1.73775671	1.26382306
contig_1686	0.94810379	1.49700599	0.89820359	0.94857713	0.5988024
contig_1688	2.71444083	4.08970976	3.31325301	0.88582677	2.95566502
contig_168	1.65816327	1.40421264	1.34874759	2.59179266	1.59883721
contig_1690	3.13315927	6.27306273	4.61165049	1.54639175	1.18918919
contig_1693	2.61437908	3.26797386	2.61437908	3.05010893	2.81385281
contig_1694	1.73796791	2.50329381	2.76890309	0.98400984	2.05580029
contig_1695	1.49516271	3.56200528	4.79331574	2.00421941	3.52267723
contig_1696	1.85185185	3.27272727	3.50877193	3.14465409	2.4822695
contig_1697	1.49418926	1.49336283	1.27212389	0.6833713	2.23921354
contig_1698	1.49812734	3.17124736	2.92792793	2.85714286	1.91339376
contig_1699	1.66919575	2.0033389	2.11382114	5.26315789	2.19619327
contig_169	0.84295973	0.83955224	0.83798883	1.33928571	1.4084507
contig_16	0.79066265	1.19378123	0.87570621	2.44865719	1.0393467
contig_1701	1.92090395	2.31884058	2.11538462	0.73529412	1.41843972
contig_1704	2.5297619	2.67857143	2.45310245	1.78571429	1.58045977
contig_1705	1.6	2	2	1.27226463	0.64020487
contig_1709	1.07936508	2.09003215	1.90114068	1.65079365	0.82382763

contig_170	0.68870523	1.76170607	0.8302583	1.34032634	1.30260521
contig_1710	1.31578947	4.64071856	5.14705882	1.04166667	0.79744817
contig_1712	1.12721417	2.22222222	2.38095238	2.6984127	0.79491256
contig_1713	1.15830116	0.92272203	2.20883534	1.20218579	0.7360673
contig_1717	6.99404762	8.49358974	8.65384615	7.69230769	7.21153846
contig_1718	1.04166667	1.65441176	1.71568627	1.05337079	0.73529412
contig_171	2.26244344	1.01010101	0.65288357	0.74906367	0.60851927
contig_1724	0.88888889	1.69082126	1.33547009	1.97238659	1.82872435
contig_1726	0.88626292	1.42786805	1.62481536	1.87192118	1.33070478
contig_1727	0.80183276	1.56599553	1.34228188	1.11856823	1.36612022
contig_1729	1.85922975	3.32056194	3.61445783	2.43902439	1.63043478
contig_172	1.11223458	1.3164557	1.46022155	0.59055118	0.90909091
contig_1734	3.23383085	3.48258706	3.23383085	2.23880597	2.48756219
contig_1735	0.68250068	1.63800164	1.42270862	1.37136588	0.43907794
contig_1737	1.21518987	1.58286778	0.87962963	1.90343547	0.79069767
contig_1738	2.52764613	3.15955766	3.15955766	1.73775671	2.21169036
contig_1739	0.33585223	2.42865817	2.7972028	1.95845697	2.1978022
contig_1741	1.57480315	2.2587269	3.24074074	1.28205128	2.1978022
contig_1742	2.66040689	3.85208012	2.85171103	2.65210608	1.29310345
contig_1744	2.0698577	2.9972752	3.62622036	2.00501253	1.7369727
contig_1745	2.53025303	2.96127563	2.18068536	1.59235669	1.29411765
contig_1746	4.07358739	3.63164721	3.64583333	3.73230373	1.03761349
contig_1747	2.53583241	3.79965458	3.41034103	3.16939891	2.57575758
contig_1750	2.36220472	4.82866044	5.60747664	3.27102804	2.80373832
contig_1751	3.58255452	2.42424242	0.20491803	1.01419878	0.21459227
contig_1752	0.53390283	2.2697512	1.76	0.93333333	0.85447263
contig_1753	1.82849937	2.06938527	2.10784314	0.93283582	2.83261803
contig_1754	2.60770975	2.13963964	2.70833333	2.64026403	1.42076503
contig_1755	0.97259063	1.82328191	2.61072261	1.89393939	1.21552127
contig_1757	1.43487859	2.54706534	3.22927879	3.34448161	2.75633958
contig_1758	0.80996885	1.12149533	1.05985037	1.24688279	0.74812968
contig_1759	4.17310665	3.125	4.60829493	6.69642857	3.12989045
contig_1760	1.08077361	1.70357751	1.6467916	2.61215219	1.76036343
contig_1761	0.91097308	1.57539385	1.2964348	1.47952444	0.93457944
contig_1762	0.73924731	1.34453782	0.70598007	2.62096774	1.71232877
contig_1763	1.70682731	3.09597523	1.82370821	2.0768432	1.46750524
contig_1764	1.82841069	2.86754665	1.36986301	1.96706313	3.51919561
contig_1766	8.41865757	9.58751394	11.0344828	7	8
contig_176	1.78861789	5.59862188	2.1150033	0.65789474	0.55865922
contig_1770	2.30680507	1.56424581	2.18390805	0.72115385	0.73260073
contig_1771	1.67958656	2.90456432	3.09139785	1.25313283	1.82291667
contig_1772	3.58974359	2.61538462	3.21100917	0.91883614	2.48833593
contig_1774	1.12647209	2.86144578	2.47311828	0.62926062	1.28617363
contig_1775	1.01010101	1.71277997	2.4218406	1.55141844	1.22645642
contig_1776	2.16284987	2.86783042	3.56703567	3.99500624	1.35802469
contig_1777	1.31687243	1.56378601	2.05761317	1.72839506	1.2345679
contig_1778	0.67114094	1.11856823	1.34228188	0.950783	0.44742729
contig_177	1.33067199	1.23626374	2.02808112	1.40721196	0.52910053
contig_1782	1.94552529	2.20779221	2.85343709	1.94552529	0.65876153

contig_1784	2.00945626	3.5971223	3.0075188	3.89294404	0.94979647
contig_1786	2.42214533	2.58175559	3.10880829	1.55440415	1.89003436
contig_1788	11.4543115	10.4247104	11.3256113	12.2265122	10.6821107
contig_1789	2.14592275	2.5751073	2.5751073	2.00286123	2.00286123
contig_178	2.42424242	1.85854414	1.30120482	1.52046784	2.79329609
contig_1790	2.13963964	2.47747748	4.97925311	2.6560425	2.68456376
contig_1791	1.1820331	2.1599533	1.87134503	2.1675454	1.32871173
contig_1794	2.00501253	2.89855072	2.00501253	1.87969925	2.88582183
contig_1795	0.29535865	2.86919831	3.23108384	1.76344086	2.16396171
contig_1796	1.45985401	3.06242638	3.29024677	0.57012543	2.15827338
contig_1798	2.04301075	5.05529226	2.97619048	2.91715286	2.143951
contig_179	1.24190065	1.23855681	1.67206041	1.05263158	2.47933884
contig_1800	1.63265306	3.1292517	2.85714286	1.76870748	2.04081633
contig_1801	3.125	2.93785311	3.02013423	2.72108844	2.93453725
contig_1803	1.72143975	3.52303523	5.21390374	2.59562842	2.21238938
contig_1805	1.24378109	2.77929155	1.75019889	1.49551346	1.54228856
contig_1807	1.09649123	1.91885965	2.07991242	0.4950495	1.53508772
contig_1808	1.77121771	2.72536688	3.08430432	2.23274696	0.49751244
contig_180	1.6091954	2.19478738	2.10045662	1.85185185	1.13960114
contig_1810	2.18790219	2.83140283	2.44530245	1.58150852	0.78037904
contig_1812	1.51921358	2.14477212	2.41286863	4.16666667	5.72864322
contig_1813	3.66161616	4.15549598	3.16205534	3.16205534	1.66666667
contig_1814	1.27226463	1.44189992	1.27226463	0.84961767	1.18443316
contig_1815	3.26086957	3.17460317	4.9200492	7.51633987	3.74269006
contig_1817	10.6306306	10.6306306	11.1913357	5.9566787	0.39761431
contig_181	0.25510204	4.30839002	1.05900151	1.11940299	1.33657351
contig_1821	1.91713049	1.5234613	2.0795107	0.61012813	0.97859327
contig_1822	2.85714286	3.52480418	2.51572327	1.78117048	1.7287234
contig_1824	1.61111111	1.68350168	1.89701897	1.54781647	2.20022002
contig_1825	1.11111111	1.38427464	1.21816168	0.71982281	0.94130676
contig_1826	2.50284414	2.08092486	1.07874865	1.91532258	2.2246941
contig_1827	0.68259386	1.55685293	0.60367454	1.39481924	0.50147493
contig_1830	3.58306189	2.47116969	2.68389662	1.41043724	0.26041667
contig_1834	2.27272727	4.10958904	3.18181818	1.82648402	1.82648402
contig_1838	2.82485876	1.3029316	0.99667774	2.93333333	0.30769231
contig_183	1.92893401	1.67714885	2.18045113	0.73746313	0.66666667
contig_1840	4.40985733	4.88771466	2.91508238	0.9009009	2.30179028
contig_1841	0.99502488	1.43949618	2.17969165	1.31004367	0.40834846
contig_1843	0.88587806	2.34848485	1.41414141	1.17948718	1.50884495
contig_184	0.84286574	1.38637734	1.27041742	1.26735063	3.9408867
contig_1851	1.15830116	1.99871051	1.86615187	1.02761721	1.99485199
contig_1852	0.9009009	4.13793103	2.51968504	2.92598967	2.9535865
contig_1855	0.71599045	1.16550117	1.26582278	1.23762376	0.47961631
contig_1857	2.15053763	2.4122807	2.30263158	1.32450331	0.96982759
contig_1860	11.4583333	11.4583333	10.9375	11.023622	9.89583333
contig_1864	1.98482195	2.89855072	1.9517206	0.86823289	1.61127895
contig_1865	3.69685767	3.81526104	4.81927711	1.3536379	2.81124498
contig_1867	3.04878049	3.93258427	3.05810398	3.58306189	1.3916501
contig_1869	2.21518987	3.07328605	3.51190476	0.6501182	2.00945626

contig_1870	2.52873563	3.13901345	4	1.36363636	2.75862069
contig_1871	1.06951872	1.63599182	1.8442623	4.16666667	1.64609053
contig_1876	1.99475066	1.52471083	1.31302521	1.62729659	0.73490814
contig_1878	0.80098583	1.41712877	2.03327172	0.80098583	1.29390018
contig_1879	0.78513478	1.64775797	1.38308977	1.41309468	1.56599553
contig_187	1.29032258	3.77867746	4.95867769	2.10409745	9.64360587
contig_1880	1.40291807	2.94117647	1.38197899	1.48749155	1.0567297
contig_1882	6.18357488	7.41324921	7.50988142	5.26315789	3.64197531
contig_1883	1.20481928	2.06666667	2.07914152	1.53333333	1.16175156
contig_1884	2.93333333	4.26666667	3.46666667	3.46666667	2.4
contig_1885	1.558753	1.34297521	1.79856115	0.47961631	1.44752714
contig_1886	0.42060988	1.24333925	3.6900369	1.15062762	0.91743119
contig_1887	2.60162602	2.87262873	2.76422764	1.76304654	1.95758564
contig_1888	0.808369	1.35746606	1.1778563	1.12830432	1.16054159
contig_188	2.88836846	3.30396476	2.0383693	2.01072386	1.99556541
contig_1891	1.01010101	2.08596713	2.3989899	1.70454545	0.88383838
contig_1893	1.38010351	1.64609053	3.14318976	1.44032922	2.10759845
contig_1894	2.08197788	2.90360046	2.03412073	0.63069376	0.79681275
contig_1896	0.94810379	1.33862172	1.19760479	1.19760479	1.21308017
contig_1897	1.94931774	4.73251029	4.54545455	2.6	3.30969267
contig_1898	1.97238659	1.81818182	3.03030303	1.38888889	2.22222222
contig_189	1.16391853		2.12765957	0	0.25
contig_1900	2.37416904	2.03883495	2.29540918	2.77246654	1.24610592
contig_1901	3.24254215	2.73631841	3.39622642	4.6854083	3.3492823
contig_1903	0.47619048	2.1978022	2.00803213	0.78431373	2.03442879
contig_1905	1.65333333	1.58013544	1.07163001	1.06801574	1.18577075
contig_1906	0.55555556	1.62259615	2.15827338	1.67865707	1.08108108
contig_1908	4.25531915	5.92105263	5.08474576	3.07692308	4.52380952
contig_190	0.42849491	1.49333333	0.80472103	0.63041765	3.30033003
contig_1910	2.17755444	3.44827586	3.03030303	0.83752094	1.52284264
contig_1913	1.59045726	1.52180275	1.04071013	0.97777778	0.77014218
contig_1915	0.66603235	1.05919003	1.37844612	1.4953271	0.93283582
contig_1917	1.05919003	3.14285714	6.07843137	0.80160321	2.31092437
contig_1918	6.12648221	2.51937984	3.87596899	2.85714286	2.88461538
contig_1919	0.81081081	4.3715847	3.82513661	3.27868852	3.55191257
contig_1925	4.3715847	0.93676815	3.2	2.14477212	0.99009901
contig_1929	1.71919771	2.0361991	1.96078431	0.56270096	1.05580694
contig_192	1.2066365	2.35988201	1.39968896	3.0758226	1.11292074
contig_1931	3.64583333	2.75689223	2.65700483	1.99004975	1.62037037
contig_1933	2.02020202	2.95138889	3.09278351	1.69491525	1.48619958
contig_1934	1.64319249	1.76574456	2.6035503	0.46701693	1.39372822
contig_1935	1.24087591	1.96936543	1.75054705	1.16703136	1.02189781
contig_1937	5.2545156	5.51724138	4.95049505	4.79233227	3.2183908
contig_1938	2.62593783	3.44234079	3.58974359	2.68817204	2.29885057
contig_1940	0.87090164	7.85100287	1.31729668	1.09427609	2.74914089
contig_1941	0.98610489	1.34770889	1.15197164	0.64143682	1.10935024
contig_1942	8.48484848	10.9090909	9.09090909	9.39393939	6.93333333
contig_1943	3.33333333	4.52380952	4.76190476	3.35731415	3.83693046
contig_1944	1.61090458	2.91201983	1.67286245	0.74349442	0.86741016

contig_1947	0.93998554	2.10144928	1.8076645	1.52505447	1.37681159
contig_1948	1.2345679	1.33951571	1.70015456	1.48641722	0.92165899
contig_1950	3.26340326	2.72835113	2.71867612	3.01568154	2.13464696
contig_1953	0.95408468	1.2173913	2.44047619	2.49287749	0.93048714
contig_1955	1.72413793	0.72463768	2.7027027	2.36486486	4.37710438
contig_1957	2.04081633	2.39651416	4.1322314	5.17529215	2.2675737
contig_1958	2.45398773	3.88548057	4.90797546	3.27198364	2.24948875
contig_1959	1.69121658	1.68569875	1.8579235	0.81967213	1.25272331
contig_1960	0.10976948	1.447178	1.7017017	1.16550117	1.98019802
contig_1962	0.79051383	1.01066816	1.22767857	0.97926267	1.90023753
contig_1966	1.28617363	2.51471375	2.53642742	2.62664165	1.78777393
contig_1968	1.34770889	1.07825015	0.92421442	0.67775724	0.46210721
contig_196	0.64239829	5.7106599	2.70935961	0.90702948	2.94840295
contig_1970	0.76023392	0.70546737	1.11111111	1.40350877	0.64327485
contig_1971	5.01474926	7.96460177	5.98290598	3.57142857	4.62046205
contig_1972	1.36645963	1.24069479	1.1993383	1.28205128	0.68551842
contig_1973	0.88008801	1.96850394	1.04510451	0.99009901	0.440044
contig_1977	2.00573066	5.14446794	2.26628895	4.89296636	1.57142857
contig_1978	1.90664037	1.86046512	0.89514066	0.71754729	0.86642599
contig_1979	1.07674685	1.17220801	1.23641015	0.71761751	0.7904294
contig_1982	2.5210084	3.07955518	2.14830215	2.42550243	2.77200277
contig_1983	1.12847222	3.08641975	2.57092199	0.97001764	2.11081794
contig_1984	0.9042954	1.88111362	1.58013544	1.05421687	0.82768999
contig_1985	0.86455331	2.72401434	1.43472023	1.17130307	1.93548387
contig_1987	0.85574572	1.38549307	1.26324368	0.24489796	0.48899756
contig_1988	0.83160083	1.16358658	1.02669405	1.4803849	0.58181818
contig_1989	1.15226337	3.21057602	2.72277228	1.57676349	1.81668043
contig_1990	1.03199174	1.34297521	1.44478844	0.92879257	1.24223602
contig_1992	1.70940171	1.70697013	2.2840828	1.51515152	1.86781609
contig_1993	0.95057034	1.20405577	0.76045627	1.33079848	0.62073246
contig_1994	1.48448043	2.4291498	2.15924426	0.74274139	1.558753
contig_1996	2.25464191	1.80055402	2.08913649	2.95608108	1.20554551
contig_1997	1.36138614	1.47420147	1.4732965	0.7985258	1.12508273
contig_1998	1.44822592	2.88461538	4.18470418	2.56222548	1.45032632
contig_19	0.92592593	1.66773573	1.11111111	2.75689223	1.1627907
contig_1	2.69749518	1.39211137	4.01606426	1.26874279	2.34234234
contig_2002	1.38248848	1.95635816	1.73062453	1.4461316	2.63355907
contig_2004	0.77186964	1.17647059	1.19521912	0.46082949	0.85413929
contig_2005	1.26213592	1.52013147	1.71102662	0.51113545	1.07780397
contig_2007	1.11642743	1.33020344	1.27591707	1.50078989	0.87025316
contig_2008	1.45985401	2.3862789	1.86567164	0.59656972	1.81132075
contig_2009	0.6752609	1.96439533	1.99430199	0.78226858	0.7029877
contig_2010	1.6139879	2.97339593	3.99385561	1.98776758	2.62430939
contig_2011	0.88726514	0.8045977	1.11016225	1.16754232	0.49833887
contig_2016	3.69843528	2.44077531	3.07692308	2.82051282	4.26951301
contig_2017	0.96510765	1.52529762	2.66955267	1.11832612	0.86580087
contig_2018	2.08717004	0.89801155	1.18824265	1.96439533	0.53727334
contig_2019	0.76717812	1.89054726	1.12769486	1.71624714	3.07167235
contig_201	0.34423408	2.11193242	0.96582467	1.04050539	0.62597809

contig_2020	0.58160669	1.34643377	1.23591421	1.19956379	0.43620502
contig_2021	1.49156939	2.20492866	2.14007782	0.58479532	1.04370515
contig_2022	5.71428571	6.59472422	6.47482014	6.07476636	5.69544365
contig_2023	9.76800977	9.56459672	7.88026878	8.92966361	9.34065934
contig_2024	0.44960659	1.34831461	1.64794007	1.73464604	0.89921319
contig_2027	1.76730486	2.52525253	2.06185567	3.67917586	1.17733628
contig_2028	1.10356537	1.81945416	2.24246671	2.55524862	0.93209055
contig_2029	1.43369176	2.8551035	1.65672631	2.29390681	2.41648898
contig_202	0.63411541	1.2482663	1.34978789	3.27552987	1.15651503
contig_2030	2.23367698	2.57731959	3.43642612	2.57731959	2.23367698
contig_2032	0.41797283	0.87077673	1.01010101	0.59212818	0.34831069
contig_2034	1.00574713	1.58045977	1.57367668	1.70454545	0.9352518
contig_2039	0.44368601	0.85470085	1.03276353	1.1208577	0.56980057
contig_203	0.91743119	0.95619988	0.88495575	0.92503987	1.35869565
contig_2042	0.37950664	2.28571429	1.80012415	0.62383032	0.70108349
contig_2044	1.25786164	1.52740341	1.97663971	1.25786164	1.52740341
contig_2046	1.04399702	1.41685309	1.67158309	0.59701493	0.89485459
contig_2048	0.90439276	0.98506514	2.01020102	0.96649485	0.45931759
contig_204	0.28901734	1.76678445	1.60116448	0.41958042	1.8705036
contig_2050	1.02564103	1.53977759	1.45299145	0.60085837	1.11111111
contig_2051	0.92776673	2.51762336	1.65672631	1.06312292	0.53262317
contig_2052	1.67264038	1.90217391	1.35869565	2.10325048	0.81521739
contig_2053	0.81148564	0.94280327	1.05919003	1.24843945	2.04081633
contig_2054	0.79710145	1.04868914	0.80704329	0.45078888	2.41874528
contig_2055	2.83333333	2.58333333	1.83333333	1.67084378	1.98840099
contig_2057	1.7626322	3.83693046	4.27764326	3.50877193	1.68134508
contig_2058	1.86721992	1.95023537	2.97372061	2.47933884	0.64102564
contig_2059	1.42671855	2.14007782	2.13592233	0.64850843	1.55339806
contig_2060	2.47524752	3.3744856	3.3744856	2.38683128	1.31687243
contig_2061	1.55925156	2.27038184	2.57997936	0.92975207	1.13519092
contig_2062	2.60303688	3.00653595	2.4911032	0.25220681	1.27877238
contig_2066	1.3592233	2.26977951	1.76470588	1.30975769	0.96818811
contig_2067	0.86887836	1.18483412	1.8957346	0.9478673	0.55688146
contig_2068	1.45631068	1.937046	1.85633575	0.8071025	1.04923325
contig_2070	1.72413793	3.33148251	1.70940171	1.97628458	1.25223614
contig_2071	1.91304348	2.18855219	2.53093363	2.13603148	0.90702948
contig_2074	1.74966353	2.14067278	2.15875125	1.4940239	1.12919296
contig_2075	1.17878193	2.74509804	2.29207597	0.91683039	1.24426981
contig_2076	1.68421053	1.89473684	1.68421053	0.49586777	0.9122807
contig_2078	0.77765995	1.78571429	1.10650069	1.16731518	0.80246914
contig_2079	1.62297129	2.62172285	2.43445693	2.30961298	2.20922677
contig_207	1.01651842	0.71428571	2.43243243	1.79738562	0.19120459
contig_2080	1.9379845	1.62790698	2.79069767	1.62790698	1.1627907
contig_2081	1.49911817	3.43709468	2.53968254	1.41604855	1.14942529
contig_2083	0.99180681	1.76800345	1.86413902	1.20741699	0.64683053
contig_2085	1.05401845	2.77777778	3.31023865	1.02179837	0.53440214
contig_2087	2.15053763	1.51994934	1.39152435	0.69576218	1.64452878
contig_2088	1.15361263	0.667881	1.76077717	0.78787879	1.37976347
contig_2089	1.66172107	1.41538462	1.96439533	0.35335689	1.04551046

contig_2092	7.26392252	7.60517799	9.14979757	8.11030008	7.95454545
contig_2094	1.86741363	1.49371069	3.90625	1.41451415	2.69230769
contig_2096	0.65127294	1.28283445	1.03092784	1.21432908	1.11940299
contig_2097	1.00738751	2.35215054	1.41414141	1.88552189	1.14017438
contig_2098	1.7515052	2.04319907	3.16939891	0.54377379	1.28205128
contig_2099	1.49739583	2.41874528	3.05989583	0.95177665	0.9334163
contig_209	1.06707317	2.90187094	2.7640264	1.67130919	0.99304866
contig_2101	1.25084517	1.3229308	1.56143924	1.04942451	0.98438561
contig_2102	0.88028169	1.12759644	2.07253886	1.29339687	1.14526823
contig_2105	1.58988993	1.91524042	1.34474328	0.73409462	1.75224124
contig_2106	1.96463654	2.5974026	1.94805195	1.26582278	1.41342756
contig_2107	2.81582953	3.45313601	3.42902711	0.9569378	2.28613569
contig_2108	1.71277997	2.32240437	2.72783633	1.40562249	1.41975309
contig_2109	1.31021195	1.93124759	1.32275132	1.19783617	0.96562379
contig_2111	6.84168656	6.92124105	6.84168656	6.27943485	7.08035004
contig_2114	1.4200299	1.49476831	2.16741405	0.41928721	0.89753179
contig_2115	0.86898396	1.74029451	1.80722892	2.54350736	1.40562249
contig_2116	2.67794221	3.68794326	4.46428571	2.12765957	2.47699929
contig_2117	1.38549307	1.71149144	1.22249389	1.46699267	1.0594947
contig_2118	0.79051383	2.10803689	1.20391272	1.73062453	0.60240964
contig_2119	1.57303371	2.68276325	1.42322097	1.4200299	1.19850187
contig_2120	1.53640615	1.88172043	1.88172043	1.27688172	0.80699395
contig_2121	2.38095238	3.54713314	1.78147268	3.12093628	1.03177879
contig_2122	1.51802657	1.58127767	1.32827324	0.69576218	1.07526882
contig_2123	2.49084249	2.12609971	3.003663	0.73260073	1.61172161
contig_2124	0.74880871	1.36383634	1.93482688	1.7311609	2.37852531
contig_2125	3.79789759	3.66225839	3.42488979	2.79386712	2.43822076
contig_2126	2.11678832	1.97512802	1.4587892	0.97891566	1.97224251
contig_2128	0.93795094	2.52525253	2.74170274	1.22655123	1.43988481
contig_2129	1.70749814	3.22580645	2.76461295	2.26773958	1.39808683
contig_2131	1.31396957	3.28820116	1.33490381	2.58865248	2.85877644
contig_2132	0.65466448	2.03748981	2.77098615	1.54506438	3.2599837
contig_2133	1.73410405	1.91204589	1.52187698	2.45795602	2.01691607
contig_2135	1.13333333	2.7197976	2.14646465	1.74081238	0.93333333
contig_2137	1.73824131	2.54295533	3.02114804	2.12183436	2.53251198
contig_2138	0.71243523	3.42555995	3.36569579	3.30634278	1.35409614
**contig_213**	**0.98684211**	**2.26824458**	**5.64417178**	**3.53982301**	**0.88105727**
contig_2140	1.28068303	0.84507042	1.30904952	2.28640193	0.60827251
contig_2141	1.49739583	1.84818482	1.63719712	0.65104167	0.86092715
contig_2143	0.76045627	2.29645094	2.44926522	1.91625266	0.90972708
contig_2144	1.32325142	2.58724428	2.95597484	2.07677785	1.94057004
contig_2145	1.54569892	1.60337553	1.94892473	0.94149294	0.88075881
contig_2146	0.40803515	0.94073377	1.75768989	1.03626943	1.10062893
contig_2147	1.2608353	1.10323089	1.41843972	1.33963751	0.94562648
contig_2148	1.05177994	1.61812298	1.69902913	0.80906149	1.77993528
contig_214	1.83566434	7.74647887	3.71517028	1.32231405	3.42935528
contig_2150	0.6782535	2.44238046	1.20439249	0.95744681	1.41542817
contig_2151	2.3916293	3.73692078	2.60999254	1.34529148	2.31689088
contig_2152	1.31944444	3.75521558	1.25	2.1515435	0.85139319

contig_2153	0.71377587	1.36239782	1.58227848	1.29824561	1.27163546
contig_2156	0.40968343	0.9310987	1.52700186	1.15456238	0.40968343
contig_2157	1.1042098	2.36991242	3.21637427	2.20022002	1.83867141
contig_2159	0.65549891	2.24179343	1.53172867	0.51207023	1.72413793
contig_215	0.84488003	2.48976808	1.35180804	1.22866894	1.16807268
contig_2161	2.30341541	1.22914838	1.42180095	0.84985836	1.63120567
contig_2162	2.6293469	3.23679727	2.61603376	2.4765158	1.03011094
contig_2163	1.04821803	1.22164049	1.98290598	0.31446541	0.61287028
contig_2164	0.93457944	1.51515152	1.33819951	1.88087774	1.20361083
contig_2166	0.8984726	1.71379606	3.04259635	1.30963517	0.71633238
contig_2168	1.62774239	1.7033357	1.90895742	0.5904059	1.98237885
contig_2169	1.1875	2.39197531	1.93508115	0.62774639	2.14438885
contig_216	1.91296031	2.6297086	2.40269101	1.95227766	1.46579805
contig_2170	0.40716612	2.11898941	0.86132644	0.89285714	1.94174757
contig_2172	1.60466813	1.52394775	2.64227642	1.85459941	0.88888889
contig_2173	1.92837466	2.20994475	1.35501355	0.62724014	1.10803324
contig_2174	3.51758794	3.1124498	2.51762336	2.48190279	1.6064257
contig_2177	2.72822665	3.06122449	3.07692308	1.59128978	1.75
contig_2178	3.66666667	2.27920228	1.70940171	1.69971671	2.37580994
contig_2179	1.62241888	3.24675325	1.94579569	1.87125749	1.04011887
contig_217	1.24434389	3.17460317	2.43084661	0.85324232	0.82417582
contig_2181	1.03473762	1.69992609	1.62601626	1.33037694	1.69992609
contig_2182	0.50621261	1.45939086	1.19958635	0.54291624	0.63979527
contig_2185	1.2962963	1.57407407	1.57407407	1.75925926	0.87463557
contig_2186	1.16959064	2.08877285	1.75879397	1.56765677	0.60922541
contig_2188	0.99821747	1.86813187	1.478318	1.33944307	0.90847914
contig_2189	1.07526882	2.88248337	1.73973556	1.86781609	1.30718954
contig_218	1.03626943	0.23640662	1.94552529	6.16045845	1.50334076
contig_2191	1.08447489	1.7704169	1.31278539	1.03448276	0.97198399
contig_2194	1.42768467	3.24005891	3.58333333	1.60295931	0.49875312
contig_2196	0.79666161	0.75843762	1.55715913	1.32726583	1.21442125
contig_2197	2.22222222	2.30381569	2.75862069	0.30721966	1.55228758
contig_2198	1.74672489	1.40117994	1.38312586	2.03340595	6.29067245
contig_2199	2.41433022	3.19314642	2.99625468	2.09953344	1.51860289
contig_219	2.28091236	1.97789412	2.15903107	2.00945626	2.61584454
contig_21	1.25313283	2.56776034	1.94552529	0.73891626	1.31147541
contig_2201	1.39433551	2.43372447	2.09698558	1.66011359	0.74268239
contig_2202	2.10674157	2.8830313	1.79186768	1.2482663	3.34261838
contig_2203	1.91826522	3.04487179	1.82270091	1.95911414	1.85185185
contig_2204	1.6	1.86666667	3.02222222	1.42222222	1.95555556
contig_2205	1.8907563	3.12722104	5.0297816	3.16856781	7.0324575
contig_2206	1.58730159	1.68650794	2.28174603	1.48809524	0.99206349
contig_2207	1.30890052	1.39616056	1.48342059	1.65794066	4.01606426
contig_2208	2.14477212	3.5971223	3.16573557	2.60707635	1.58286778
contig_2209	1.91570881	3.5339064	3.5440613	2.87356322	2.5862069
contig_220	2.17755444	1.61812298	1.51260504	0.63844086	0.64102564
contig_2210	4.16666667	4.31309904	2.54574383	4.11954766	6.06758833
contig_2213	0.98039216	1.84818482	0.98039216	1.2605042	0.86149768
contig_2214	1.86688312	2.92682927	2.97906602	1.20772947	1.90555095

contig_2215	1.72413793	2.86738351	2.29390681	1.20141343	0.85106383
contig_2216	2.52808989	2.3297491	2.58019526	1.40252454	1.32774284
contig_2217	1.05448155	1.82322632	1.47351653	1.1257954	0.75486691
contig_2219	1.34600158	2.21694378	2.05859066	0.79176564	1.10847189
contig_221	2.69413629	4.32098765	3.3201581	2.090301	1.40515222
contig_2220	1.86846039	2.23214286	1.81950509	2.54681648	0.9715994
contig_2221	0.84097859	1.0230179	0.81481481	1.70068027	1.2755102
contig_2223	1.24888492	1.78372352	1.35135135	0.62444246	0.92165899
contig_2224	0.60150376	1.55993432	1.82648402	0.86139389	0.77821012
contig_2225	6.51340996	6.05263158	6.42570281	6.36856369	5.98958333
contig_2226	1.80064309	1.62885398	1.56340475	1.48148148	1.68776371
contig_2227	1.71339564	2.72108844	2.81797411	2.10684725	1.02929533
contig_2228	0.50441362	1.61812298	1.34510298	0.42052145	1.50753769
contig_2229	2.44698206	3.43980344	3.67047308	1.76380368	1.47058824
contig_222	1.60520607	1.35048232	3.23551543	1.55163189	1.75824176
contig_2232	2.28359002	2.84842319	2.29434807	1.62647224	0.90566038
contig_2234	1.41342756	3.95968323	1.92982456	1.55355683	0.78947368
contig_2235	1.07526882	3.37643678	3.54609929	2.87356322	2.0480226
contig_2237	1.24429697	1.78041543	2.02375715	1.56862745	1.51515152
contig_2238	1.29672006	1.34281201	2.05627706	0.53394355	1.10584518
contig_2239	1.29224652	2.29083665	1.68650794	2.48262165	0.9009009
contig_2240	1.57480315	1.65794066	2.44328098	1.39982502	0.78534031
contig_2241	1.63304515	1.92122959	1.72910663	1.07108082	1.53698367
contig_2242	2.85087719	5.2154195	2.32383808	3.73765867	2.88248337
contig_2246	1.24777184	1.36904762	1.09689214	1.30952381	0.58377116
contig_2247	6.53303638	6.16184113	5.56792873	5.41945063	5.64216778
contig_2248	0.70028011	1.54061625	1.33333333	1.33614627	0.98245614
contig_2251	1.45576708	1.82904069	2.01567749	1.38111236	1.00783875
contig_2252	1.20336943	2.25080386	2.78670954	1.60771704	0.43290043
contig_2253	1.29339687	1.50170648	1.62711864	2.05303678	1.49659864
contig_2254	0.82135524	1.55367232	2.47823175	2.61299435	1.99704142
contig_2257	1.59883721	2.55863539	2.13219616	1.3503909	1.35617416
contig_2261	2.07852194	2.65755505	3.38722094	2.22734255	1.82232346
contig_2263	1.26849894	2.41411328	1.25786164	2.64072272	0.83682008
contig_2265	0.90497738	2.33009709	2.77777778	1.29533679	0.79522863
contig_2266	1.40944327	2.76595745	1.27659574	1.40944327	1.61744023
contig_2267	1.44927536	1.45228216	1.38312586	3.3957034	7.00416089
contig_2268	0.94086022	2.77777778	2.53333333	0.46666667	1.73333333
contig_226	1.96540881	1.53508772	3.10606061	1.20336943	1.20481928
contig_2270	1.22807018	1.43884892	1.34408602	1.74793008	1.17924528
contig_2271	1.09529025	1.65361184	2.64084507	0.34482759	1.13043478
contig_2272	2.21378874	2.40506329	1.70777989	2.5	1.26984127
contig_2273	1.93050193	2.44845361	2.44530245	2.57566001	1.88434048
contig_2274	1.91815857	3.14519604	3.59435173	1.3955985	1.63398693
contig_2275	1.50793651	2.06349206	1.66666667	1.11111111	0.87929656
contig_2279	1.27287192	2.62529833	1.99044586	0.55643879	0.16025641
contig_2280	0.52648898	0.72392234	0.7568279	0.46544934	0.84566596
contig_2281	1.76245211	1.99233716	2.22222222	1.76245211	0.99693252
contig_2282	1.8922853	2.65804598	2.47452693	1.9650655	1.99851962

contig_2283	0.96510765	0.96510765	1.11358575	1.1878248	0.81799591
contig_2285	0.42967631	0.99920064	0.87122243	0.82616179	0.23595608
contig_2286	0.93023256	1.70940171	1.78432894	1.02604578	1.54083205
contig_2287	0.59259259	0.74794316	2.31335436	2.37037037	3.40136054
contig_2289	0.67888663	2.32717317	2.89952798	2.5273224	2.46406571
contig_2290	1.28205128	1.34831461	1.85185185	0.78796562	1.20824449
contig_2291	1.52158528	1.20268836	1.3718947	0.76708508	0.70052539
contig_2293	1.29240711	1.29449838	1.18662352	0.70118662	0.56148231
contig_2296	2.43562978	1.88022284	3.22580645	1.37283237	3.71959943
contig_2297	0.96618357	2.12765957	1.73913043	1.64251208	0.79522863
contig_2298	2.34042553	2.12464589	1.84397163	1.91082803	1.72026926
contig_2299	1.6886931	2.72479564	2.31958763	2.87128713	1.43988481
contig_2300	1.60744501	1.86125212	5.48829701	1.78117048	1.44189992
contig_2302	2.78422274	3.80116959	3.20284698	5.3343949	3.41085271
contig_2303	1.22977346	1.79487179	1.19047619	1.12881806	0.53262317
contig_2305	8.29493088	6.37480799	8.98617512	8.19923372	8.32684825
contig_2306	1.6025641	2.89156627	2.27088402	1.32780083	1.03016924
contig_2307	1.05973025	2.11335255	1.54738878	0.38535645	1.25240848
contig_2308	1.16959064	1.29954516	2.61980831	0.97465887	0.97465887
contig_2309	4.90196078	6.8627451	3.64341085	3.88127854	5.36659108
contig_2310	1.22324159	3.67454068	2.03735144	1.60796325	1.37614679
contig_2311	1.54798762	2.45398773	1.84804928	0.92879257	0.97508126
contig_2312	1.07223476	1.08695652	1.37513751	1.15575124	0.80645161
contig_2314	0.91603053	2.44088482	1.44927536	0.83905416	1.59090909
contig_2318	1.44927536	1.6886931	1.36739834	0.68468468	0.79767948
contig_2319	1.31782946	2.58823529	3.4029389	1.26984127	1.68776371
contig_2322	2.27120908	2.18832891	1.73680695	2.34741784	1.66666667
contig_2323	1.72987359	2.06254158	1.86418109	1.37795276	0.86666667
contig_2324	0.89649552	1.44927536	1.1409943	1.1409943	1.10826939
contig_2325	1.87878788	1.63636364	2.3030303	0.69124424	0.96969697
contig_2327	1.54830024	2.32002729	2.3255814	3.47115875	1.21580547
contig_2328	0.81757793	1.8018018	1.2244898	1.10701107	3.34014997
contig_2329	0.65502183	3.14557425	1.38888889	1.03244838	0.21802326
contig_232	2.01765448	2.46406571	2.38853503	1.73333333	1.87561698
contig_2330	3.47694633	2.95909487	2.43690165	1.11376293	2.00174064
contig_2331	1.99203187	1.02921647	1.5936255	0.86321381	1.56042497
contig_2332	1.50793651	1.34920635	1.50793651	1.26984127	1.42857143
contig_2337	1.36054422	0.84550346	1.00308642	1.5060241	1.45044319
contig_2338	1.64383562	2.37442922	1.64383562	1.00548446	1.53985507
contig_233	0.82372323	1.28205128	1.32370638	2.16836735	0.7470651
contig_2340	0.93954248	1.87678499	1.72201722	2.3255814	1.49683362
contig_2341	1.01214575	1.68690958	1.28205128	0.53981107	1.48448043
contig_2342	0.89485459	2.42966752	2.9402365	1.88438199	0.73505912
contig_2345	0.93896714	2.1693492	1.59680639	0.70546737	0.64553991
contig_2347	0.1898614	0.64393939	0.64397424	0.49788399	1.41410281
contig_2348	0.53859964	1.39393939	1.11576011	0.4211793	0.29994001
contig_2349	2.54545455	2.67295597	0.54545455	1.26811594	2.11764706
contig_234	2.0979021	5.27240773	4.17090539	2.56410256	1.73469388
contig_2350	1.47058824	2.06896552	1.25733445	1.08073744	2.16698534

contig_2351	1.1409943	1.79299104	1.62999185	0.89649552	1.30718954
contig_2354	1.27504554	1.78372352	1.40073082	0.74884793	0.30248034
contig_2355	1.3584117	1.77638454	2.71682341	1.98537095	1.46290491
contig_2358	1.67014614	2.22686152	2.64439805	0.83507307	1.39178845
contig_235	2.70018622	2.63053009	1.55577623	1.88953488	1.85873606
contig_2362	0.69605568	1.17647059	1.09739369	0.76530612	1.3726836
contig_2363	1.87969925	1.65289256	2.56880734	1.31421744	1.78023327
contig_2365	1.93637621	1.93637621	1.25086866	1.79806362	1.0252905
contig_2367	1.70212766	1.27659574	1.70212766	1.55697098	1.21428571
contig_236	1.72413793	2.88065844	3.64647713	0.6772009	2.82051282
contig_2370	0.84388186	2.25035162	1.82841069	1.54711674	1.68776371
contig_2371	0.81466395	2.03665988	1.42566191	2.03665988	0.81466395
contig_2372	1.2303486	2.26789511	2.62996942	1.2345679	1.64835165
contig_2373	0.76923077	1.32774284	1.32774284	0.43956044	1.04821803
contig_2374	0.51851852	2.00445434	2.22717149	1.11111111	1.19492158
contig_2376	1.01483216	1.56128025	1.2490242	0.70257611	1.32708821
contig_2378	0.67443286	1.43995098	1.40931373	0.708061	0.54561988
contig_2379	1.83227625	1.42857143	1.66666667	0.73583517	1.75736961
contig_237	2.00527704	1.60427807	2.50783699	0.7797271	1.98735321
contig_2380	0.81081081	1.44144144	1.62601626	0.81154193	0.90334237
contig_2381	1.7989418	1.45754119	2.09258085	1.30890052	1.06918239
contig_2382	1.48016578	0.96774194	1.48016578	0.57613169	1.03626943
contig_2383	1.73490632	0.98661029	2.24089636	0.55478502	1.75685172
contig_2384	1.11593304	1.94968553	1.42238714	1.12359551	0.47990402
contig_2386	1.87040748	3.12185297	2.99401198	2.89449113	3.9536469
contig_2387	1.11692845	1.22164049	1.11692845	0.34904014	0.66317627
contig_2389	1.98163364	1.33120341	1.30057803	1.83309214	1.79318589
contig_2390	2.58236866	1.10565111	2.62850467	0.68153656	0.81727963
contig_2392	1.66112957	2.49343832	3.37690632	0.78740157	0.78740157
contig_2393	2.6642984	4.72440945	1.85185185	1.04384134	1.58730159
contig_2396	1.68690958	2.08333333	2.22672065	5.19568151	1.01010101
contig_2397	3.31491713	2.86975717	3.31125828	3.31125828	1.85185185
contig_239	1.94931774	2.50201776	1.64705882	2.6183283	1.15089514
contig_23	1.50273224	3.09423347	2.33253589	1.78970917	1.81311018
contig_2401	1.02960103	1.47294268	1.42146411	0.6121134	0.99646416
contig_2402	1.31492439	2.36686391	2.73015873	0.85470085	1.25
contig_2406	1.08786611	3.63924051	3.476874	3.32162248	3.68353619
contig_2407	1.39130435	1.56361052	0.76425632	0.93294461	0.5800464
contig_2408	0.93984962	1.62907268	2.32323232	0.62189055	1.41712877
contig_2409	1.43198091	1.61443495	1.43312102	1.8714402	0.55732484
contig_240	1.95011338	2.42816673	0.95541401	1.4041514	1.45772595
contig_2410	0.70621469	1.41242938	1.41242938	1.27118644	1.20056497
contig_2411	1.38221154	1.43660212	1.86186186	0.71942446	0.24125452
contig_2412	1.00887813	2.00324851	1.5719468	2.10466439	1.03806228
contig_2413	2.20338983	2.25733634	3.46534653	3.2183908	4.11899314
contig_2414	1.70940171	6.40668524	7.04874835	1.43369176	1.46471372
contig_2415	0.70101647	1.15667718	1.01647389	0.42060988	0.98142306
contig_2417	0.486618	1.66261152	0.80536913	1.50223305	1.11306385
contig_2418	2.28250083	1.39313626	1.09626584	1.00603622	1.32523468

contig_2420	1.03595369	2.91970803	2.5450031	1.46788991	0.73260073
contig_2422	1.44431775	1.82823129	1.48232611	1.51739452	1.39949109
contig_2423	0.77192982	1.05337079	2.22222222	0.49122807	0.73637703
contig_2425	2.34567901	2.20700152	2.52152522	1.28205128	2.0295203
contig_2426	1.90923318	1.8639329	1.70435923	2.63076447	1.62192394
contig_2427	2.2278481	2.99401198	3.19361277	2.84552846	2.27765727
contig_2428	0.59206631	1.96451204	2.0278834	1.54577883	0.34423408
contig_2429	1.01587302	3.08502634	1.84478372	1.14285714	1.65079365
contig_2430	2.19435737	3.13479624	3.65497076	2.25080386	1.3368984
contig_2435	2.10325048	3.3203125	2.95597484	1.98336532	1.49447693
contig_2436	1.77304965	2.946593	2.36406619	0.54184226	2.42085661
contig_2437	0.82135524	1.5742642	1.36892539	0.89041096	0.54832077
contig_2439	1.23997082	1.86823992	1.75054705	0.65645514	0.9502924
contig_243	1.99306759	0.86741016	0.77669903	3.38164251	1.88305253
contig_2443	1.9291162	4.39153439	2.19858156	0.49975012	0.99071207
contig_2444	1.11111111	1.78173719	0.88888889	0.66666667	0.96296296
contig_2445	1.9253911	2.41648898	2.31316726	0.58479532	1.4907573
contig_2449	2.47933884	3.96825397	3.82395382	1.44300144	0.79365079
contig_244	0.60606061	5.81395349	1.08548168	0.9771987	0.486618
contig_2451	7.22563054	7.70279482	8.31629175	7.41017964	4.63531016
contig_2452	2.00601805	2.63641937	2.22910217	2.22634508	2.23463687
contig_2454	1.41843972	3.35345406	2.481556	1.91798942	0.58252427
contig_2458	3.62318841	2.4691358	2.89855072	1.60427807	1.17647059
contig_2459	0.80106809	1.27360562	2.18398412	0.85603113	0.56459648
contig_245	1.69354839	1.72209026	1.65333333	1.8961039	1.41723356
contig_2461	1.73913043	1.95652174	1.94805195	1.37681159	1.30434783
contig_2463	0.72874494	2.26537217	2.10355987	1.37651822	0.97087379
contig_2464	1.20240481	1.8729097	1.73680695	1.53743316	0.74829932
contig_2465	1.90723884	2.19036241	2.27882038	1.97024528	1.87110187
contig_2466	1.14526823	1.32132132	1.35396518	1.09606705	1.5485408
contig_2467	1.92439863	2.88659794	2.88659794	1.09965636	1.09965636
contig_2469	0.4606968	1.17951669	0.94909405	0.9556907	0.27829314
contig_2470	0.52910053	0.66298343	0.53387334	0.69469835	0.46040516
contig_2471	2.59365994	2.05278592	3.92156863	2.02312139	2.82485876
contig_2476	0.997921	1.39235987	1.02509721	0.74738416	1.22038765
contig_2478	1.31670132	1.61177295	2	0.84566596	0.63469676
contig_247	1.71957672	3.08988764	1.97682345	2.06489676	1.49365198
contig_2480	1.06716886	2.20125786	2.89126336	2.11267606	1.00819156
contig_2483	1.42857143	0.95642933	2.79898219	1.80250784	0.87073007
contig_2488	0.56355667	1.25313283	1.75438596	1.25078174	0.7518797
contig_2489	1.13571834	2.50403877	2.27014756	1.592719	0.60706402
contig_2492	1.13091158	1.3576779	2.45543222	1.00519931	0.71077091
contig_2493	1.26413839	2.07914152	1.40562249	1.20080053	6.55080214
contig_2494	0.96618357	2.15053763	2.7605245	1.44927536	1.19382022
contig_2495	1.10535406	1.79269883	1.58894646	2.27979275	1.07081174
contig_2497	1.30385488	3.86363636	4.58190149	2.50659631	1.5954416
contig_2498	2.11038961	2.88461538	1.94244604	1.77683014	0.88495575
contig_2499	1.85988841	2.14067278	1.66975881	0.80246914	1.85540627
contig_249	1.0373444	2.55164034	3.37763012	1.36596471	0.63920455

contig_2500	1.55826558	1.82926829	2.100271	1.69376694	0.94850949
contig_2501	1.34408602	2.13333333	1.6	1.07009096	1.2979351
contig_2502	1.07526882	2.27120908	1.68408827	1.38138138	1.67000668
contig_2503	0.60504202	1.14006515	1.28452152	0.86526576	1.78091398
contig_2504	1.61812298	2.02265372	1.45631068	1.21359223	1.05177994
contig_2505	1.33333333	1.54385965	1.12449799	1.40350877	0.9122807
contig_2506	7.87037037	12.8820961	12.0870871	9.41176471	11.5720524
contig_2508	0.96153846	1.66666667	2.30326296	0.63979527	1.41206675
contig_250	3.04054054	1.86335404	2.20820189	2.05570292	2.0979021
contig_2510	1.2371134	2.66666667	3.02405498	2.68041237	1.78694158
contig_2511	0.89903181	1.24481328	1.52143845	0.55325035	0.96818811
contig_2512	1.57342657	2.73764259	2.23993926	2.72988506	1.4957265
contig_2513	1.43570537	0.63091483	1.3732834	1.19873817	0.81148564
contig_2516	1.33928571	2.46305419	1.64609053	2.30452675	0.82508251
contig_2517	1.39176227	1.47963105	1.12082493	0.72655218	0.9204857
contig_2518	0.51567657	0.61893955	0.60253927	0.72836332	0.72463768
contig_2519	0.82768999	1.33124511	1.05740181	1.81132075	0.53231939
contig_2520	0.79681275	2.01168073	1.90288714	0.72036673	1.35317997
contig_2524	2.47619048	2.05278592	2.66666667	2.38095238	0.97751711
contig_2526	2.08830549	2.42192479	2.97845374	2.32266164	1.3165769
contig_2528	1.00334448	1.75438596	2.67558528	1.84615385	1.33779264
contig_2531	0.9596929	1.41218005	1.01010101	2.76527331	1.41034251
contig_2533	2.04081633	3.79403794	3.65853659	0.86741016	2.16802168
contig_2537	1.57635468	2.3255814	2.87698413	1.63599182	4.36507937
contig_2538	1.09289617	0.98360656	1.96721311	3.65448505	1.44284129
contig_253	1.50891632	4.07488987	2.61324042	1.375	0.60565276
contig_2540	0.80775444	2.09339775	1.35674381	1.94647202	1.64203612
contig_2541	0.60396894	2.19298246	3.34288443	1.24282983	1.1
contig_2543	1.98135198	2.44755245	1.3582343	1.40105079	1.68997669
contig_2544	7.06575074	6.43564356	9.29292929	7.12851406	7.1957672
contig_2545	3.87275242	3.91363023	5.19125683	3.29218107	3.91389432
contig_2547	3.22580645	3.56778797	4.03321471	2.20125786	2.82685512
contig_2551	0.79307859	1.22655123	1.17845118	0.38619358	0.62620424
contig_2553	1.20036934	3.46534653	3.34883721	0.92250923	0.9469697
contig_2555	6.02150538	5.93311758	6.23655914	6.57108722	7.09677419
contig_2557	2.11382114	2.94924554	2.1691974	2.56410256	1.40350877
contig_2558	2	2.14723926	2.51293422	1.48902821	1.26368997
contig_2559	0.76157001	1.06007067	1.61111111	0.29291154	0.92272203
contig_255	1.63487738	2.25711482	2.04741379	2.00381679	1.55502392
contig_2560	0.38809832	1.16429495	1.20741699	0.56058646	0.6037085
contig_2563	0.80256822	2.08667737	2.48667851	0.96308186	0.64585576
contig_2567	1.03986135	1.47441457	1.82133565	1.33333333	1.47954743
contig_2568	2.28091236	3.33333333	2.03735144	1.94518126	0.92764378
contig_256	0.66371681	3.47054076	1.64638511	1.76817289	1.8714402
contig_2570	1.78970917	2.22929936	2.21518987	2.46085011	1.50537634
contig_2571	0.83333333	2.81359906	3.15789474	3.95833333	1.99530516
contig_2573	2.81124498	3.47448426	4.26758939	4.12371134	2.2587269
contig_2575	1.4084507	2.19092332	2.81690141	2.03442879	2.50391236
contig_2576	3.50877193	4.63659148	4.13533835	2.88220551	2.63157895

contig_2577	2.57270694	3.50282486	3.2474804	1.73611111	1.79573513
contig_2578	4.60157127	2.13523132	2.71867612	1.04895105	1.2716763
contig_2579	0.87003806	0.97244733	2.28966986	0.95559303	0.75890251
contig_2581	1.22967077	1.06888361	1.3064133	0.98736177	1.42517815
contig_2582	2.04342273	1.91570881	2.29885057	1.40485313	1.27713921
contig_2583	1.99004975	2.6119403	3.52201258	1.97530864	2.36318408
contig_2584	1.33451957	2.53748558	2.67295597	1.49625935	0.99255583
contig_2585	1.62601626	1.8627451	4.00843882	1.8311292	0.39215686
contig_2586	0.83003953	1.04438642	1.78192618	1.15810675	1.3444049
contig_2588	1.80623974	3.98230088	6.01421542	3.39805825	2.01117318
contig_2589	1.10552764	1.5060241	1.80722892	1.6080402	1.90763052
contig_258	1.15163148	2.01834862	1.8018018	1.9253911	1.22850123
contig_2591	1.84372256	4.51197053	3.3492823	0.18214936	3.66071429
contig_2593	0.69444444	1.62037037	1.85185185	1.62037037	1.0428737
contig_2594	0.8057676	1.63265306	1.66261152	0.69190069	1.01437025
contig_2597	1.47679325	1.68776371	2.6371308	1.16033755	1.26582278
contig_259	0.8953168	2.1043771	2.33693757	1.56889495	1.36200717
contig_2600	0.53609721	1.88948307	0.87552341	0.71230708	0.95490716
contig_2602	1.29171152	2.5974026	1.82207931	1.51843818	1.9019019
contig_2605	0.92936803	2.12121212	1.83936235	1.84126984	1.10091743
contig_2606	1.28654971	2.16271885	2.11267606	0.53937433	1.29107981
contig_2608	1.78117048	3.56234097	2.67175573	2.54452926	1.14795918
contig_260	1.13519092	2.27038184	2.47933884	0.64850843	0.50847458
contig_2610	1.63170163	1.98135198	1.63170163	1.28205128	1.86480186
contig_2611	1.0936757	1.42653352	0.99857347	0.61816453	0.52380952
contig_2612	0.87209302	4.16666667	1.47058824	0.93209055	1.91570881
contig_2613	3.79310345	6.98529412	2.42825607	3.55392157	4.41176471
contig_2614	1.42095915	2.38095238	1.65425972	2.14094558	2.17983651
contig_2615	1.78326475	1.67832168	3.42935528	2.33196159	1.37174211
contig_2616	1.16033755	2.64270613	2.32804233	2.13675214	1.85995624
contig_2617	1.5920398	2.58706468	1.29611167	1.49253731	1.5936255
contig_261	2.65957447	1.9019019	2.63424519	0.94339623	0.65252855
contig_2620	0.87527352	1.23997082	1.43964563	1.38282387	0.58479532
contig_2622	1.66666667	2.16322517	2.08333333	0.9375	1.5625
contig_2623	1.73745174	2.76292335	2.87141074	2.26950355	1.07430618
contig_2624	0.52219321	2.37467018	1.74064404	1.58572844	1.74978128
contig_2629	2.29885057	2.29885057	1.49425287	1.37931034	1.6091954
contig_2631	1.86823992	1.91740413	0.98328417	1.03244838	1.48148148
contig_2632	0.69084629	2.89017341	3.7037037	1.70068027	2.31164384
contig_2633	1.79324895	2.00421941	2.74261603	0.63559322	1.47679325
contig_2634	1.58415842	2.76134122	3.1031031	2.04498978	0.95846645
contig_2636	1.41297367	1.67417901	4.31145431	1.15755627	1.43755615
contig_2639	0.98087298	2.31350331	1.47198481	2.3573786	2.73455917
contig_263	0.38338658	1.25335721	1.95016251	5.50314465	1.55388471
contig_2640	1.67189133	2.08986416	2	1.2539185	1.56739812
contig_2642	2.25988701	3.5971223	3.92156863	2.93255132	1.59474672
contig_2643	0.54035799	0.97840756	0.7775524	0.84430935	0.4048583
contig_2644	2.13365539	2.21417069	1.85185185	1.08695652	1.08695652
contig_2645	1.21673004	3.200883	1.74639332	1.91277735	2.49140893

contig_2646	3.95327942	2.8358209	3.02445302	3.71747212	2.98013245
contig_2647	1.87903699	2.31454006	2.16277746	0.4664723	0.92646207
contig_2648	1.31147541	0.32786885	1.53005464	1.09289617	1.42076503
contig_2649	0.93196645	3.02222222	2.79069767	0.98654709	3.44827586
contig_264	1.44546649	1.81818182	2.4691358	0.82406263	1.22164049
contig_2650	0.5628977	0.66046967	0.46477495	0.48947626	0.63647491
contig_2651	1.47239264	2.07064555	2.45098039	3.00120048	1.34474328
contig_2655	3.18362706	3.45098039	4.17482061	0.72222222	2.19378428
contig_2656	1.24843945	2.62172285	2.24719101	1.87265918	1.62297129
contig_2658	0.67854114	1.88679245	1.90972222	0.64683053	0.98039216
contig_2659	2.12971926	3.22896282	3.01263362	1.76470588	1.18110236
contig_265	2.07253886	7.64872521	4.34310532	1.84108527	4.65631929
contig_2661	1.1299435	2.82485876	2.64993026	0.98039216	1.47058824
contig_2666	0.22296544	1.99600798	1.53698367	3.02114804	0.86956522
contig_2668	0.65666041	2.43445693	0.80280983	1.21212121	0.80385852
contig_2669	0.72742833	1.2303776	1.27478754	1.16580311	0.37942664
contig_266	0.85592011	1.72143975	2.21402214	1.64654226	1.29449838
contig_2673	1.68439716	3.80228137	6.77248677	3.71593724	7.18321227
contig_2676	1.45322434	1.83150183	2.26449275	1.19047619	0.76408787
contig_2679	2.48640249	1.94250194	2.29799852	0.62160062	1.10497238
contig_2680	2.2556391	3.37078652	3.74531835	1.5037594	2.63157895
contig_2681	1.74471993	2.94396961	2.4024024	1.13960114	1.13960114
contig_2682	1.89018902	1.82815356	2.22816399	2.43902439	1.62895928
contig_2683	3.01142264	2.21260221	3.55249205	3.14875136	1.87376726
contig_2685	0.79365079	1.16046419	1.1466983	0.60096154	0.31570639
contig_2688	1.8579235	2.27272727	2.97029703	1.77187154	0.32154341
contig_268	1.22237894	3.32843857	2.06508135	1.48809524	1.12612613
contig_2691	0.36452005	1.68758113	2.02511138	1.42450142	0.52652896
contig_2692	2.03252033	3.25203252	2.74390244	2.23577236	1.42276423
contig_2696	1.91780822	6.72268908	5.41062802	2.47933884	3.33333333
contig_2697	1.51946819	2	2.53968254	1.2345679	1.48148148
contig_2699	1.28205128	1.38568129	1.72413793	0.61728395	1.71821306
contig_269	2.41254524	1.86666667	2.83085633	2.20326937	1.5503876
contig_26	1.68539326	2.08986416	2.14183722	1.58075601	1.3986014
contig_2702	1.84225676	1.80878553	2.77463194	1.23734533	0.66815145
contig_2704	1.92307692	1.26849894	1.89274448	0.43478261	1.7130621
contig_2705	1.2775842	2.5	2.77777778	1.25	1.74216028
contig_2707	1.17449664	1.84563758	2.06935123	1.34228188	0.8815427
contig_2708	1.78970917	1.97726149	2.08688245	1.48548278	0.93736685
contig_2709	0.85585586	2.25225225	2.56756757	0.63063063	1.62162162
contig_270	1.35802469	2.3364486	2.58531541	0.34995626	2.03644159
contig_2711	2.7100271	3.52303523	3.38753388	2.01729107	3.25203252
contig_2713	1.10987791	1.39372822	2.44498778	1.7699115	1.6025641
contig_2714	4.36205016	5.50055006	4.90196078	2.74621212	2.2875817
contig_2716	2.8320971	1.12359551	2.09035738	0.98107919	0.92497431
contig_2717	1.36054422	2.49433107	1.4739229	1.92743764	1.58730159
contig_2718	2.04081633	2.34454638	2.95616718	2.16271885	1.12130479
contig_2719	2.31481481	3.49288486	1.85185185	2.31481481	2.00617284
contig_271	2.31958763	1.69172932	1.46067416	2.27272727	2.87356322

contig_2722	1.31086142	2.51937984	2.74656679	0.43695381	1.74496644
contig_2723	0.56058646	1.16529996	1.59551531	0.77619664	0.86244071
contig_2725	0.45433894	1.20925342	1.2715713	0.63578565	0.49977283
contig_2727	1.17521368	1.06951872	1.6025641	2.00945626	0.45924225
contig_2728	0.93509351	1.9433648	3.22394934	2.16894977	1.7826337
contig_2729	1.86741363	1.79299104	1.96721311	0.52687039	0.87527352
contig_272	1.4721346	2.7027027	2.54065041	1.64251208	1.49031297
contig_2730	2.30352304	2.84552846	2.43902439	2.30352304	0.81300813
contig_2732	1.74418605	4.35806832	3.47826087	1.06589147	2.72108844
contig_2736	1.97368421	3.24909747	2.31023102	0.66225166	0.66445183
contig_2737	1.54405086	2.95588904	2.04081633	0.70060719	3.36976321
contig_2739	1.99004975	1.87265918	3.88471178	1.87969925	1.49253731
contig_273	1.56402737	0.997151	1.52733119	0.73710074	2.02702703
contig_2740	0.86580087	1.55642023	0.89171975	0.83194676	1.10403397
contig_2741	1.20614035	1.0845987	2.21483942	2.36907731	1.01237345
contig_2742	1.1627907	1.32890365	1.60875161	1.66112957	0.94130676
contig_2743	2.49042146	4.02298851	3.25670498	2.10727969	2.87356322
contig_2744	2.53164557	2.74261603	3.48101266	2.10970464	1.89873418
contig_2746	0.71647902	1.80094787	2.19378428	1.70886076	0.69422531
contig_2747	2.82485876	4.02298851	4.97076023	2.63157895	2.92397661
contig_2748	2.00250313	3.77002828	2.48161765	1.06269926	1.30111524
contig_2749	3.31588133	2.48538012	3.14136126	0.99009901	2.25694444
contig_2750	2.45454545	2.10526316	2.58548791	0.76335878	3.26576577
contig_2751	0.44585987	1.5970516	2.14646465	1.2145749	1.72307692
contig_2753	1.50943396	3.74531835	2.14646465	3.24594257	1.91815857
contig_2754	0.66225166	1.06382979	0.93085106	1.23620309	1.20913569
contig_2757	2.86659316	0.98463962	1.01156069	0.99651221	0.81585082
contig_2758	4.01002506	3.50877193	4.39276486	3.33333333	0.55096419
contig_275	1.85546875	0.8841733	1.17244748	2.05761317	0.67567568
contig_2760	2.05128205	3.58974359	3.24786325	1.36752137	2.39316239
contig_2761	2.79503106	2.57201646	1.77083333	2.93501048	2.50783699
contig_2762	1.15334207	2.07501995	1.7557652	1.17449664	1.67758847
contig_2763	2.06489676	2.60999254	3.17343173	3.62962963	3.01675978
contig_2765	1.62907268	3.22580645	1.99004975	1.74346202	2.125
contig_2767	4.97881356	4.21940928	3.27004219	3.1372549	2.59179266
contig_2768	2.07715134	1.97823937	5.0273224	0.89186176	1.08803165
contig_2771	2.46125798	0.85910653	1.24164279	1.83823529	0.56710775
contig_2772	4.19947507	5.19877676	5.04201681	2.16049383	2.16049383
contig_2773	1.28585559	1.45379024	2.80830281	1.98216056	0.71258907
contig_2774	1.71990172	2.36406619	2.36406619	1.80505415	1.05509965
contig_2776	0.80515298	1.86795491	1.49309444	1.22265122	0.73157006
contig_2778	6.05664488	7.76614311	5.44662309	1.71379606	6.17928634
contig_2779	1.08882521	2.0045819	1.77777778	0.5730659	1.26002291
contig_277	1.27118644	0.77220077	2.76292335	0.93348891	2.68041237
contig_2780	1.56794425	3.23741007	2.78503046	2.21653879	1.07421875
contig_2783	1.59817352	1.87214612	2.31404959	1.23287671	1.09439124
contig_2784	1.97132616	2.95698925	2.15053763	1.97132616	1.46923783
contig_2785	11.1471861	11.0752688	11.0722611	11.3785558	11.3043478
contig_2786	2.88808664	2.8340081	2.0383693	0.24125452	1.09356015

contig_2791	2.45566166	1.61290323	2.3222061	2.20264317	0.58224163
contig_2792	1.39784946	1.93548387	1.93548387	1.50537634	1.50537634
contig_2795	0.91463415	1.52439024	1.42276423	1.07858243	1.7167382
contig_2796	1.07393639	1.7783292	2.23325062	1.39475909	0.97997444
contig_2797	1.18371212	1.657982	1.69731259	1.65016502	2.02734559
contig_27	3.43170899	2.77264325	2.14876033	1.36518771	1.37659784
contig_2800	1.01010101	2.61603376	2.3628692	2.27272727	2.70042194
contig_2801	0.84121977	4.09924488	5.53633218	0.7486631	1.60771704
contig_2803	2.28070175	1.26126126	3.50877193	2.98245614	2.45614035
contig_2804	1.28301887	1.45934677	2.050581	0.5468216	0.69651741
contig_2805	0.74786325	2.01900238	2.50260688	1.6025641	1.14702815
contig_2806	1.13580247	1.78041543	1.86859554	1.25223614	0.84439083
contig_2808	1.06075217	1.54440154	1.28700129	0.32144005	0.80906149
contig_280	1.21506683	2.48756219	3.125	1.2300123	2.11382114
contig_2810	0.75662043	1.76433522	2.33144297	1.57529931	0.94517958
contig_2811	1.16375727	2.61136713	1.85950413	1.75438596	2.55681818
contig_2812	2.05128205	3.60046458	2.31660232	1.73410405	2.05920206
contig_2813	1.0139417	3.01318267	1.78435839	1.37825421	1.37903861
contig_2814	2.01698514	2.33545648	1.48619958	1.00334448	1.33460439
contig_2815	2.31607629	4.0446304	3.21100917	1.34408602	2.14067278
contig_2816	2.68138801	3.46878097	2.31362468	1.3681592	1.86046512
contig_2818	0.94228504	1.78784267	1.58450704	1.52761457	2.34146341
contig_2819	0.8815427	0.93818985	1.48514851	0.88642659	1.26582278
contig_281	0.4048583	2.29776519	1.184573	2.7854385	0.85240018
contig_2820	2.10526316	1.13798009	1.68421053	1.8245614	1.30260521
contig_2822	2.12014134	3.8869258	2.00235571	0.70754717	1.1778563
contig_2823	2.04081633	1.84824903	2.62390671	1.77404295	1.45985401
contig_2825	1.36054422	2.52672498	2.12355212	1.75438596	1.8907563
contig_2829	0.72992701	1.82481752	3.28638498	2.45901639	0.85158151
contig_282	1.66773573	3.33745365	3.28467153	4.28870293	0.19493177
contig_2830	1.73333333	2.92056075	2.85006196	2.4	2.29468599
contig_2831	6.46583395	6.25	4.77223427	6.2150056	4.91345617
contig_2832	0.99502488	3.80952381	2.26190476	2.50284414	1.24716553
contig_2833	2.02380952	3.0952381	1.90703218	1.70940171	0.7290401
contig_2834	1.10132159	3.83091149	1.7865323	0.35257823	0.8377425
contig_2835	1.06382979	1.63120567	1.8089725	1.20567376	1.16175156
contig_2837	0.72604066	0.96873624	1.18733509	1.30548303	0.8355321
contig_2839	1.51658768	4.17940877	2.23152022	2.26628895	1.84108527
contig_283	2.66666667	3.00842359	0.72463768	3.03030303	2.15439856
contig_2840	4.45292621	4.52830189	3.04232804	2.5974026	1.60213618
contig_2841	9.07911803	8.4306096	10.6355383	8.60495437	7.29166667
contig_2842	1.51843818	1.91740413	2.21238938	2.07100592	2.50737463
contig_2843	1.14638448	1.23893805	1.51380232	2.48796148	0.47732697
contig_2844	2.8150134	2.14190094	3.61445783	2.14477212	1.07095047
contig_2845	0.54274084	1.30434783	2.37288136	0.47554348	0.7826087
contig_2848	1.92102455	2.18579235	2.02020202	1.93939394	1.93939394
contig_2849	2.37324703	2.80474649	2.91262136	2.69687163	1.94174757
contig_284	0.8	0.68807339	1.41010576	1.05820106	0.80385852
contig_2850	1.565762	2.70833333	3.33680918	1.35135135	0.96359743

contig_2853	1.36570561	2.90979631	2.05026455	0.60882801	1.33868809
contig_2854	1.68195719	2.57575758	2.42424242	1.41700405	1.81818182
contig_2855	1.71821306	4.66321244	5.79196217	4.0091638	5.56844548
contig_2857	1.97530864	2.4691358	2.71604938	1.97530864	1.72839506
contig_2858	0.84985836	2.57414661	1.06085985	1.22836404	1.11731844
contig_285	2.59067358	1.32850242	1.3253012	1.52207002	2.23367698
contig_2860	0.63636364	2.40847784	1.71790235	1.83044316	0.29527559
contig_2861	1.91693291	1.70394036	2.342918	0.69860279	0.53304904
contig_2863	1.2345679	3.28282828	3.28282828	2.53807107	3.29949239
contig_2864	1.27536232	2.14492754	1.79710145	1.10144928	2.69461078
contig_2865	0.4460665	1.62206002	1.68918919	1.25709651	0.93306288
contig_2869	2.65363128	2.39099859	2.92887029	2.5	1.68067227
contig_2870	3.57518402	6.97674419	5.81140351	4.62962963	2.11193242
contig_2873	2.22405272	3.34890966	2.65957447	2.27743271	1.89054726
contig_2874	9.87780041	10.5105105	7.75510204	9.30930931	10.8108108
contig_2875	2.66666667	2.26666667	2.8	2.58397933	2.66666667
contig_2877	0.9556907	1.73992674	2.10622711	0.91074681	1.37362637
contig_2879	1.50862069	1.41843972	1.65229885	1.75054705	0.9018759
contig_287	1.33037694	3.63180964	2.98804781	3.41880342	2.82485876
contig_2881	1.83792815	2.33918129	2.92397661	1.93115029	1.58862876
contig_2882	2.45901639	1.82315406	2.82331512	1.09289617	1.54826958
contig_2883	1.29310345	1.97607904	2.15605749	1.44063587	1.92634561
contig_2886	1.59489633	3.96825397	3.61552028	3.01826847	2.06841687
contig_2887	2.22222222	3.18979266	3.03030303	2.06677266	2.06349206
contig_2888	1.91693291	3.83386581	3.19488818	2.12992545	1.27795527
contig_2889	1.17762512	2.65392781	1.27388535	1.16772824	1.80467091
contig_2892	1.3434089	3.13559322	2.11640212	1.67926113	1.59663866
contig_2894	2.31023102	2.74914089	2.87878788	3.07971014	1.2195122
contig_2895	1.06589147	1.35790495	1.84108527	1.25968992	0.7751938
contig_2896	2.61096606	1.73611111	1.82291667	2.09059233	0.95735422
contig_2898	1.85654008	2.6293469	2.29591837	1.0178117	1.18744699
contig_2900	2.32371795	3.17073171	3.49593496	2.90556901	1.74129353
contig_2901	1.41509434	2.04402516	2.20125786	1.8081761	1.01960784
contig_2903	1.80412371	3.46585117	2.57731959	1.55709343	0.82712986
contig_2906	1.0178117	1.33333333	0.9122807	1.3968254	0.68965517
contig_2907	1.07883817	2.4691358	2.21130221	0.66833751	0.92827004
contig_2908	1.7418844	2.82485876	3.61519608	2.73631841	1.79591837
contig_2911	1.24031008	3.96825397	3.60153257	1.82539683	1.46266359
contig_2912	1.10062893	1.97005516	1.04501608	1.68269231	0.95465394
contig_2913	2.33766234	3.72983871	1.47167035	0.29828486	1.83727034
contig_2914	1.52008686	2.18914186	3.0647986	2.09973753	0.89365505
contig_2915	0.55432373	1.09606705	1.46950771	1.29449838	0.58287796
contig_2916	3.99724328	3.64779874	3.30417881	1.46341463	0.32948929
contig_2918	3.35195531	2.64705882	2.27272727	2.37868696	1.49812734
contig_2919	1.63551402	1.62412993	1.59453303	1.15473441	1.08275329
contig_291	1.09119252	3.40993328	1.23076923	5.14403292	2.31788079
contig_2920	1.94420964	1.67158309	3.97932817	0.95497954	1.19313945
contig_2921	1.23809524	1.71428571	2.12527964	1.61904762	1.61904762
contig_2922	10.5090312	10.3475513	10.3448276	9.79532164	9.58036421

contig_2923	0.9478673	1.28514056	1.06635071	0.9478673	0.55445545
contig_2926	1.57480315	1.50922303	1.35062129	0.99473376	0.86021505
contig_2927	2.75624462	4.75812847	4.5021645	2.3364486	2.14285714
contig_2928	1.16175156	1.96078431	1.90562613	1.16175156	1.16175156
contig_2930	0.84425036	1.60095579	1.25651968	1.31358968	0.90183836
contig_2931	3.30396476	3.0839895	1.98237885	0.81481481	0.97323601
contig_2932	0.84611317	1.53468386	1.44478844	2.15716487	1.77638454
contig_2933	1.35021097	2.63374486	2.96296296	1.48148148	0.91743119
contig_2934	1.1741683	1.56402737	1.27077224	1.46771037	0.39138943
contig_2935	1.59489633	2.08	2.79106858	1.44115292	1.03916867
contig_2937	2.30607966	2.30607966	2.7700831	1.36268344	3.02506482
contig_2938	2.7027027	5.32879819	4.60157127	2.7027027	2.46085011
contig_2939	1.31845842	0.41109969	1.28968254	0.25873221	1.03950104
contig_2940	1.47895336	10.2702703	1.67410714	4.10798122	3.53200883
contig_2942	1.41843972	3.37301587	2.8650647	2.50501002	1.78739417
contig_2943	2.42718447	4.27860697	4.11089866	0.89552239	2.67034991
contig_2944	1.33928571	1.33928571	1.22905028	0.78125	1.11731844
contig_2945	1.352657	2.89855072	2.12560386	0.96618357	1.54589372
contig_2946	1.06544901	2.13089802	0.87527352	0.91324201	1.63204748
contig_2947	1.80878553	3.38283828	3.10077519	2.72277228	1.98511166
contig_294	0.89820359	1.83615819	1.47255689	1.33587786	0.48780488
contig_2951	2.18855219	3.41951626	3.53143842	3.61757106	0.51724138
contig_2952	1.4619883	3.50877193	2.76243094	1.4619883	2.33918129
contig_2956	1.00830368	2.21027479	2.03106332	2.38500852	3.75886525
contig_2958	0.89285714	1.60570919	1.42602496	0.53715309	0.4456328
contig_2959	0.72169599	1.1732852	0.90211998	0.54127199	0.63233966
contig_295	1.20160214	3.23660714	2.00873362	1.43442623	0.81967213
contig_2960	1.74418605	2.16126351	2.32172471	1.98019802	2.97029703
contig_2963	1.208981	2.7638191	1.88370188	2.72108844	2.26086957
contig_2964	1.68463612	1.98087432	2.41046832	0.98199673	2.16450216
contig_2966	1.45259939	1.26705653	2.67584098	1.48148148	1.58353127
contig_2968	1.3986014	4.49010654	3.00870942	0.39032006	1.20192308
contig_2973	1.07899807	1.33149679	1.23314066	1.38888889	0.73245952
contig_2974	1.26582278	1.58227848	1.05152471	1.26715945	1.89873418
contig_2975	0.82051282	1.89520624	2.00668896	0.66006601	1.6722408
contig_2977	2.05570292	2.29885057	3.24718357	4.12105602	4.60992908
contig_2978	1.67046317	1.67173252	1.45148969	1.89969605	1.27340824
contig_2979	1.93905817	2.30840259	2.12373038	1.84672207	1.01569714
contig_2980	1.60575858	2.12765957	2.12389381	1.52542373	1.17434508
contig_2981	1.39405204	1.57845868	3.34261838	0.64995357	1.20705664
contig_2983	1.525941	2.88557214	2.77777778	2.2977023	1.03092784
contig_2985	0.9629273	1.58577607	1.72413793	0.90996169	1.17647059
contig_2988	1.72265289	1.20585702	1.5503876	1.72265289	0.94745909
contig_2989	1.77993528	2.50809061	2.75080906	0.88996764	1.05177994
contig_2992	1.1579818	1.41196013	1.49501661	0.32921811	0.72405471
contig_2993	1.98473282	1.90694127	2.05949657	1.11111111	0.91324201
contig_2994	0.90834021	1.40379851	1.89768977	1.32340778	0.99009901
contig_2995	1.30718954	1.83908046	1.7369727	1.60183066	0.92879257
contig_2997	1.58450704	2.77777778	2.32131949	1.5037594	1.5130674

contig_2998	2.64550265	3.80194518	1.7989418	2.85714286	1.37711864
contig_2	2.94530154	4.84913793	2.5095057	5.33472803	0.68493151
contig_3000	1.65934602	2.08636584	2.06185567	0.80058224	0.86393089
contig_3002	2.04444444	3.02222222	2.48888889	3.11111111	1.6
contig_3003	2.14338507	3.72807018	3.34029228	2.07407407	1.84501845
contig_3005	0.73800738	1.41451415	1.16421569	1.04166667	1.4084507
contig_3006	3.35195531	2.80112045	3.8425492	4.35606061	1.12044818
contig_3007	1.34600158	1.82106097	1.90023753	2.53365004	1.26682502
contig_3008	1.0620915	1.31477185	1.53473344	0.64935065	0.25531915
contig_3009	1.0019084	1.33155792	0.92592593	1.02678571	0.66137566
contig_3011	1.44927536	2.74599542	3.12738368	0.68649886	1.60183066
contig_3012	0.79554495	1.23309467	0.91487669	0.39777247	0.39777247
contig_3014	1.8018018	2.05158265	2.21975583	2.01450443	1.46627566
contig_3016	1.28726287	1.4059754	1.742552	1.69952413	0.42857143
contig_3017	1.91693291	5.30785563	2.23642173	1.70575693	1.38445154
contig_3018	1.60836349	2.09087254	2.17129071	1.99161426	1.53738644
contig_3019	0.83062477	0.93896714	1.19176598	0.75839653	0.57782593
contig_3020	0.77220077	1.46239554	1.22265122	1.12016293	2
contig_3024	1.48619958	2.33258089	2.55831452	2.25733634	1.77383592
contig_3027	1.1023622	2.20385675	1.92837466	1.72921266	0.78864353
contig_3031	1.98198198	1.57480315	1.50918635	0.65876153	1.86011905
contig_3033	1.33064516	1.39082058	0.94185094	1.30879346	0.98159509
contig_3034	0.98619329	2.56410256	2.95857988	0.88757396	0.999001
contig_3039	1.17449664	1.6	1.92629816	1.27659574	1.2455516
contig_3040	0.59405941	1.25495376	1.81311018	2.06185567	2.14352283
contig_3041	2.12962963	3.50877193	4.45682451	2.34741784	2.70018622
contig_3042	1.26931567	1.65289256	1.27777778	0.86071987	0.61983471
contig_3043	1.71192444	2.124834	1.63398693	1.14379085	0.96885813
contig_3044	1.27853881	1.82648402	1.55251142	1.09589041	0.91324201
contig_3047	2.07156309	3.0075188	3.94366197	1.69014085	2.141527
contig_3048	1.2962963	1.91358025	1.72839506	1.2962963	0.92592593
contig_3049	2.00892857	1.65016502	3.25443787	2.35414534	1.7130621
contig_304	0.59656972	1.07705054	1.73053152	1.31964809	0.53003534
contig_3050	1.03244838	1.47710487	1.32743363	1.38323841	1.7397882
contig_3051	1.82370821	1.51975684	1.4213198	0.70921986	1.41843972
contig_3053	2.62529833	2.14285714	2.72435897	2.21579961	2.40384615
contig_3054	2.56410256	1.46092038	4.16666667	2.94117647	2.43716679
contig_3055	0.70528967	0.94024871	0.90744102	1.44467935	1.37711864
contig_3057	1.80436847	2.46212121	2.84090909	1.60984848	1.91666667
contig_3059	1.31324836	2.12437234	1.62224797	1.09995218	0.84974894
contig_305	1.73010381	3.34528076	1.40986908	0.55788006	0.9009009
contig_3062	1.31926121	4.48548813	4.15194346	3.43007916	1.49911817
contig_3064	0.91342335	1.71042164	1.34920635	0.76628352	1.15262321
contig_3065	0.83798883	0.92850511	0.83410565	0.74418605	1.62107396
contig_3066	3.13390313	2.85714286	3.13390313	4.58452722	2.22929936
contig_3067	2.13089802	2.10843373	2.30923695	0.80321285	1.6064257
contig_306	1.26903553	3.29545455	2.52964427	3.63408521	0.61728395
contig_3070	0.63643596	2.66666667	2.3923445	2.15311005	2.22752586
contig_3072	1.02442868	2.07501995	2.75807723	2.0979021	0.46765394

contig_3074	0.97150259	4.17801998	1.98019802	2.41545894	2.52631579
contig_3078	1.79127726	1.26689189	1.43702451	1.45038168	1.88111362
contig_3079	0.98911968	1.68150346	1.68150346	0.89020772	1.63043478
contig_307	1.58730159	2.17755444	2.19619327	0.78125	0.21881838
contig_3080	3.5746202	4.01427297	4.27807487	1.82648402	2.85459411
contig_3081	4.86348123	4.63121784	5.77777778	5.34979424	4.18803419
contig_3083	2.02578269	2.02578269	2.20994475	1.65898618	1.38121547
contig_3085	1.4571949	2.9972752	2.03359859	0.75973409	1.04861773
contig_3086	1.36054422	3.36817654	2.4534687	1.52931181	1.33060389
contig_3088	1.0989011	2.08540218	2.17596973	1.21836926	1.13636364
contig_308	1.96850394	2.31481481	1.53846154	0.24813896	0.7183908
contig_3091	1.57068063	0.65093572	1.32978723	0.39525692	1.27853881
contig_3092	0.73839662	1.69491525	1.83823529	1.96078431	3.06010929
contig_3093	0.91590341	2.34741784	3.23475046	3.7254902	4.91195551
contig_3094	4.1556145	2.29276896	2.71317829	3.91644909	2.56410256
contig_3095	3.19361277	1.70340681	2.9029029	2.3023023	2.80561122
contig_3096	2.39463602	4.98084291	4.02298851	1.14942529	2.40153698
contig_3097	1.05485232	1.16033755	2.10970464	1.05485232	1.58227848
contig_3098	1.96656834	2.06489676	2.24358974	1.37659784	1.08161259
contig_3100	1.30187144	1.50421179	2.00320513	1.16812683	2.39059968
contig_3101	1.80904523	3.25203252	4.60093897	2.02224469	2.29540918
contig_3102	0.85470085	1.54440154	3.51906158	1.67264038	1.2392755
contig_3106	1.0270775	3.5480859	3.36134454	2.80112045	1.0270775
contig_3107	3.10880829	4.47761194	3.93374741	0.82815735	1.19760479
contig_3109	5.51948052	4.85436893	7.4433657	1.29449838	3.58306189
contig_3111	3.51537979	4.29601756	3.44935717	2.88241074	2.84435922
contig_3112	2.08333333	2.48328558	2.10124164	2.9608405	3.45849802
contig_3113	0.97402597	1.62337662	1.7699115	0.88757396	1.73160173
contig_3116	1.19156737	2.1978022	2.01465201	1.46520147	1.37362637
contig_3117	1.67339873	2.81609195	3.24976787	1.90641248	1.76322418
contig_3118	1.34228188	1.2464046	1.81992337	1.14942529	1.62835249
contig_3121	1.6374269	3.15789474	4.21052632	2.23004695	2.00471698
contig_3123	0.76923077	1.31672094	0.86713698	0.8377425	0.679983
contig_3124	0.8105802	2.16768916	1.19352089	0.98039216	0.53966541
contig_3125	1.8018018	2.07207207	2.25225225	0.99099099	1.70940171
contig_3127	0.98039216	1.31578947	1.06761566	0.80357143	2.76292335
contig_3128	2.45746692	3.58829084	2.36071766	1.61290323	1.32200189
contig_3129	1.86219739	3.44506518	3.44506518	1.86219739	1.58286778
contig_312	1.1190234	2.96961326	1.07889413	0.87660148	0.48721072
contig_3130	1.67189133	3.87840671	3.6687631	2.62054507	3.33333333
contig_3133	1.21012101	1.81518152	1.81518152	0.8255366	1.21012101
contig_3136	1.48478099	1.33679911	1.29342203	0.48112509	1.2345679
contig_3138	1.57004831	1.93236715	1.69082126	1.08827086	1.44927536
contig_3139	1.30718954	1.86741363	1.86741363	1.68067227	1.34408602
contig_313	4.27966102	4.05522002	2.1685254	7.22222222	2.79441118
contig_3142	2.80764636	2.13396562	3.0781693	2.68948655	1.578354
contig_3143	3.13390313	2.22222222	2.42954325	1.45772595	1.62368672
contig_3144	2.69953052	2.19178082	1.955105	1.41467728	0.92250923
contig_3145	1.18973075	4.47035957	5.88662791	4.18227216	2.54385965

contig_3148	1.14503817	1.77777778	1.65079365	1.20634921	1.14285714
contig_314	1.72413793	1.5548645	2.27882038	0.79893475	0.73839662
contig_3150	1.93720775	1.87793427	2.66040689	1.38248848	2.30708035
contig_3151	1.18803769	1.96560197	2.01895344	1.65016502	0.61000407
contig_3153	1.14394662	1.74927114	1.5920398	0.98619329	0.68292683
contig_3154	2.01612903	2.02166065	2.04498978	1.51209677	3.96551724
contig_3155	0.58805323	1.02135562	1.08325596	0.37105751	0.24397682
contig_3156	0.79217148	1.8079096	2.02020202	1.53203343	0.72604066
contig_3157	1.33178923	1.74927114	1.15740741	1.50987224	0.98894706
contig_3161	1.97585071	2.19298246	2.85087719	1.4254386	0.98684211
contig_3162	0.44174489	2.91529153	2.78384279	2.25770925	1.52755046
contig_3163	2.3255814	2.72727273	2.02224469	2.52525253	2.38568588
contig_3165	1.8579235	2.39651416	3.48583878	3.26797386	1.52008686
contig_3167	0.55555556	2.43407708	1.70109356	1.24756335	0.56703119
contig_3169	1.5625	1.40318054	1.8639329	0.95328885	1.97740113
contig_316	1.41065831	1.5	1.86219739	1.04529617	1.72955975
contig_3170	1.20481928	1.20643432	2.21327968	0.82644628	0.95011876
contig_3171	1.69779287	2.94284097	3.58612581	2.75482094	2.68700073
contig_3173	1.41150923	2.63901979	3.90879479	1.69971671	1.6286645
contig_3175	2.787068	4.06504065	2.56410256	3.14606742	3.62811791
contig_3176	1.17845118	2.11335255	1.91780822	1.51515152	1.36986301
contig_3177	1.28205128	1.97238659	1.87376726	1.18343195	1.57790927
contig_3178	0.97789116	1.61564626	1.40306122	1.06292517	0.80782313
contig_3179	1.59074476	1.22921186	2.74765004	1.15690528	1.85185185
contig_3180	0.70310003	1.08695652	0.92948718	1.09051254	0.50922979
contig_3181	1.09375	2.87769784	1.18110236	0.47021944	1.83266932
contig_3186	0.97680098	1.57004831	1.58730159	0.72245635	1.02040816
contig_318	0.97560976	2.49433107	2.70602706	2.4516129	2.29357798
contig_3190	1.20585702	1.98618307	1.55440415	0.57613169	0.57613169
contig_3191	2.11640212	2.67558528	3.03030303	1.09090909	1.71957672
contig_3192	0.73302469	1.08024691	1.19598765	1.0428737	0.61728395
**contig_3193**	**1.63934426**	**1.53005464**	**1.97368421**	**1.76017602**	**0.89585666**
contig_3199	0.83857442	2.49415433	4.59501558	1.12612613	2.83018868
contig_31	2.75439939	3.68818106	2.8831563		1.15273775
contig_3201	1.22324159	1.10091743	1.02843315	0.42839657	0.73394495
contig_3205	2.9535865	3.17775571	2.88461538	1.53403643	2.08333333
contig_3206	1.1299435	3.20150659	2.91902072	1.77404295	2.054155
contig_3207	2.36928105	3.10077519	3.91156463	2.61824324	1.04166667
contig_3208	1.25786164	1.2588513	1.49371069	1.33437991	1.1820331
contig_320	10.4046243	10.2316602	5.45596259	2.01793722	2.94695481
contig_3210	0.59835453	0.97378277	1.38282387	1.07361963	0.64935065
contig_3211	1.0487677	1.51991614	1.62473795	0.47244094	0.89285714
contig_3212	1.90718373	1.78861789	2.01384519	0.6557377	1.68674699
contig_3214	1.66889186	1.66002656	1.92563081	0.73138298	1.10759494
contig_3215	1.92307692	2.31814548	4.84261501	2.40963855	1.1299435
contig_3217	0.75667065	1.67064439	1.58460668	1.23309467	0.61349693
contig_3218	1.42857143	1.21212121	3.87665198	1.23355263	0.24291498
contig_3219	1.1299435	1.67818361	2.46794872	0.91254753	0.50096339
contig_321	0.73461892	2.39190432	0.91157703	0.72016461	1.97530864

contig_3220	2.05655527	1.83387271	2.14776632	1.54241645	0.92592593
contig_3221	0.84388186	3.80952381	3.18257956	1.37457045	1.5993266
contig_3222	2.413273	1.65562914	2.00668896	0.88300221	1.00671141
contig_3224	1.35708227	4.95382032	2.86919831	2.03562341	1.6115352
contig_3225	1.9047619	2.07792208	2.07792208	1.21212121	1.3434089
contig_3227	2.64993026	1.50715901	2.76338515	0.84328883	1.81968569
contig_3228	2.83203125	1.06692532	5.04761905	2.21518987	0.86705202
contig_3229	1.15190785	1.63304515	1.55987521	0.50395968	0.45728039
contig_3230	1.6765286	1.7921147	2.7072758	1.47928994	1.38067061
contig_3231	0.87014726	2.61044177	1.45027624	0.61082024	1.76787087
contig_3232	2.31481481	2.12053571	1.98019802	1.55382908	1.44444444
contig_3233	1.46750524	2.295253	1.80133814	0.73030777	1.63170163
contig_3235	2.03359859	2.03359859	1.95035461	1.59151194	1.23784262
contig_3236	1.68539326	2.99625468	2.71535581	2.77280859	1.12464855
contig_3237	1.39917695	2.95081967	3.01302932	1.31578947	1.39917695
contig_323	1.37540453	1.35135135	1.10356537	1.74029451	0.98522167
contig_3240	1.46386093	4.16666667	3.09653916	1.93548387	1.19815668
contig_3241	0.98522167	1.71990172	1.8134715	1.32340778	1.14942529
contig_3242	1.25340599	2.93501048	2.55991285	1.42932769	1.16537181
contig_3244	1.5755329	2.80561122	4.55390335	3.60696517	1.03383459
contig_3245	2.03389831	1.90713101	2.92397661	1.42222222	2.02531646
contig_3246	1.38408304	2.65282584	2.30680507	1.49425287	1.84544406
contig_3248	0.8254717	1.53121319	1.41426046	1.23893805	2.74122807
contig_3249	1.36307311	2.10918114	2.73972603	1.48698885	1.35802469
contig_324	1.34502924	0.91420534	1.24949617	1.47727273	1.25944584
contig_3250	1.47783251	2.51322751	2.4691358	2.83950617	2.1039604
contig_3251	1.55440415	3.52053646	2.77056277	0.25974026	1.04166667
contig_3252	1.07874865	1.99275362	2.04460967	1.54888674	1.95530726
contig_3253	1.53031195	2.23398001	1.66769611	3.23339212	1.88124633
contig_3254	2.11800303	4.35510888	4.20560748	1.24416796	6.74486804
contig_3257	1.08813928	1.08843537	1.46638054	1.40338251	0.54347826
contig_325	1.13636364	1.0668164	1.88517566	0.6840796	0.98314607
contig_3260	0.8008008	2.48917749	2.70562771	1.10663984	1.73160173
contig_3261	1.22362869	1.51950719	2.01646091	1.31523123	1.39593909
contig_3263	4.58015267	4.58015267	5.05050505	4.58015267	3.05343511
contig_3264	1.38888889	4.03071017	5.26315789	1.99556541	3.46666667
contig_3265	1.2864494	3.35276968	2.38704177	1.03004292	3.95778364
contig_3267	1.12239444	1.60750167	1.57181572	2.66040689	1.63627863
contig_3268	1.04364326	2.27272727	2.69886364	1.13122172	2.22748815
contig_3271	1.3803681	2.69922879	2.74207369	1.32819195	1.3653484
contig_3273	3.9800995	4.72636816	8.33333333	4.72636816	2.98507463
contig_3274	0.66137566	1.65165165	1.75438596	0.68226121	0.78226858
contig_3275	1.6064257	1.97300104	2.7027027	1.68150346	0.60331825
contig_3277	1.54043646	2.31660232	2.6119403	1.66204986	0.56818182
contig_3278	1.57232704	1.83534347	2.04402516	1.04821803	1.41583639
contig_3279	1.28205128	2.01117318	0.89186176	1.76697957	0.71743929
contig_3282	3.67567568	3.28151986	1.57480315	2.45700246	1.96078431
contig_3283	1.04640582	2.27642276	1.31467345	1.13107119	2.00638395
contig_3286	2.08333333	3.31588133	4.66321244	3.17460317	1.60714286

contig_3287	1.09717868	1.62074554	2.03045685	1.80623974	2.05371248
contig_3290	2.60631001	2.42261104	3.63128492	3.30033003	3.26241135
contig_3291	2.35042735	4.2462845	1.10375276	2.76008493	1.91082803
contig_3292	1.20689655	1.66666667	1.95065978	1.836969	1.61012076
contig_3294	1.20056497	1.90677966	2.54237288	2.18926554	0.64148254
contig_3297	1.49625935	1.3732834	2.10918114	0.38265306	2.69905533
contig_3299	5.18518519	4.16666667	4.46428571	3.57142857	4.46428571
contig_329	0.90725806	2.09471767	0.88383838	1.49253731	2.08333333
contig_3302	1.14285714	1.87192118	1.33715377	0.95890411	1.19047619
contig_3303	1.8735363	3.10442145	3.5046729	0.80721747	2.73843248
contig_3305	1.31578947	3.92798691	3.76432079	4.3902439	2.16666667
contig_3306	4.97291974	6.50887574	6.75542406	5.24737631	6.28090999
contig_3307	1.13268608	2.76422764	5.20325203	3.1372549	2.17391304
contig_3309	1.2802276	2.64317181	5.49132948	3.85756677	3.13837375
contig_330	1.41843972	3.23488045	2.31155779	1.04821803	2.28873239
contig_3310	1.87110187	2.24948875	1.84162063	2.40174672	2.69151139
contig_3311	1.02315563	1.83091007	1.66935918	1.23855681	0.96930533
contig_3314	0.84911822	1.28783001	2.40963855	3.83048085	1.67088608
contig_3315	1.02647218	0.86673889	1.29659643	0.97297297	1.37589433
contig_3316	4.58135861	2.57648953	4.12698413	2.31839258	4.61783439
contig_331	1.16528672	1.65453342	2.04734485	1.40018067	1.78225494
contig_3321	2.50626566	0.28248588	4.26065163	2.78481013	1.25313283
contig_3327	0.71976967	1.05820106	0.86330935	1.39088729	0.8260447
contig_3328	0.64143682	1.07913669	1.1778563	1.09689214	0.97146327
contig_3330	1.0139417	2.05128205	2.12294043	1.84478372	1.36406397
contig_3331	2.5	2.98165138	1.82232346	0.45558087	1.59453303
contig_3333	1.43112701	2.29988053	2.11687537	1.67264038	1.23697917
contig_3335	2.77777778	3.81679389	4.34782609	3.56234097	2.82051282
contig_3337	0.94191523	1.93615908	1.55440415	1.82291667	0.98958333
contig_3343	3.47985348	4.76190476	5.61594203	2.40740741	3.66300366
contig_3345	1.8115942	2.53623188	2.15053763	1.43884892	0.88967972
contig_3348	3.16856781	3.56234097	3.00546448	0.82079343	1.44927536
contig_3349	0.90681676	1.37844612	1.12570356	0.46904315	1.07339716
contig_3350	3.97236615	14.5929339	2.05761317	6.21212121	6.96969697
contig_3353	2.98034242	6.56469089	7.47782003	2.42192479	1.07731305
contig_3355	3.67965368	4.76190476	4.54545455	3.03030303	2.2181146
contig_3356	2.97619048	3.37301587	3.90070922	2.38095238	2.97619048
contig_3357	3.73563218	5.17241379	4.02298851	3.16091954	1.47928994
contig_3358	0.48023642	1.36327185	1.56956637	1.09101349	1.57819225
contig_335	1.25	2.28782288	2.9134533	0.88573959	1.20481928
contig_3362	2.92207792	2.20338983	4.62519936	6.67779633	5.77557756
contig_3365	0.93177036	1.70454545	1.02194169	0.52151239	0.45126354
contig_3366	2.5	2.68714012	3.64683301	3.2967033	0.78431373
contig_3370	0.49916805	4.25170068	1.42180095	2.08333333	2.84280936
contig_3371	2.83687943	5.33333333	5.3030303	1.28676471	2.03703704
contig_3372	0.89020772	1.64365549	2.10180624	1.44927536	0.95363367
contig_3374	1.66944908	3.10880829	4.04040404	1.3400335	0.83752094
contig_3375	0.99337748	1.4356709	1.54525386	1.21614151	0.82827167
contig_3376	2.36220472	2.88713911	4.19947507	1.31233596	0.52631579

contig_3378	3.18627451	3.93374741	0.65897858	0.52910053	0.53097345
contig_3379	0.94339623	3.66056572	1.55296728	0.66592675	1.88888889
contig_3380	1.33868809	1.75202156	1.70709793	1.12676056	1.12917796
contig_3381	0.46554935	0.58969584	0.77591558	0.99440646	0.37243948
contig_3384	2.37467018	1.96483971	1.57958688	1.56548301	1.40081338
contig_3387	2.05643233	2.5456292	2.06830207	0.96200096	1.92400192
contig_3390	11.6438356	11.4155251	10.6854839	10.5022831	10.5022831
contig_3391	4.10958904	4.10958904	5.02283105	5.25114155	2.73972603
contig_3392	0.22148394	1.21883657	1.10741971	0.66445183	1.38427464
contig_3393	4.17457306	4.33925049	4.09982175	3.3530572	2.20440882
contig_3394	0.57518488	1.26893164	1.18609407	0.94146541	0.86513995
contig_3397	1.64814105	2.16014898	2.71880819	1.87597707	1.32992327
contig_3399	0.72544643	1.06809079	1.56075808	0.66964286	0.90039392
contig_339	2.72277228	2.35081374	2.44115083	1.44175317	1.29749768
contig_3400	1.8327606	3.32187858	2.06185567	1.48911798	3.31588133
contig_3401	0.78125	1.92798412	2.27670753	0.42529062	2.07887496
contig_3402	1.06221548	2.87859825	1.36986301	1.62297129	2.36794171
contig_3403	2.11640212	3.0411449	4.14673046	1.67597765	2.56410256
contig_3406	0.9557945	1.55409444	1.55316607	1.07655502	1.25523013
contig_3407	0.73163594	1.62098438	1.43400644	1.4645577	1.64030463
contig_3408	0.37093239	1.28075497	0.82616759	0.55809234	0.62426185
contig_3409	3.6437247	5.18867925	4.35606061	5.08130081	4.69083156
contig_340	0.85825028	1.28062361	1.30121816	0.75728891	2.41020794
contig_3412	2.10084034	1.96353436	3.81791483	1.26582278	0.75471698
contig_3415	3.22580645	3.65591398	2.15053763	3.22580645	1.50537634
contig_3417	3.72960373	4.1958042	4.1958042	3.26340326	3.26340326
contig_3418	0.6204757	1.15485564	0.82815735	1.34453782	0.68965517
contig_3419	1.5819209	2.30880231	1.85962807	1.62241888	1.2
contig_341	0.79033008	1.70786517	1.08843537	0.63291139	1.46341463
contig_3420	2.31213873	4.43159923	3.08285164	1.37254902	1.73410405
contig_3421	1.38067061	2.30566535	1.38431114	0.46854083	0.92165899
contig_3425	0.79656863	1.16421569	1.04166667	1.40056022	0.55147059
contig_3430	0.65978199	1.60688666	1.14777618	1.63996949	1.48853826
contig_3431	3.34128878	4.04761905	4.52380952	1.54525386	2.17391304
contig_3433	2.16802168	2.17714003	2.51382604	3.55750487	2.7080256
contig_3436	1.25786164	2.64227642	2.71966527	1.88679245	1.25786164
contig_343	1.89655172	1.99161426	0.21459227	1.59151194	2.89017341
contig_3441	0.81300813	2.24043716	1.91256831	1.40625	1.03199174
contig_3443	1.80383315	2.37556561	1.97674419	1.90369541	3.14685315
contig_3444	1.52817574	2.26628895	2.62681159	1.24069479	1.3986014
contig_3445	1.69082126	2.8277635	3.38164251	5.56900726	3.38164251
contig_3446	2.25225225	1.32547865	1.39275766	0.57202288	0.43731778
contig_3448	0.7797271	2.87565816	3.27933623	1.17029863	0.78201369
contig_3451	1.52284264	3.46715328	2.35294118	1.48148148	1.1627907
contig_3452	2.35602094	3.91304348	2.7027027	0.77821012	2.35131397
contig_3453	2.01196302	2.54742547	1.44110276	1.3681592	1.3601741
contig_3454	2.61780105	5.38194444	3.82513661	1.99275362	2.89115646
contig_3458	1.49812734	1.67785235	3.50877193	0.73800738	1.94931774
contig_345	0.75471698	1.41414141	2.04563336	3.32294912	0.39447732

contig_3460	2.25641026	5.32241556	3.14136126	3.03983229	4.14937759
contig_3462	0.91991342	2.05627706	1.94805195	0.54112554	1.13636364
contig_3463	2.96803653	5.25114155	2.51141553	3.40136054	2.49433107
contig_3465	1.11287758	3.76458112	1.05988341	1.11464968	0.58386412
contig_3466	1.10993658	2.18408736	1.89161554	2.43000528	1.68150346
contig_3467	2.10970464	1.15778137	0.726979	0.57692308	0.86160474
contig_3468	2.86738351	2.7607362	2.73556231	2.42424242	3.63636364
contig_3469	3.17460317	4.16666667	3.76984127	3.37301587	2.18253968
contig_346	1.96078431	2.97029703	4.76190476	1.70940171	1.07334526
contig_3470	3.15315315	3.15315315	3.15315315	3.3557047	2.92792793
contig_3471	1.67504188	2.34505863	1.92629816	0.58626466	2.94930876
contig_3475	1.02040816	1.2145749	3.23679727	4.08163265	1.01010101
contig_3477	2.89699571	3.45821326	2.35042735	2.20385675	1.8387553
contig_3478	1.02880658	1.52838428	1.51515152	1.51515152	1.2987013
contig_3479	2.08695652	2.94117647	3.98550725	1.28440367	2.60416667
contig_347	2.36220472	0.79681275	2.5527192	1.12570356	0.52631579
contig_3482	4.95238095	2.8340081	3.42465753	5.52486188	3.44827586
contig_3483	1.38135175	2.2178413	2.54403131	2.02869866	1.86548846
contig_3484	1.70454545	4.95238095	2.96684119	0.55147059	1.53256705
contig_3486	3.65591398	3.41880342	3.3970276	2.32067511	1.89873418
contig_3488	3.72807018	2.99401198	3.94265233	2.41545894	3.09423347
contig_348	1.00250627	2.95909487	1.85783522	1.36876006	0.58616647
contig_3491	7.76942356	7.26817043	8.02005013	7.26817043	7.26817043
contig_3496	7.21868365	7.21868365	8.86075949	9.55414013	7.90513834
contig_3497	2.09205021	4.02298851	1.46842878	2.13675214	1.62601626
contig_3498	1.56494523	1.95977308	1.65889062	1.2966805	1.19418484
contig_3499	2.17864924	4.35729847	6.36942675	1.52505447	1.30718954
contig_349	4.43548387	2.20264317	1.02639296	1.00286533	1.88976378
contig_34	2.18253968	3.50877193	4.87993803	2.91970803	1.74587779
contig_3501	1.67427702	2.38457636	2.73300631	1.56803237	2.02942669
contig_3504	4.22535211	4.5045045	4.87329435	4.81695568	2.43055556
contig_3505	1.50753769	1.86219739	2.36486486	1.69204738	1.68067227
contig_3507	0.83102493	1.47737765	1.56971376	0.46168052	1.51300236
contig_3508	1.89003436	3.19361277	1.2987013	0.50505051	0.17730496
contig_3509	2.42825607	3.16384181	4.23280423	2.36051502	3.6971831
contig_3510	0.54466231	1.24257158	1.08932462	0.54466231	0.48754063
contig_3512	1.88679245	4.81481481	4.37317784	2.93637847	3.72250423
contig_3513	0.97336066	1.12704918	1.38461538	0.56352459	0.5629478
contig_3514	3.10559006	2.69151139	4.9689441	2.89855072	2.05761317
contig_3517	0.68627451	1.03042198	1.32352941	0.6372549	0.88235294
contig_3518	2.2675737	2.42537313	5.29344074	0.64724919	4.05040504
contig_3519	2.03606748	5.34550196	2.39257813	6.56167979	1.03211009
contig_3520	1.8018018	2.36024845	2.24382947	1.94174757	0.7230658
contig_3522	0.95505618	1.92153723	1.02928128	0.7523511	0.9082218
contig_3523	0.67264574	2.82101167	1.39275766	2.22531293	0.36231884
contig_3528	1.9138756	1.69875425	2.28777845	1.32890365	0.74441687
contig_352	1.14702815	1.84797768	1.24352332	1.36876006	0.64784822
contig_3530	1.96882691	1.48963731	1.15900773	0.73313783	2.57731959
contig_3531	2.05761317	1.52671756	2.28816589	1.93617784	1.33333333

contig_3532	1.02564103	1.81112549	1.00628931	1.46750524	0.99206349
contig_3534	0.92681368	0.89988751	0.63734863	1.43678161	0.88008801
contig_3537	1.52542373	1.73333333	2.43664717	2.32815965	0.38095238
contig_3538	0.83959346	1.52477764	0.98345999	3.85964912	0.39840637
contig_3539	2.12150434	4.87804878	6.25641026	2.12014134	3.16573557
contig_3541	5.72390572	9.95145631	9.94035785	7.40740741	6.73400673
contig_3543	1.79573513	1.30134201	0.81366965	1.35674381	2.08695652
contig_3544	2.36666667	2.61690262	2.18609865	2.45614035	1.96078431
contig_3546	0.6795609	1.35734795	0.65155069	1.7699115	0.87719298
contig_3547	6.66666667	7.54716981	7.83289817	8.21806347	6.14035088
contig_3548	2.02492212	1.53985507	1.63934426	1.25136017	1.11731844
contig_354	2.17948718	1.65778567	1.81097906	0.76190476	1.86431901
contig_3553	1.38888889	3.68663594	2.40549828	1.26984127	1.63230241
contig_3556	1.52284264	2.88888889	1.60771704	1.03159252	0.91973244
contig_3558	1.5015015	5.27777778	3.9817975	0.78037904	3.72670807
contig_355	1.25	1.88356164	2.56991686		1.82186235
contig_3560	1.4014014	2.99539171	4.62107209	2.61044177	0.76142132
contig_3562	1.97530864	4.36590437	3.10136157	2.76647681	2.0431328
contig_3564	0.79954312	1.09170306	1.61711007	1.44	0.88832487
contig_3565	0.75503356	0.72992701	0.9771987	0.31948882	0
contig_3567	1.86092067	1.55823919	2.48376003	2.19560878	1.25247198
contig_3568	3.52941176	3.35595777	2.20088035	1.52173913	1.16807268
contig_356	0.84230229	1.41530296	1.99880668	1.55172414	0.86355786
contig_3570	2.43266725	1.83486239	2.15439856	1.52603232	1.60349854
contig_3571	2.02456024	1.98757764	1.44404332	1.22476001	0.52287582
contig_3572	1.78970917	3.32409972	3.60685302	0.20920502	0.23041475
contig_3574	1.14320096	3.35120643	2.2685469	1.4449127	1.68674699
contig_3576	1.18998769	1.29403307	0.64474533	1.04986877	0.63463282
contig_3578	1.39327024	9.90813648	4.42577031	1.54875044	1.61443495
contig_3579	3.15656566	1.76245211	2.40384615	1.16618076	1.21765601
contig_357	0.92336103	3.48977136	0.96153846	0.93240093	0.1529052
contig_3580	1.53683095	1.93615908	2.2845953	0.82530949	1.55279503
contig_3582	1.6025641	2.94117647	2.27560051	0.98765432	0.2283105
contig_3584	1.51975684	4.11081323	2.17831814	2.37529691	1.75859313
contig_3585	0.75566751	1.70940171	1.54291225	0.85714286	0.42372881
contig_3586	1.0644589	1.71969046	4.04916847	1.84182015	2.95532646
contig_3587	1.18675828	2.16169477	1.77935943	1.01083032	1.44927536
contig_358	1.8651363	1.66666667	3.23508267	0.85271318	1.31004367
contig_3590	0.95541401	1.49082569	1.08385625	1.50295223	0.36764706
contig_3591	1.64251208	1.63487738	1.74216028	1.34715026	1.34907251
contig_3594	1.25786164	1.65975104	2.28270413	1.81378476	1.05421687
contig_3595	1.61904762	3.20232897	2.73972603	1.71543895	0.55555556
contig_3596	0.8097166	3.24574961	1.02476516	4.96551724	0.37807183
contig_3597	1.04076323	3.01274623	1.99004975	2.84090909	1.05485232
contig_3599	1.23762376	1.43312102	1.90346703	1.38792505	2.11267606
contig_35	3.76470588	2.97225892	1.74757282	1.01744186	0
contig_3601	1.70387779	3.42316096	2.63004091	2.66798419	2.52525253
contig_3602	0.90909091	2.07792208	2.65780731	2.78011794	1.8404908
contig_3605	1.03286385	1.83006536	2.78503046	1.54559505	1.89483657

contig_3607	0.98436595	1.13161132	1.01260591	1.3871889	1.48966843
contig_3608	1.30285152	0.81621165	1.2254902	1.12721417	1.28939828
contig_3609	1.71886937	1.56128025	1.55590131	1.67548501	0.88987764
contig_3610	0.9355162	1.79104478	0.93992644	1.43385754	1.55595452
contig_3611	1.062417	3.85964912	2.25763612	0.83217753	1.875
contig_3612	1.33542812	3.79581152	1.13438045	3.97897898	1.25195618
contig_3613	1.40703518	1.72354361	1.73592846	1.91111111	1.86294079
contig_3614	1.34275618	1.94444444	1.49461244	1.29820542	1.40203295
contig_3616	1.65165165	2.97619048	6.83701657	6.66666667	2.66222962
contig_3617	0.87509944	1.12408128	1.85701021	1.47909968	2.78154681
contig_3619	0.71428571	5.14619883	2.44173141	2.38331678	2.18446602
contig_361	1.54569892	2.05761317	1.61857847	1.05471325	5.00556174
contig_3623	0.94503855	1.05820106	1.67753961	0.75801749	2.46516613
contig_3626	1.08695652	4.5751634	2.23182145	1.43988481	2.80528053
contig_3628	1.67264038	1.78468624	1.83867141	0.54644809	1.00401606
contig_3629	1.03092784	2.23048327	3.87878788	1.34730539	2.39410681
contig_3633	2.39130435	1.70244264	1.01596517	1.90217391	0.33898305
contig_3634	1.29789864	4.3946932	2.05338809	1.22484689	1.18525022
contig_3635	1.38888889	4.21313507	2.83783784	0.45941807	1.44927536
contig_3636	2.54110613	3.01724138	4.72069237	0.57915058	2.55720054
contig_3637	3.8961039	1.79487179	1.94902549	0.54644809	1.25348189
contig_3639	1.72839506	7.46527778	2.80898876	6.04026846	1.03383459
contig_3640	1.78710179	2.03784571	2.09090909	1.34099617	0.67873303
contig_3641	2.62597587	3.38424678	3.5	2.00761509	1.9406057
contig_3642	2.17917676	3.04878049	1.80952381	3.61648444	2.27908836
contig_3643	1.07286544	1.66892197	1.84453228	0.86477987	1.32978723
contig_3648	1.41110066	1.79948586	2.64279625	2.49017038	1.95121951
contig_364	1.57035176	4.07185629	2.4173028	0.9596929	1.70212766
contig_3650	3.54267311	1.6798419	1.7288444	2.79503106	1.33111481
contig_3654	2.07509881	2.0979021	2.40046838	0.77586207	2.83140283
contig_3655	2.13068182	2.82542886	2.01409869	2.60521042	1.2195122
contig_3658	0.80321285	1.15176152	1.46975692	2.07156309	1.46804836
contig_3659	2.23004695	2.44072524	2.18068536	3.5161744	2.25988701
contig_3660	1.07411386	1.92743764	0.95367847	1.40646976	0.41109969
contig_3662	1.40056022	1.38004246	2.20548682	1.02432778	1.35135135
contig_3663	1.8321513	2.09324453	1.31186643	0.90909091	1.03092784
contig_3664	2.23765432	2.33144297	0.88255261	2.35383447	1.24879923
contig_3671	2.21975583	1.67696381	1.5161503	1.3064133	1.99063232
contig_3673	2	0.78299776	4.33369447	2.44648318	6.54490107
contig_3674	1.89205955	2.24772836	1.85960019	2.055993	0.91292135
contig_3677	2.84697509	3.99201597	2.72063703	3.59281437	4.46735395
contig_3678	3.08370044	3.66598778	5.01285347	3.85964912	1.74978128
contig_3679	2.01257862	2.72727273	2.21354167	0.45841519	2.76094276
contig_3680	1.76297747	4.37710438	3.61904762	1.62059104	1.80952381
contig_3685	1.32774284	2.39752514	2.08779443	0.51428571	1.66570936
contig_3686	1.08191654	2.38751148	1.38568129	0.84985836	
contig_3687	1.76515733	2.46189918	1.62443145	1.291364	1.84229919
contig_3689	2.18463707	4.70175439	2.72259014	1.89274448	2.66666667
contig_3691	1.25	0.6302521	0.33112583	0.68259386	2.14285714

contig_3695	0.25210084	1.32090132	1.08010801	1.58730159	1.61073826
contig_3696	1.71379606	3.22580645	2.48500428	0.60085837	0.85689803
contig_3699	1.22044241	1.83639399	1.61616162	0.71620412	1.30568356
contig_36	0.63191153	1.4	1.84818482	0.83333333	1.00401606
contig_3701	1.2295082	1.27055306	1.66723375	1.20627262	0.93713393
contig_3702	2.19675263	3.96518375	2.34234234	0.94339623	1.57657658
contig_3704	1.77252585	1.46290491	1.87553282	1.062417	1.87165775
contig_3705	1.47757256	1.52905199	1.26582278	1.08754758	1.26782884
contig_3706	2.67062315	2.57171118	3.06627102	2.1760633	3.06627102
contig_3707	2.66666667	2.88713911	1.13935145	3.23974082	1.11731844
contig_370	2.25102319	3.26530612	3.34014997	3.3220339	4.27350427
contig_3713	1.74418605	3.01700494	1.45038168	2.192639	0.29732408
contig_3716	0.73277968	2.0609319	1.70316302	1.02189781	0.91911765
contig_3717	1.44927536	4.29141717	1.56521739	0.58207218	2.46478873
contig_371	1.33101852	2.08719852	1.55826558	1.78010471	1.63090129
contig_3723	2.51374705	1.61054173	6.01092896	4.47427293	1.79127726
contig_3724	0.63694268	1.9278607	1.87134503	0.84889643	1.82975338
contig_3725	2.11480363	2.11608222	1.34310134	0.71428571	2.61845387
contig_3726	2.44530245	2.00258398	3.10283688	0.81566069	2.7554535
contig_3727	1.10911271	1.58902131	1.47536146	1.47928994	2.49671485
contig_3728	1.88087774	4.47761194	3.97877984	1.7167382	2.42825607
contig_3729	1.26436782	1.31482834	1.6159105	1.21580547	2.29826353
contig_3730	16.0215054	17.1597633	18.9762797	18.8967136	19.1037736
contig_3731	2.48175182	7.99220273	4.56769984	5.04201681	1.39784946
contig_3734	0.95062864	1.03626943	0.67463968	1.51515152	1.09505037
contig_3735	1.27028934	2.68292683	2.09895052	5.43735225	3.36538462
contig_3738	2.77153558	2.79232112	2.86576169	2.63951735	2.49810749
contig_3739	1.37540453	1.937046	1.94174757	1.23565755	0.40518639
contig_373	1.81508792	1.23372173	1.901566	2.94342508	2.08333333
contig_3740	0.93795094	2.79206466	2.75913497	2.25035162	0.79470199
contig_3741	1.65945166	3.98860399	2.74170274	1.51515152	3.46320346
contig_3742	1.93089431	3.57873211	3.64741641	1.11448835	2.1194605
contig_3743	1.38297872	2.64150943	4.11985019	0.64279155	0.64279155
contig_3744	1.99637024	1.6938899	3.02480339	1.74672489	1.65425972
contig_3746	1.99733688	1.70715693	2.82152231	0.79365079	0.39946738
contig_3747	0.81497133	1.58172232	1.34502924	1.79468772	1.0031679
contig_3748	2.44755245	3.78250591	3.67734282	0.57971014	0.99502488
contig_374	4.14141414	2.67326733	2.08439248	3.99556049	0.92678406
contig_3750	1.26984127	2.75080906	3.61445783	0.47961631	1.06100796
contig_3752	0.51847051	1.48305085	1.67832168	1.09289617	1.54867257
contig_3753	4.32852386	4.43686007	5.69948187	4.54042082	4.08878505
contig_3756	1.56128025	2.69749518	2.11864407	1.95227766	1.72320948
contig_3759	1.45833333	2.8	1.53110048	0.73710074	2.22222222
contig_375	1.72786177	1.12211221	1.33555927	1.14660115	1.32052821
contig_3760	2.18579235	4.11255411	4.70588235	0.96153846	1.42231947
contig_3761	1.29081245	1.59939071	1.67046317	0.83586626	0.69767442
contig_3762	1.33333333	2.74599542	1.8018018	0.55955236	1.12359551
contig_3764	1.5576324	1.25786164	1.57728707	1.14566285	0.47923323
contig_3767	1.20025268	2.29007634	3.34788937	2.64705882	1.517067

contig_3768	0.61871616	3.36538462	2.86576169	0.31007752	0.83586626
contig_3769	2.34192037	1.38248848	2.44755245	2.56410256	2.25988701
contig_376	1.63043478	2.95275591	2.37370663	1.02739726	2.1760633
contig_3771	1.34680135	2.05673759	2.38095238	2.03665988	1.01146325
contig_3774	1.6336056	1.57004831	3.51966874	0.8045977	1.04166667
contig_3775	2.18068536	6.25	6.11111111	1.84162063	2.10727969
contig_3776	1.54135338	1.37795276	2.27686703	1.35236664	2.05479452
contig_3779	1.07526882	6.48967552	2.82051282	4.6728972	2.19675263
contig_3782	0.86555107	2.26876091	1.32871173	2.85316632	4.6854083
contig_3783	1.52505447	2.3174971	2.06297503	1.76211454	0.9771987
contig_3784	5.70902394	5.92592593	7.34767025	2.89855072	3.03030303
contig_3785	8.42105263	4.84048405	11.3354037	5.28789659	5.23321957
contig_3786	1.34112792	2.87990196	1.44329897	0.3502627	1.44181256
contig_3787	1.37566138	2.50284414	4.11985019	3.77073906	2.07156309
contig_3789	1.21580547	0.73800738	1.2962963	1.79806362	3.36700337
contig_3793	1.03329506	3.22003578	3.1124498	1.66666667	1.05691057
contig_3795	1.64968462	2.22772277	2.41652021	1.10497238	2.49355116
contig_3796	1.02040816	1.64574616	2.14776632	0.5946935	1.74193548
contig_3797	0.69444444	8.53658537	8.62470862	12.1621622	10.4938272
contig_37	1.35135135	3.01204819	1.37382502	1.11662531	2.17054264
contig_3801	1.32890365	5.67901235	3.4904014	3.25077399	2.11640212
contig_3802	1.0521701	1.62529551	1.33385641	1.17967332	1.16113744
contig_3804	2.94985251	4.98442368	9.52380952	5.2154195	1.61030596
contig_3806	1.79104478	1.85758514	1.08359133	1.86335404	0.89445438
contig_3807	1.97823937	6.02409639	2.42326333	1.99203187	1.3986014
contig_3809	8.16522574	2.64187867	6.68604651	5.47588005	8.58283433
contig_380	0.78328982	1.41003948	1.57924422	2.24806202	1.36518771
contig_3810	2.75	2.71604938	4.10958904	3.68852459	2.25921522
contig_3812	2.51121076	1.09589041	2.42587601	2.18579235	0.69444444
contig_3813	1.66666667	4.29447853	2.94715447	1.96721311	2.10409745
contig_3814	2.90556901	3.59771055	1.8268467	1.05691057	1.39917695
contig_3815	0.62893082	2.96242775	4.10526316	1.29496403	1.34803922
contig_3816	1.98821797	2.70935961	1.13717129	1.56482861	1.3740458
contig_3818	0.62305296	1.85479597	1.51515152	2.35042735	1.46031746
contig_3821	1.78571429	3.21543408	3.48639456	2.29885057	1.44557823
contig_3822	1.45772595	4.5675413	3.30417881	1.06899903	4.17881438
contig_3824	1.59624413	6.1281337	3.89363723	2.53968254	2.90237467
contig_3825	2.08623088	4.46194226	4.35356201	4.21792619	4.62809917
**contig_3826**	**1.47225368**	**2.72572402**	**3.25476992**	**2.32717317**	**0.68681319**
contig_3827	1.75438596	2.33918129	4.00410678	1.96078431	1.4619883
contig_3828	3.40960072	4.66237942	4.30107527	4.01987353	2.60557053
contig_3831	0.89605735	4.42338073	1.36116152	2.76752768	0.70921986
contig_3833	1.47058824	5.6372549	1.96078431	2.45098039	4.16666667
contig_3834	0.88797814	2.41286863	1.83798502	0.58139535	1.13168724
contig_3840	1.02040816	1.20724346	0.93856655	1.02040816	2.9455081
contig_3843	1.60525356	2.29885057	1.23997082	1.7581629	0.77134986
contig_3845	1.3916501	2.18037661	3.80622837	2.98507463	0.57034221
contig_3846	0.5841548	1.33779264	1.37495355	1.32058288	1.02115244
contig_3847	11.882716	10.1851852	8.95061728	6.79012346	9.41358025

contig_3848	1.96078431	3.47003155	3.39506173	1.8907563	1.12044818
contig_3849	1.19165839	2.01511335	2.406639	2.07920792	2.80082988
contig_3850	1.5060241	0.70052539	1.42169729	0.75956763	1.37062937
contig_3852	0.80847505	1.30992196	1.02159953	2.09542231	0.94760312
contig_3853	1.00334448	6.07375271	4.01034929	4.27350427	0.48543689
contig_3857	0.85261876	1.23281176	1.89189189	1.71218916	0.63948841
contig_3859	8.07017544	6.57596372	6.83333333	6.2103929	6.41025641
contig_385	3.07291667	3.34261838	1.48988214	5.88235294	1.80685358
contig_3860	4.28954424	2.05540661	3.11614731	4.31309904	3.01075269
contig_3861	0.70621469	1.48514851	1.85185185	0.73529412	2.09150327
contig_3863	2.12290503	2.75913497	2.47933884	1.10011001	2.83975659
contig_3864	2.39043825	6.84410646	3.92902408	0.92879257	1.88172043
contig_3866	1.34575569	1.60493827	1.87617261	1.2195122	1.78571429
contig_3867	0.2688172	3.72907154	3.09278351	1.84757506	1.02040816
contig_3868	2.6826484	2.63819095	1.85897436	1.32501949	1.44927536
contig_3869	1.69971671	3.69357045	3.77002828	1.69491525	2.18778487
contig_3870	1.30662021	2.02492212	1.3224822	1.66333999	0.79365079
contig_3872	3.99449036	7.49646393	4.66830467	0.78125	4.11764706
contig_3874	0.77378243	2.2131888	2.58525853	1.50891632	2.25108225
contig_3877	0.71326676	1.05519481	2.07833733	0.65359477	2.61506276
contig_387	0.41341295	1.26786538	1.20789779	1.19512756	0.55884287
contig_3880	1.36778116	5.63583815	2.04081633	1.07913669	1.9047619
contig_3881	0.83160083	1.77809388	2.49520154	1.87165775	1.28012048
contig_3882	1.53061224	1.91919192	1.97530864	1.7699115	2.5
contig_3884	0.7569386	0.85261876	0.4803074	1.15646259	0.2451982
contig_3886	4.62724936	2.41423126	3.24074074	1.95618153	1.87018702
contig_3887	4.39189189	1.1627907	2.80193237	3.1884058	0.97943193
contig_388	0.50697085	1.01265823	1.9047619	1.10132159	1.96078431
contig_3891	5.01319261	3.41419587	2.96684119	2.72251309	5.50807217
contig_3892	0.92592593	1.69927909	3.13152401	1.60142349	1.92307692
contig_3894	3.23624595	2.76243094	3.13075506	2.31481481	1.56494523
contig_3896	1.44258511	1.97101449	1.97101449	0.80691643	3.37209302
contig_3897	1.1328976	2.9318037	0.86695565	0.76897359	0.80952381
contig_3899	1.20793788	3.68550369	1.31147541	1.25	0.99502488
contig_3901	0.35149385	2.28310502	2.53718285	1.48883375	1.3546798
contig_3902	1.74165457	1.67641326	1.64126612	0.85971082	0.74257426
contig_3903	0.29498525	2.28571429	2.46153846	1.04633782	1.47420147
contig_3904	3.46820809	3.10734463	4.33526012	2.48538012	1.24378109
contig_3905	1.82954038	3.89326334	2.63888889	2.2397892	1.84615385
contig_3906	6.22222222	6.44444444	4	6.22222222	6.11111111
contig_3908	8.81057269	7.63582966	7.03296703	7.1278826	4.55212922
contig_3910	0.75136612	2.55144033	3.2996633	1.37187653	1.4084507
contig_3911	1.04683196	1.06286061	0.74560095	0.60734892	0.90600227
contig_3913	1.48898154	1.74059517	1.36904762	1.79964007	1.82046453
contig_3914	1.89982729	2.68398268	3.0224525	1.23152709	2.73381295
contig_3916	1.13871636	1.86335404	1.23152709	1.44927536	0.84680523
contig_3917	1.47446024	3.05810398	2.47435124	3.07414105	2.5974026
contig_391	0.9699321	2.39390642	1.82767624	1.47954743	1.54639175
contig_3920	1.07737512	1.75953079	1.27326151	0.58823529	1.27201566

contig_3922	0.78796562	1.007109	1.99764982	2.5994486	1.14285714
contig_3923	1.1627907	1.10029623	2.37789203	1.84094256	1.06022053
contig_3924	0.58191585	1.78041543	2.44008715	0.85357846	1.57170923
contig_3925	0.72845002	1.49779736	1.79518564	1.55292195	1.70487948
contig_3926	2.48618785	2.68199234	3.16455696	2.05479452	3.77777778
contig_3927	1.25523013	2.04081633	1.66975881	0.58788948	0.80395795
contig_3928	1.31578947	2.18181818	1.38888889	1.92926045	2.28658537
contig_3930	6.69144981	6.22065728	6.15563298	9.61923848	3.67847411
contig_3931	2.014295	2.40415854	2.83687943	0.99800399	3.03239145
contig_3933	0.90361446	2.29057592	2.5177026	1.41557128	1.37254902
contig_3936	2.30991338	2.51082251	2.06318504	1.03225806	0.5988024
contig_3937	9.85915493	3.75586854	7.51173709	1.6509434	1.91256831
contig_3938	0.38374324	1.16928447	0.57561486	1.12938866	0.7504363
contig_3939	1.68539326	4.3956044	3.07867731	2.72676683	0.22408964
contig_3940	0.97451274	1.72802404	1.78571429	3.00429185	0.92748735
contig_3942	1.81229773	2.8277635	2.31660232	2.81803543	1.8018018
contig_3944	1.96656834	1.77238806	1.58450704	1.50976909	1.55844156
contig_3947	0.66225166	3.14569536	2.37306843	2.20440882	0.55187638
contig_3948	2.42744063	7.51173709	1.55002349	3.67917586	2.4600246
contig_3949	2.50135943	3.54609929	4.18705818	2.09125475	1.25272331
contig_394	0.93708166	1.27175368	1.6064257	1.00874243	0.52287582
contig_3950	1.42450142	2.809482	2.34741784	1.27919911	3.44585091
contig_3951	4.8	7.9491256	4.26540284	4.51010886	5.93220339
contig_3955	1.46842878	3.38235294	1.81818182	1.91458027	4.01606426
contig_3956	10.7981221	11.2676056	11.2676056	11.0328638	10.7981221
contig_3957	0.53304904	1.44578313	2.12765957	1.27226463	1.93133047
contig_3959	1.79968701	2.6128266	2.05714286	1.82841069	1.77865613
contig_395	0.6662965	1.55309033	1.49647887	0.85106383	0.42296073
contig_3961	0.80941869	1.40740741	1.64271047	1.28976784	1.55316607
contig_3963	1.36131594	1.63432074	1.54004107	1.28799588	2.3628692
contig_3967	1.67748918	4.50038139	3.37301587	0.62464509	3.64328439
contig_3968	2.34636872	1.94931774	2.79034691	3.03983229	3.37349398
contig_396	0.96200096	2.32007576	1.18371212	2.10589651	0.91390091
contig_3970	1.25739645	2.74414851	2.82152231	2.38095238	0.63965885
contig_3971	2.80448718	2.30414747	1.8115942	2.40740741	2.04402516
contig_3974	4.54545455	4.0802213	3.91246684	3.37972167	3.14606742
contig_3977	1.27388535	2.13017751	1.45268944	2.1601686	1.06382979
contig_3978	0.82987552	1.68010753	1.5742642	1.20603015	0.96192385
contig_3979	1.75019889	3.44827586	2.00772201	0.8709422	1.86915888
contig_397	0.62893082	3.60934183	3.38345865	3.9348711	1.30718954
contig_3980	1.50214592	3.23054332	2.72	2.23820943	1.08695652
contig_3981	2.38751148	2.48962656	4.68319559	2.38751148	1.83150183
contig_3982	2.20646178	3.22061192	4.15537489	1.46596859	2.20646178
contig_3984	3.07328605	2.21453287	2.33406273	2.17864924	2.17086835
contig_3985	1.32669983	2.3023023	2.06270627	1.73267327	1.15511551
contig_3986	1.72732881	4.27860697	2.39043825	1.40306122	1.01975781
contig_3989	1.19047619	2.66565118	3.53460972	0.92807425	1.26682502
contig_3991	3.88751034	4.2218543	10.4395604	4.42773601	7.01754386
contig_3992	3.55555556	5.78512397	5.55555556	3.03370787	3

contig_3993	2.08514335	1.85512367	2.78019114	3.84087791	2.60869565
contig_3996	1.96687371	1.96687371	1.6563147	1.44927536	1.5560166
contig_3997	1.2855831	1.46923783	1.5610652	1.2855831	1.46923783
contig_3999	1.40306122	1.02367242	1.27713921	1.14869177	1.11111111
contig_399	0.78895464	3.06748466	3.26472676	0.95486111	0.63965885
contig_4000	4.94417863	2.15311005	3.43383585	1.75879397	1.52
contig_4001	0.97222222	2.5	2.36111111	1.78041543	2.64072272
contig_4002	2.07677785	1.60493827	1.14613181	1.56521739	2.47557003
contig_4004	1.11856823	2.05128205	2.25127088	0.75244545	1.16194626
contig_4007	1.89526185	2.97979798	3.84231537	2.58302583	2.45398773
contig_4009	0.87301587	1.5	1.47470125	1.2345679	1.55807365
contig_400	1.45833333	1.59744409	2.54160363	1.01010101	1.88547486
contig_4011	1.95635816	2.43710692	1.81818182	1.2608353	2.89855072
contig_4013	1.91693291	3.38635198	3.0816641	2.3006135	2.87179487
contig_4014	1.81211254	2.89655172	2.7100271	2.80941534	1.66204986
contig_4015	5.58292282	6.5681445	6.07553366	6.89655172	7.06075534
contig_4017	1.36147039	2.62467192	2.45566166	1.9781719	3.35025381
contig_4018	2.47678019	1.75298805	2.53333333	3.21969697	1.1820331
contig_4020	2.13178295	2.93398533	2.52182347	2.22868217	3.44530577
contig_4026	3.50241546	3.61581921	3.98550725	2.88018433	2.30769231
contig_4027	1.42646335	0.98328417	1.75141243	2.36220472	1.03346457
contig_4028	1.61812298	3.54077253	2.71493213	3.22580645	2.59179266
contig_4030	1.08225108	2.83950617	3.21350763	1.5394913	1.24391563
contig_4031	1.57894737	5.14705882	4.01891253	3.44827586	3.125
contig_4042	2.47747748	2.47747748	2.7027027	3.15315315	3.15315315
contig_4050	0.91093117	2.05042948	1.1676397	2.65363128	0.81967213
contig_4051	1.20772947	1.78759201	4.30906389	2.39867659	2.58899676
contig_4055	1.00182149	1.94292653	0.68292683	0.56915196	1.43205363
contig_4057	2.1236727	3.7037037	3.70634355	2.73405137	2.36907731
contig_405	1.96463654	3.71794872	2.6384083	0.9765625	0.72036673
contig_4066	3.79213483	6.14035088	5.12232416	4.76190476	2.18579235
contig_4069	3.12204352	2.74621212	4.8245614	1.93072118	1.85409109
contig_406	1.56092649	2.38095238	2.56797583	1.76233635	1.38312586
contig_4070	1.48975791	2.15897939	2.60869565	1.97238659	0.63694268
contig_4079	1.51234568	1.18534483	2.13581599	1.40728477	1.68818272
contig_407	1.22302158	1.96548418	1.64694966	3.09917355	1.38089758
contig_4081	4.66237942	4.99306519	6.41025641	3.85164051	2.22222222
contig_4082	1.41843972	2.82186949	2.53251198	0.9122807	1.53027279
contig_4083	1.42755175	2.45901639	1.48544266	0.41493776	1.91470844
contig_4084	4.15686275	1.86781609	1.40734949	0.52521008	1.62074554
contig_4086	1.26482213	3.73303167	1.55400155	1.44782826	1.83823529
contig_4087	1.16888351	1.08827086	1.21261116	1.65255945	0.87045571
contig_4088	1.98807157	1.82348651	2.47933884	2.70906949	1.38169257
contig_408	2.0278834	4.34782609	4.53333333	1.30890052	0.94117647
contig_4090	1.78197065	4.71380471	3.98843931	2.84552846	2.90205562
contig_4096	0.75821398	1.52905199	1.93602694	1.17056856	1.08342362
contig_4099	1.95721438	1.16110305	1.45687646	1.04477612	1.16071429
contig_409	1.404741	2.0216267	2.14384509	1.5971606	4.03005464
contig_4106	1.15384615	1.61532682	1.98205717	1.44324174	1.24740125

contig_4107	5.19877676	7.58082497	7.91100124	1.00143062	1.70068027
contig_4108	2.57400257	2.15909091	3.35648148	1.23726346	0.71301248
contig_410	1.94915254	1.53751538	4.26179604	0.55762082	1.62200282
contig_4112	5.875	6.75	6.3670412	6.4516129	5.01253133
contig_4118	0.51314945	0.94517958	1.53846154	2.91627469	1.77595628
contig_4119	1.16189001	2.15568862	1.19331742	1.53508772	1.10987791
contig_411	1.68067227	2.94372294	3.17460317	2.1515435	2.89719626
contig_4120	2.6481715	1.55555556	2.64150943	1.66279969	1.82291667
contig_4121	3.2288699	3.07167235	2.22652469	3.64341085	1.4571949
contig_4122	6.35964912	8.16777042	2.23642173	5.97014925	5.05725191
contig_4123	1.25842697	1.84269663	2.455838	2.87719298	2.734375
contig_4125	1.49019608	3.17460317	2.52659574	0.60168472	0.83632019
contig_4126	3.2586558	1.88067445	2.20941402	2.20318237	1.36736554
contig_4129	1.2254902	2.93159609	2.200489	1.2755102	1.2254902
contig_412	3.93120393	5.96421471	4.41176471	4.08163265	3.97350993
contig_4132	1.51515152	2.91897333	2.6429342	2.04962244	0.9751773
contig_4133	0.94057289	1.7976032	1.48576145	0.26041667	0.73055436
contig_4134	3.0876494	2.06022187	1.43849206	1.48731409	1.91637631
contig_4135	17.9487179	21.0743802	16.1616162	18.1311018	18.9488243
**contig_4136**	**7.75067751**	**9.1954023**	**7.3371284**	**4.5995671**	**4.63659148**
contig_4138	1.066961	1.39808683	1.39808683	2.59419395	1.33958473
contig_4140	1.98159943	2.95790671	2.78388278	1.17216117	1.94585448
contig_4141	1.87449063	2.18140069	3.42298289	1.25786164	1.95121951
contig_4143	0.63719634	1.02564103	0.86455331	1.31782946	1.01091791
contig_4144	2.84810127	9.46666667	5.52380952	5.21376434	10.3009259
contig_4145	1.31578947	2.33785822	2.18905473	1.8916595	0.5242464
contig_4147	2.268431	2	1.9989195	2.38095238	2.31246475
contig_4149	2.4691358	2.64705882	2.5540275	1.87590188	1.68650794
contig_4150	1.41325536	2.0797227	1.4084507	1.4893617	1.44404332
contig_4152	1.51515152	3.3446712	3.88809862	3.69836695	3.18664643
contig_4153	2.03252033	2.57997936	2.24489796	2.92164675	1.13722517
contig_4154	1.07108082	1.75953079	2.96735905	2.26155359	1.38476756
contig_4155	1.31313131	1.23076923	1.45833333	0.51813472	1.11111111
contig_4156	2.14460784	1.73680695	2.23651961	2.20588235	2.57352941
contig_4158	7.94573643	11.5973742	11.7428925	8.74751491	6.23781676
contig_4159	10.6382979	8.04733728	6.85579196	0.48449612	4.53781513
contig_415	1.70648464	2.58136925	1.23734533	1.68539326	4.43622921
contig_4161	2.18068536	3.49726776	3.57142857	4.42600277	2.53968254
contig_4162	3.80952381	2.19028063	3.75653828	3.63247863	1.74825175
contig_4163	7.74550484	6.33737185	7.49665328	5.61403509	5.47686497
contig_4167	1.32450331	3.52941176	4.89130435	2.11898941	4.28134557
contig_4168	6.71077505	9.69827586	9.87884436	7.8125	5.66037736
contig_4169	2.195734	5.96685083	2.25763612	4.22429907	4.87804878
contig_4170	4.69348659	6.49188514	4.31293882	4.00890869	1.12359551
contig_4171	6.16142945	7.88022065	9.6875	9.05797101	6.55324512
contig_4172	16.4658635	16.064257	14.6118721	15.7684631	14.6118721
contig_417	1.06347624	1.28008193	1.144641	0.81967213	1.84831103
contig_418	1.88298588	2.41610738	2.15053763	1.25847047	1.41474311
contig_419	1.34744947	1.51874703	1.27659574	0.67534973	1.97585071

contig_420	1.09289617	1.70682731	2.56410256	2.6119403	0.60679612
contig_422	1.59074476	2.09689082	3.31905782	1.8813314	2.03389831
contig_424	1.60984848	3.94736842	2.1780303	0.92764378	4.7752809
contig_426	0.6147541	2.92353823	2.39398085	6.36237898	1.82348651
contig_427	2.19435737	2.34933606	2.2289767	2.12765957	1.21703854
contig_429	1.51515152	2.57611241	2.09386282	1.06837607	3.07692308
contig_430	1.37254902	1.11856823	1.37254902	1.47058824	0.98039216
contig_432	2.31481481	1.80932498	1.88679245	1.06707317	1.14503817
contig_433	3.05676856	4.00457666	3.5639413	3.4965035	4.48916409
contig_435	0.38095238	1.56862745	4.23572744	0.49627792	2.59067358
contig_436	0.91074681	3.29218107	2.22222222	2.75689223	1.79640719
contig_441	1.73913043	4.18353576	3.68663594	2.57936508	1.70454545
contig_442	2.60273973	4.32220039	4.15512465	2.5290499	1.38203356
contig_443	3.39403974	2.71021543	3.32464146	3.08571429	2.17391304
contig_445	1.9253911	2.61437908	1.96801968	1.80505415	1.46341463
contig_446	1.26436782	1.20341615	1.6318205	1.11169931	1.31396957
contig_447	1.30718954	1.32231405	5.26315789	2.33160622	0.96153846
contig_44	1.50669643	0.99299065	1.45611964	1.11856823	0.82987552
contig_453	1.46443515	2.87921348	3.58649789	1.83598531	2.71777003
contig_459	3.61604208	4.33656958	3.31457161	6.21193666	1.00864553
contig_460	1.54536391	1.69888694	1.97086547	1.73992674	0.99052541
contig_461	1.77035913	3.22061192	2.21193416	0.81607031	3.20675105
contig_463	0.57175529	1.08548168	0.79225352	1.01387407	2.41850683
contig_464	0.40650407	1.94444444	1.90995907		4.65116279
contig_465	0.54794521	1.86335404	3.05343511	1.47601476	1.83066362
contig_466	2.11221122	2.68907563	2.10637177	3.74787053	1.32669983
contig_467	0.51480051	4.27807487	2.67379679	1.36054422	2.85204991
contig_468	0.97012029	1.1577424	1.54679041	1.19876257	1.11835974
contig_46	1.10387772	1.04031209	2.1611002	0.33388982	1.85283219
contig_470	1.23329908	1.8764154	1.29516019	1.40350877	0.80753701
contig_474	1.68067227	2.27531286	2.75449102	2.58094791	3.97812034
contig_475	0.80200501	1.62381597	2.50719277	1.34958642	1.58259149
contig_476	2.18687873	2.39567233	1.58045977	1.10826939	1.30434783
contig_478	1.53452685	2.21987315	1.02249489	1.26742712	3.86100386
contig_484	1.26413839	2.00400802	1.68690958	2.71903323	2.74656679
contig_485	0.68438003	0.92592593	1.14239086	1.81629476	1.34200895
contig_486	0.86021505	1.57303371	1.35795763	1.64399093	0.74677529
contig_488	0.99337748	1.98675497	1.76600442	1.33037694	1.88087774
contig_489	1.06382979	2.18340611	0.39772727	1.41223941	2.18340611
contig_491	2.77777778	1.58730159	2.87828947	1.3836478	2.5297619
contig_497	1.97758734	7.27699531	2.73081925	4.10396717	8.88192268
contig_498	1.1846002	1.00755668	1.28458498	1.59521436	1.68650794
contig_500	1.9895288	3.18181818	1.53846154	2.90215589	1.07526882
contig_502	1.06951872	1.79372197	1.88425303	2.9082774	0.62977958
contig_506	1.75182482	3.43053173	1.0989011	1.45922747	2.8436019
contig_507	0.83046964	1.37106184	0.85929108	1.6	0.67074949
contig_508	1.28913444	2.21606648	2.12569316	1.84162063	1.8134715
contig_509	1.46520147	3.003003	1.88909202	0.55079559	1.82926829
contig_50	2.06435944	1.38121547	1.10344828	2.12765957	1.0270775

contig_510	0.9557945	5.59610706	2.91375291	3.84615385	3.48837209
contig_512	1.13452188	4.10742496	6.96202532	2.52365931	6.55737705
contig_515	1.10864745	1.35440181	2.42825607	2.42825607	1.937046
contig_516	2.62195122	2.14646465	2.20500596	2.13567839	0.67873303
contig_518	5.50807217	3.7037037	5.95463138	3.96475771	6.0434372
contig_519	1.24444444	2.20507166	2.48070562	1.32953466	0.47003525
contig_520	1.79640719	3.22580645	2.24550898	1.65036675	0.74850299
contig_521	1.10076207	1.59651669	1.6722408	0.98585512	2.63277349
contig_523	1.69836957	1.79324895	1.42700329	1.04166667	1.64705882
contig_524	0.87365591	1.48749155	1.88172043	0.73924731	0.81135903
contig_525	0.33783784	3.47923681	1.98675497	0.95882685	2.29540918
contig_526	3.9039039	6.30630631	7.73333333	3.3033033	4.5045045
contig_527	2.12264151	1.76678445	1.66073547	1.1778563	0.95808383
contig_529	2.22222222	1.68804862	2.08197788	4.52830189	1.35542169
**contig_52**	**1.48054146**	**1.40774258**	**1.59521436**	**2.80373832**	**0.9199632**
contig_532	0.97592713	1.03297576	1.48975791	1.10441767	0.73051948
contig_534	1.62314749	2.15053763	2.37741456	1.60183066	4.27728614
contig_535	1.20481928	2.61865794	2.3255814	1.32743363	0
contig_536	1.7452655	2.10425634	1.63618864	2.81803543	1.26445087
contig_539	1.84630739	1.32908028	1.58127767	1.9874477	2.1927588
contig_540	5.40540541	2.05128205	2.40963855	0.6116208	0.8097166
contig_541	2.52764613	8.83838384	5.96330275	7.960199	6.42201835
contig_546	4.84330484	2.28571429	1.29375951	5.54355652	2.37681159
contig_548	1.20391272	3.79403794	2.18373494	3.36726703	2.7479092
contig_549	2.21300138	1.56918688	1.51828847	1.93236715	1.74757282
contig_550	1.10011001	1.17474302	1.71526587	1.61458333	0.86517664
contig_552	3.44352617	3.11111111	1.89189189	2.79569892	2.69541779
contig_558	2.31213873	1.8327606	2.4691358	1.2295082	0.52631579
contig_560	0.71754729	1.53733529	1.41532455	0.81068193	0.79012346
contig_562	1.10565111	1.80567498	2.66579974	0.58536585	2.06896552
contig_565	4.21686747	3.27978581	2.22222222	4.55153949	2.54350736
contig_566	2.40137221	2.0661157	2.00320513	0.57603687	1.07681263
contig_56	1.0996119	2.12264151	2.68817204	1.09717868	0.49627792
contig_571	1.20036934	2.03139428	2.11786372	1.20036934	1.48342059
contig_573	0.87863811	0.94444444	1.43964563	0.63657407	0.90039392
contig_574	2.39520958	3.27754533	3.65934797	3.33333333	3.39285714
contig_575	1.90615836	1.8079096	2.02360877	1.02564103	1.70674942
contig_576	8.54092527	8.22649573	7.90598291	1.70940171	9.56284153
contig_578	1.09431996	2.73618998	2.25840336	3.28358209	0.66006601
contig_57	1.2345679	1.55440415	1.01851852	1.28939828	1.59313725
contig_580	1.31386861	2.18253968	1.34486071	0.96339114	0.87032202
**contig_583**	**1.12079701**	**1.41129032**	**0.96899225**	**1.27659574**	**1.05820106**
contig_584	1.41975309	1.55979203	1.43884892	1.85185185	0.60200669
contig_587	1.127089	1.5478424	0.85339835	2.13414634	1.8922853
contig_589	0.67713143	2.08558073	2.7155465	1.10132159	0.65052403
contig_590	1.3562387	1.19965724	1.26841244	1.89847297	1.00686499
contig_591	7.77777778	8.88223553	7.98353909	8.36431227	8.74493927
contig_592	6.26654898	2.35988201	1.55709343	1.15340254	2.2397892
contig_593	1.41430948	2.36148955	2.55427842	3.16455696	1.33000831

contig_594	0.65520066	3.84615385	2.20125786	1.53203343	1.43781452
contig_595	1.04790419	3.84087791	1.53374233	1.32113821	2.40334378
contig_596	1.44807613	3.9049236	3.00925926	1.75925926	2.03421174
contig_59	2.38095238	1.92307692	1.81229773	0.52724077	1.45833333
contig_603	0.70156503	1.6	2.87356322	1.3536379	0.72689512
contig_607	2.29276896	2.07138305	1.96523054	1.68808264	2.00878845
contig_609	1.87573271	0.72815534	2.77108434	2.16010165	2.51256281
contig_60	9.43738657	7.37913486	7.68431983	4.45859873	6.61538462
contig_614	1.40280561	2.0800832	1.35375846	1.72328902	0.66815145
contig_617	2.35640648	3.92156863	1.71052632	5.74712644	2.65486726
contig_619	1.92427064	1.68067227	1.56081808	0.87431694	0.55126792
contig_621	2.3890785	2.15893431	1.46471372	1.33982948	1.66964818
contig_622	0.75093867	1.24716553	1.61711007	1.21012101	1.85873606
contig_623	1.91804708	5.61056106	2.04498978	2.7027027	2.71929825
contig_625	1.31670132	3.19001387	3.03657695	1.93637621	2.20994475
contig_626	1.6007533	3.24675325	3.01075269	0.96463023	1.82795699
contig_628	1.17647059	2.5527192	2.27995758	1.19402985	0.88495575
contig_62	1.25984252	2.13049268	1.12359551	1.06685633	1.42276423
contig_631	1.13052415	1.64670659	3.13283208	1.85597624	1.78173719
contig_633	2.04342273	2.69461078	2.35003092	1.41025641	1.68612192
contig_634	2.00913242	3.24976787	5.14767932	3.95480226	6.51801029
contig_63	1.35708227	3.44456405	4.21052632	0.51413882	1.21065375
contig_640	1.36752137	3.03030303	2.22222222	2.16346154	1.52733119
contig_642	0.85744909	2.51428571	1.18137596	2.2675737	1.2345679
contig_643	2.38095238	1.27610209	2.34146341	0.6718925	3.28849028
contig_644	1.77304965	2.63157895	3.03975058	1.3559322	1.73913043
contig_645	1.64992826	2.61635913	2.4238061	1.69971671	3.12648034
contig_648	1.32496513	2.49221184	1.33779264	1.7873101	1.21168924
contig_651	7.10144928	7.64525994	8.84615385	5.64202335	6.88172043
contig_653	1.404741	1.36752137	1.15942029	1.55239327	0.96755834
contig_654	2.12560386	2.16450216	3.12757202	0.71237756	3.07503075
contig_655	1.33020344	3.23299889	2.04402516	1.43198091	1.10062893
contig_656	4.37997725	3.40314136	3.75426621	3.20150659	2.78725825
contig_657	1.05337079	1.75438596	1.96491228	1.61064426	1.20226308
contig_658	1.58469945	2.76679842	2.37854251	1.84615385	1.04683196
contig_661	2.01028518	1.87441425	1.12359551	1.54738878	1.55221072
contig_662	1.85185185	2.25618632	1.76340926	3.5035035	0.92059839
contig_663	11.4155251	12.5570776	11.6438356	10.9589041	11.6438356
contig_664	4.10447761	11.4355231	9.89010989	9.09090909	0.39840637
contig_668	1.3559322	1.81818182	2.40963855	1.87793427	3.32975295
contig_669	7.98122066	6.79380215	6.87960688	7.12530713	7.50853242
contig_66	2.10896309	2.05592105	1.61527166	4.88888889	3.51758794
contig_673	1.42671855	1.19047619	1.99004975	1.29701686	0.513573
contig_674	1.24671916	1.58045977	1.44356955	1.19435396	0.76124567
contig_675	5.32646048	6.32183908	1.34357006	2.23367698	3.69747899
contig_678	1.41129032	1.72143975	1.24564026	1.74291939	1.16906475
contig_679	4.12979351	2.59319287	2.26244344	2.71565495	0.68493151
contig_67	0.67146283	1.2195122	1.33460439	2.35294118	0.8912656
contig_683	1.80064309	1.7756255	2.81045752	0.9155646	1.64149705

contig_684	0.44682752	1.35135135	3.6019536	1.48883375	1.92076831
contig_686	1.05307498	1.33547009	1.19341564	1.60427807	0.81231295
contig_688	1.50684932	1.88679245	2.43183493	2.29555237	0.7497657
contig_690	0.55147059	3.27669903	2.83687943	2.43902439	1.55440415
contig_693	1.3029316	2.33918129	3.2586558	1.76240209	1.64502165
contig_694	2.0431328	1.87361067	1.70454545	0.81081081	0.999001
contig_696	1.40032949	2.16819974	1.57170923	1.02960103	0.19736842
contig_697	2.02663578	2.42424242	1.43329658	1.01265823	1.83626626
contig_699	2.55668114	3.61519608	3.13555234	4.15879017	4.02262728
contig_700	0.82758621	1.70827858	1.6509434	1.17493473	2.07407407
contig_701	0.85382514	2.11360634	1.37659784	0.52157421	0.63460254
contig_702	6.00600601	4.98442368	4.21348315	5.26315789	7.62711864
contig_703	1.01960784	1.26582278	1.80392157	2.22513089	1.44508671
contig_706	0.952705	1.16092874	1.5246934	2.46562352	0.90521832
contig_708	2.63991552	1.79324895	1.16156283	1.26582278	0.94936709
contig_70	2.17391304	2.10573477	1.67029775	1.12570356	1.09126984
contig_710	1.67548501	2.88713911	3.85078219	1.17222723	1.67865707
contig_712	1.75438596	3.784219	2.36794171	2.10387903	1.63398693
contig_713	2.34702431	3.125	2.99145299	1.37299771	0.35555556
contig_714	3.29861111	1.74825175	4.86111111	0.52539405	2.25694444
contig_716	2.03217612	2.63991552	2.83806344	2.02360877	1.4198783
contig_717	2.61941448	1.38248848	4.76190476	1.83150183	2.05128205
contig_718	1.57303371	1.83066362	2.39700375	2.5477707	0.73855244
contig_719	1.30952381	0.93276331	1.13122172	1.6399287	1.81253237
contig_71	1.57894737	2.98672566	1.75763182	3.12837109	3.75
contig_720	8.0474934	8.16864295	8.56389987	8.03689065	9.63060686
contig_722	3.61445783	2.08333333	1.02171137	1.45348837	1.91780822
contig_723	1.19731801	1.06434446	1.96769457	1.41659439	0.89186176
contig_724	0.99778271	2.13675214	1.1789925	1.23647604	0.34246575
contig_726	2.02702703	3.6036036	4.05405405	1.63599182	3.88888889
contig_728	4.82758621		3.28638498	1.25984252	1.88679245
contig_72	0.75901328	0.95238095	0.92710185	1.96447793	0.83030618
contig_731	0.49342105	1.28068303	2.09828824	0.5789909	1.51260504
contig_733	2.20673635	1.99600798	2.11981567	0.12870013	2.06185567
contig_734	1.76531672	1.79063361	2.41984271	1.39808683	0.96793708
contig_737	0.32733224	3.32278481	1.52380952	1.18694362	1.43329658
contig_739	1.17171717	1.78571429	1.29449838	1.04427736	1.0105093
contig_73	1.46341463	1.33333333	2.30591852	1.03244838	0.49261084
contig_740	3.77358491	3.4562212	4.53149002	4.688701	3.40314136
contig_743	0.84245998	2.13004484	1.58127767	0.94501718	1.62950257
contig_747	3.12109863	2.2675737	4.24469413	1.58730159	1.02040816
contig_748	2.90697674	4.63917526	2.92397661	2.39410681	1.85873606
contig_749	1.51228733	2.38095238	1.97758734	1.63522013	0.49751244
contig_751	1.33982948	2.8526149	2.28187919	1.29954516	1.93976519
contig_754	1.77083333	2.95857988	1.50706436	0.46511628	0.92807425
contig_758	1.28205128	2.3255814	2.5974026	1.11642743	0.97493036
contig_760	1.05633803	2.06489676	1.27826942	1.71717172	0.62176166
contig_761	1.6004415	1.80392157	1.45268944	0.3968254	1.69291339
contig_766	0.71620412	2.59391771	1.89018902	3.6101083	2.16021602

contig_768	2.79232112	3.15656566	1.88087774	3.02297461	3.40136054
contig_76	0.78515346	0.66889632	1.79352581	1.48975791	2.17640321
contig_771	0.66183423	1.76721511	1.27591707	1.06312292	1.42326733
contig_772	0.87489064	0.73956683	2.11640212	0.84470435	1.35958744
contig_773	1.92906036	1.54471545	1.20048019	1.33600534	0.98870056
contig_774	1.90039318	1.50053591	2.03012443	1.76817289	0.78125
contig_775	2.68500327	3.22427691	2.40360541	0.59037239	2.95320309
contig_777	1.11964171	2.16147489	1.52091255	2.16162288	1.69983416
contig_778	1.73160173	2.87769784	3.46320346	2.16450216	2.74170274
contig_779	2.03327172	1.89982729	2.89532294	1.90895742	3.78006873
contig_77	2.12234707	1.5392509	1.50048403	4.67836257	2.00716846
contig_781	9.23369824	9.43223443	10.7941037	8.63137816	8.31702544
contig_786	1.80750113	1.6293279	2.05949657	2.18423552	1.08374384
contig_787	1.57126824	2.44648318	0.80645161	1.43149284	0.21978022
contig_789	1.44804089	1.77935943	2.19409283	1.26689189	3.77668309
contig_791	1.62703379	2.38500852	1.19453925	1.03092784	2.13414634
contig_793	1.38888889	3.08457711	2.36928105	1.96078431	1.72413793
contig_794	0.8423586	2.71317829	1.44404332	0.54466231	2.09424084
contig_797	1.00647017	2.26795464	1.71489818	2.17306946	0.64599483
contig_799	2.26308345	2.40601504	2.31023102	1.45131086	0.54794521
contig_800	1.41242938	2.08037825	2.75908479	1.89139719	1.41110066
contig_801	1.70250896	1.98618307	1.70250896	1.17011701	2.89855072
contig_802	0.64620355	2.70618557	1.42450142	1.47420147	1.28676471
contig_803	1.55172414	1.05868119	1.11843992	0.75757576	1.13553114
contig_806	0.60551014	1.9404572	0.90161761	1.65710644	1.43859649
contig_807	1.37880987	1.82481752	2.67201069	0.78563412	2.46533128
contig_810	1.64512338	3.18148952	2.10997442	1.93298969	1.26984127
contig_811	1.2145749	2.42261104	2.15343203	0.81855389	0.72595281
contig_816	0.91883614	1.23339658	1.76419966	0.40983607	1.09034268
contig_821	1.64609053	0.96385542	1.6644474	0.31779661	0.71174377
contig_822	0.94594595	1.98019802	6.06060606	2.86576169	1.26262626
contig_824	1.75120773	2.73259597	2.87461774	3.95256917	1.83823529
contig_831	2.92207792	0.67567568	2.56410256	1.35658915	1.04166667
contig_834	1.83121019	2.71234832	2.3468576	1.43426295	1.48261759
contig_836	13.836478	14.1509434	17.6100629	8.25688073	6.16883117
contig_837	0.56116723	1.63098879	0.88987764	0.9489917	0.48154093
contig_838	3.9039039	2.01005025	2.08900999	1.45322434	1.71277997
contig_839	1.39616056	1.33517495	1.13168724	1.47563487	1.69491525
contig_83	0.87102178	1.65456013	1.60720026	0.88	1.82704019
contig_840	5.26544822	8.09399478	6.22280244	6.33977216	7.06467662
contig_842	0.81424936	1.87040748	1.15273775	0.71428571	0.83256244
contig_843	1.57437568	3.29317269	2.61682243	1.52380952	2.09973753
contig_845	1.50564617	0.6116208	2.21914008	1.80055402	1.44300144
contig_849	3.11640697	2.39410681	2.53045923	3.75	0.63091483
contig_84	1.56128025	1.8735363	2.26385636	0.46666667	0.78534031
contig_850	0.93147039	2.56410256	1.69270833	2.28915663	0.6012024
contig_855	2.75229358	3.80952381	2.22222222	1.56599553	1.95035461
contig_856	2.17864924	2.52672498	1.41542817	0.60168472	1.20311394
contig_857	2.62329486	3.32434861	4.283054	2.60707635	1.67890871

contig_859	1.66852058	2.18631179	1.93050193	0.82219938	2.0696143
contig_85	1.07066381	0.97087379	0.65693431	0.36036036	1.22149837
contig_861	1.35908441	1.00574713	1.10565111	2.64026403	1.01522843
contig_862	1.62601626	1.92165558	1.25646711	2.01793722	0.78125
contig_863	2.52469813	4.24581006	4.15183867	2.09267564	3.64741641
contig_864	2.37918216	1.48478099	1.56361052	0.89820359	0.78534031
contig_867	0.58823529	1.80467091	1.31411615	1.92165558	1.01419878
contig_869	1.59574468	2.41545894	3.16301703	2.03252033	0.70778564
contig_870	1.40166448	2.14442013	2.35910878	0.60667341	1.26970228
contig_871	1.08108108	4.01529637	2.95652174	2.86738351	2.43055556
contig_872	0.60137457	2.12604404	1.5910899	2.47172183	0.89781958
contig_874	0.80536913	1.02908277	1.16331096	1.16435289	0.53691275
contig_876	1.02339181	1.63487738	2.99319728	0.98846787	0.55172414
contig_879	2.07522698	0.94674556	1.7452007	1.98198198	1.01483216
contig_87	0.57803468	2.21606648	1.51380232	2.30607966	1.37362637
contig_880	0.69735007	1.27388535	2.80970626	0.9383378	0.11587486
contig_882	1.66534496	2.5450031	2.28677379	0.63965885	0.64935065
contig_886	2.56410256	2.93171255	2.35651843	1.42056075	2.49839846
contig_887	1.2703252	1.11567821	0.65557237	1.27616131	0.99220411
contig_88	0.81521739	1.51057402	1.38067061	1.93861066	0.93312597
contig_890	1.58730159	2.11640212	2.3715415	1.85185185	1.71957672
contig_892	0.61946903	1.348504	1.29107981	1.38067061	0.9310987
contig_894	1.01010101		3.003003	0.61099796	2.13414634
contig_895	0.70257611	2.00597525	3.44036697	1.4721346	1.19131044
contig_896	1.52722444	3.37609723	3.32225914	1.66333999	2.85524568
contig_897	1.2145749	1.24172185	1.82322632	1.12057373	0.57471264
contig_898	1.51921358	3.12779267	1.57657658	1.25142207	1.70709793
contig_89	1.27388535	2.16572505	1.33704735	1.46163216	0.26490066
contig_8	1.23674912	3.03541315	1.40562249	1.50300601	2.16606498
contig_900	2.36111111	3.82165605	3.08641975	2.08333333	1.08932462
contig_902	1.10062893	2.16154721	1.96078431	0.78740157	1.4229249
contig_905	0.92961487	1.72642762	1.53027279	0.79840319	0.63613232
contig_908	2.53411306	1.65745856	1.2755102	2.88600289	4.11877395
contig_90	1.53846154	2.36220472	1.58045977	1.83585313	2.69607843
contig_910	1.79573513	4.66988728	1.55210643	0.57077626	1.55210643
contig_913	1.18089104	1.82403433	1.28962923	1.90735695	3.44827586
contig_914	1.72711572	1.84225676	1.55555556	0.43290043	1.0989011
contig_915	0.62111801	4.18250951	1.7037037	3.73831776	2.43902439
contig_918	0.89108911	1.26582278	2.22092344	1.46771037	2.02464789
contig_919	1.95775374	2.82051282	1.8826937	1.10957004	1.46750524
contig_925	0.57573074	1.5936255	1.01814962	0.61974325	1.46082337
contig_927	0.93984962	2.85423038	1.42711519	1.8018018	2.63513514
contig_928	1.4354067	1.38888889	1.54173312	1.46590185	2.14088398
contig_929	1.75097276	1.49253731	1.45833333	1.09780439	1.78759201
contig_92	1.22399021	2.48397436	1.19271814	2.01900238	2.19907407
contig_930	0.46413502	1.348504	1.63198694	1.31524866	1.06863954
contig_931	3.04726368	1.69491525	2.72776869	1.5241882	1.51679307
contig_932	1.96592398	4.25963489	5.11182109	2.53623188	4.41001192
contig_935	1.22426266	1.34255492	1.97835013	1.41757797	1.04733976

contig_936	1.80840665	2.98142717	3.22108346	2.52304706	1.36852395
contig_937	1.68336673	1.43072289	1.04166667	0.41666667	0.8
contig_938	1.17593849	1.62011173	0.91827365	1.01061142	1.38888889
contig_939	1.88679245	2.20883534	2.22222222	1.59151194	1.2345679
contig_93	1.9019019	4.02298851	4.25633668	1.22377622	1.5910899
contig_940	7.50341064	10.5263158	8.23529412	8.47457627	3.33333333
contig_941	1.53683095	4.10885806	1.96913252	1.36876006	0.79840319
contig_944	1.49625935	3.13293819	2.32751455	2.24438903	2.19966159
contig_945	1.71184023	1.42180095	2.95238095	3.16384181	2.81385281
contig_94	1.42180095	1.76908752	6.36363636	1.78890877	4.38596491
contig_950	4.36408978	2.95566502	3.1420765	0.71428571	1.4242116
contig_952	1.06280193	1.83574879	1.54004107	0.77071291	0.91827365
contig_953	1.44444444	1.75953079	1.66177908	1.96078431	1.47058824
contig_955	2.64227642	2.20868241	3.50877193	2.13675214	1.48601399
contig_956	1.55844156	1.91754554	3.48943985	1.61030596	1.34099617
contig_957	1.06288751	1.49965917	1.36518771	1.44628099	0.75459318
contig_958	0.77058565	1.17603822	1.2164902	0.84147666	0.54434838
contig_95	1.04408353	6.28019324	2.62675626	0.97765363	1.01910828
contig_960	1.39372822	1.86666667	3.76647834	2.3943662	0.62111801
contig_962	0.88888889	1.58878505	1.43705092	2.99019608	1.15456238
contig_965	1.0488616	0.80717489	0.98457499	0.58027079	1.14490161
contig_968	1.00069013	1.53297683	1.34343782	1.06227106	0.68941744
contig_96	5.22088353	7.07762557	9.61538462	3.57995227	5.77777778
contig_970	1.23809524	2.19047619	1.91605839	1.52380952	1.88087774
contig_971	5.05952381	5.9972106	3.09278351	1.08527132	1.54320988
contig_979	0.48923679	1.36452242	1.11922141	1.58835208	0.94517958
contig_97	2.77777778	2.85474391	2.24719101	3.41034103	0.58708415
contig_980	2.78044104	3.91621129	2.43589744	1.04562738	1.3368984
contig_981	6.66666667	4.49438202	3.96825397		2.4
contig_982	2.24948875	1.21827411	1.76322418	0.73260073	0.63041765
contig_983	1.41093474	2.53699789	1.83066362	2.01793722	1.43737166
contig_984	2.22222222	2.26130653	1.10083664	2.12841433	1.52259332
contig_985	1.0668164	1.03986135	2.90016732	1.71875	1.88679245
contig_986	1.68471721	2.27001195	2.27817746	1.56438026	1.558753
contig_990	3.63937138	1.97255575	1.78723404	3.22580645	1.51133501
contig_992	2.14067278	2.16718266	2.44648318	1.65118679	0.8255934
contig_994	2.86458333	2.4911032	2.2589053	0.97777778	1.43369176
contig_996	1.84162063	2.39410681	1.65745856	1.65745856	1.5610652
contig_997	1.26619552	1.53166421	1.10086284	1.37907837	0.90403338
contig_998	1.31694469	2.19885277	2.09424084	1.2195122	1.87265918
contig_9	1.20160214	1.24378109	2.81690141	2.11764706	1.55316607
contig_3661	2.10084034	2.38095238	4.47976879	1.30208333	3.22128852
average	2.27957455	2.65533604	2.57782365	1.96463193	1.86402546
