# Supplementary material for: Evolution of Transcriptomes in Early-Generation Hybrids of the Apomictic Ranunculus auricomus Complex (Ranunculaceae)
Source: Int J Mol Sci. 2022 Nov 10;23(22):13881. doi: 10.3390/ijms232213881 (PMC9697309; doi:10.3390/ijms232213881)
Supplement: Supplementary file 1 [file ijms-23-13881-s001.zip › Supplemental Table S4_Perl_summary.pdf]

Supplemental Table S4. Average dN/dS ratios of the longest ungapped ORF of each putative nuclear single copy gene estimated by the Nej-Gojobori algorithm as implemented in Bioperl.

contig	average dN	average dS	average dN/dS
contig_1002	0.0032	0.0027	1.03937008
contig_1005	0.009	0.0326	0.44600939
contig_1009	0.0045	0.0125	0.64444444
contig_101	0	0.0049	0.67114094
contig_1011	0.0016	0.0361	0.2516269
contig_1012	0.0025	0.0286	0.3238342
contig_1017	0	0.0204	0.32894737
contig_1018	0.0021	0.0194	0.41156463
contig_1020	0.004	0.0104	0.68627451
contig_1021	0.0021	0.0336	0.27752294
contig_1024	0	0.0196	0.33783784
contig_1025	0.0115	0.1313	0.15215853
contig_1028	0.0023	0.0276	0.32712766
contig_1029	0.0013	0.0094	0.58247423
contig_1030	0.0015	0.0512	0.1879085
contig_1031	0.0029	0.008	0.71666667
contig_1033	0.0195	0.0027	2.32283465
contig_1036	0.0032	0.0069	0.78106509
contig_1039	0	0.0119	0.45662101
contig_104	0.0014	0.0271	0.30727763
contig_1046	0.004	0.0089	0.74074074
contig_1047	0.0035	0.0234	0.40419162
contig_1049	0.0013	0.0139	0.47280335
contig_105	0.0086	0.2412	0.07404459
contig_1051	0.0056	0.0082	0.85714286
contig_1053	0.002	0.0162	0.45801527
contig_1055	0.0183	0.0358	0.61790393
contig_1059	0	0.0022	0.81967213
contig_1061	0.0033	0.0287	0.34366925
contig_1064	0.0021	0.0109	0.57894737
contig_1069	0.0072	0.0192	0.5890411
contig_107	0	0.0089	0.52910053
contig_1077	0.0063	0.0133	0.69957082
contig_1079	0.0027	0.0242	0.37134503
contig_1083	0.0026	0.0171	0.46494465
contig_1084	0.0018	0.0161	0.45210728
contig_1085	0.0017	0.011	0.55714286
contig_1086	0.0036	0.0135	0.5787234
contig_1087	0	0.0137	0.42194093
contig_1089	0.0019	0.0141	0.49377593
contig_1092	0.006	0.0231	0.48338369
contig_1093	0.0088	0.0244	0.54651163
contig_1094	0.0005	0.0102	0.51980198

contig_1095	0.0116	0.057	0.32238806
contig_1098	0.0012	0.0229	0.34042553
contig_1103	0.0011	0.0151	0.44223108
contig_1106	0.0066	0.0146	0.67479675
contig_1108	0.0018	0.0135	0.50212766
contig_1110	0.0009	0.0211	0.35048232
contig_1111	0.0014	0.0308	0.27941177
contig_1114	0.001	0.0117	0.50691244
contig_1119	0.0021	0.0351	0.26829268
contig_112	0.0018	0.0268	0.32065217
contig_1121	0	0.0158	0.3875969
contig_1124	0	0.0085	0.54054054
contig_1127	0.0048	0.0131	0.64069264
contig_113	0.0058	0.014	0.65833333
contig_1130	0.0038	0.0375	0.29052632
contig_1131	0.0026	0.0216	0.39873418
contig_1136	0.0022	0.0136	0.51694915
contig_1138	0.0008	0.0353	0.2384106
contig_1139	0	0.0149	0.40160643
contig_1140	0.0049	0.0245	0.43188406
contig_1141	0.0016	0.0148	0.46774194
contig_1142	0.0139	0.0233	0.71771772
contig_1143	0.0069	0.011	0.80476191
contig_1146	0.0014	0.0083	0.62295082
contig_1147	0.0009	0.0182	0.38652482
contig_1149	0	0.0139	0.41841004
contig_115	0.0014	0.0066	0.68674699
contig_1156	0.0008	0.0104	0.52941177
contig_116	0.0009	0.0019	0.91596639
contig_1161	0	0.0122	0.45045045
contig_1162	0.0007	0.0052	0.70394737
contig_1165	0.0012	0.0121	0.50678733
contig_1166	0.0012	0.0052	0.73684211
contig_1168	0.0019	0.0097	0.60406091
contig_1173	0.0011	0.0211	0.35691318
contig_1174	0.0022	0.0196	0.41216216
contig_1175	0.0027	0.0085	0.68648649
contig_118	0.0056	0.0157	0.60700389
contig_1183	0.0032	0.0056	0.84615385
contig_1187	0	0.0618	0.13927577
contig_1191	0.0042	0.0166	0.53383459
contig_1192	0.0031	0.0023	1.06504065
contig_1195	0	0.0037	0.72992701
contig_1197	0.0033	0.0309	0.32518337
contig_1199	0.0006	0.0123	0.47533632
contig_1200	0.0009	0.0266	0.29781421
contig_1201	0.0024	0.0136	0.52542373
contig_1207	0.0016	0.0096	0.59183674
contig_1208	0.0052	0.0082	0.83516484

contig_121	0.0148	0.0534	0.39116719
contig_1211	0.0078	0.0296	0.44949495
contig_1216	0.0015	0.0129	0.50218341
contig_1220	0.0007	0.0022	0.87704918
contig_1224	0.0046	0.044	0.27037037
contig_1228	0.0041	0.0242	0.4122807
contig_1229	0.0041	0.0562	0.21299094
contig_1232	0.0016	0.0153	0.45849802
contig_1234	0.0025	0.012	0.56818182
contig_1235	0.0013	0.0106	0.54854369
contig_1238	0.0003	0.0113	0.48356808
contig_1240	0.0023	0.0713	0.15129151
contig_1241	0.0008	0.0087	0.57754011
contig_1242	0.0066	0.0273	0.44504021
contig_1247	0.0119	0.1287	0.15789474
contig_1251	0	0.0037	0.72992701
contig_1253	0.0038	0.023	0.41818182
contig_1256	0.0056	0.0121	0.70588235
contig_1257	0	0.0075	0.57142857
contig_1259	0.0049	0.0051	0.98675497
contig_126	0	0.0144	0.40983607
contig_1261	0.0014	0.0106	0.55339806
contig_1263	0.0033	0.014	0.55416667
contig_1267	0.0027	0.0063	0.7791411
contig_127	0.0033	0.0049	0.89261745
contig_1274	0	0.0129	0.43668122
contig_1275	0	0.0026	0.79365079
contig_1278	0.0006	0.0114	0.4953271
contig_1282	0.0033	0.0226	0.40797546
contig_1286	0	0.0114	0.46728972
contig_129	0.0009	0.0359	0.23747277
contig_1294	0.0034	0.0315	0.32289157
contig_1300	0.0007	0.0426	0.20342205
contig_1302	0.0039	0.019	0.47931035
contig_1304	0.0014	0.0141	0.47302905
contig_1310	0.0008	0.0316	0.25961539
contig_1318	0.0026	0.0204	0.41447368
contig_132	0.0034	0.0132	0.57758621
contig_1320	0.0008	0.0377	0.22641509
contig_1324	0.0024	0.0284	0.32291667
contig_133	0.0085	0.0368	0.39529915
contig_1331	0.0009	0	1.09
contig_1333	0.0035	0.0253	0.38243626
contig_1342	0	0.0181	0.35587189
contig_1347	0.0141	0.0291	0.61636829
contig_1348	0.0024	0.0131	0.53679654
contig_135	0.0016	0.0172	0.42647059
contig_1352	0.0027	0.0205	0.41639344
contig_1356	0.0044	0.0075	0.82285714

contig_136	0.0037	0.0077	0.7740113
contig_1362	0.0024	0.0382	0.25726141
contig_1366	0.0023	0.0042	0.86619718
contig_1367	0.0065	0.0114	0.77102804
contig_1371	0.0018	0.0205	0.38688525
contig_1372	0.0013	0.0138	0.47478992
contig_1377	0	0.0048	0.67567568
contig_1378	0.0076	0.0548	0.27160494
contig_1383	0.0006	0.0116	0.49074074
contig_139	0.0029	0.0393	0.26166329
contig_1393	0.0011	0.0059	0.69811321
contig_1398	0.0024	0.0107	0.59903382
contig_1400	0.0009	0.0041	0.77304965
contig_1406	0.0014	0.0107	0.55072464
contig_1408	0.0025	0	1.25
contig_1409	0.0095	0.0384	0.40289256
contig_141	0.001	0.0171	0.40590406
contig_1413	0.0036	0.0182	0.4822695
contig_1416	0.0057	0.0112	0.74056604
contig_1418	0.0075	0.0385	0.36082474
contig_142	0.0021	0.0087	0.64705882
contig_1420	0.0076	0.0468	0.30985916
contig_1423	0.0095	0.0214	0.62101911
contig_1435	0.0206	0.0232	0.92168675
contig_1436	0.0017	0.0188	0.40625
contig_1441	0.0014	0.022	0.35625
contig_1448	0.0011	0.0127	0.48898678
contig_145	0.0043	0.0142	0.59090909
contig_1451	0.0051	0.0319	0.36038186
contig_1454	0.0029	0.0586	0.18804665
contig_1457	0.0038	0.0278	0.36507937
contig_1459	0	0.0164	0.37878788
contig_1460	0.0034	0.0108	0.64423077
contig_1464	0.0002	0.0141	0.42323652
contig_1465	0.0018	0.0137	0.4978903
contig_1468	0.0036	0.0066	0.81927711
contig_1469	0.0002	0.0103	0.50246305
contig_147	0.0021	0.0224	0.37345679
contig_1482	0	0.0235	0.29850746
contig_1483	0	0.0332	0.23148148
contig_1485	0	0.0156	0.390625
contig_1491	0.0013	0.0047	0.76870748
contig_1493	0.001	0.0097	0.55837564
contig_1495	0.0012	0.0122	0.50450451
contig_1496	0.0047	0	1.47
contig_15	0	0.0089	0.52910053
contig_1506	0.0033	0.0039	0.95683453
contig_151	0.0018	0.0226	0.36196319
contig_1510	0.0027	0.0158	0.49224806

contig_1511	0.0039	0.0076	0.78977273
contig_1513	0.0009	0.0111	0.51658768
contig_1519	0.0035	0.0148	0.54435484
contig_152	0.0025	0.0078	0.70224719
contig_1525	0.0009	0.022	0.340625
contig_1527	0.0063	0.0494	0.27441077
contig_1530	0.0037	0.0119	0.62557078
contig_1531	0.0081	0.0056	1.16025641
contig_1532	0.0045	0.0087	0.77540107
contig_1534	0.0014	0.0077	0.6440678
contig_1535	0.0008	0.026	0.3
contig_1537	0	0.0288	0.25773196
contig_1540	0.0049	0.0148	0.60080645
contig_1543	0.0016	0.0188	0.40277778
contig_1546	0	0.0233	0.3003003
contig_1548	0.0006	0.011	0.50476191
contig_1549	0.0039	0.0443	0.25598527
contig_1562	0.0012	0.0245	0.32463768
contig_1566	0.0019	0.0018	1.00847458
contig_1567	0.0022	0.0032	0.92424242
contig_1568	0	0.0258	0.27932961
contig_157	0.0012	0.0066	0.6746988
contig_1572	0.0017	0.0071	0.68421053
contig_1573	0.0028	0.031	0.31219512
contig_1576	0.0039	0.0189	0.48096886
contig_1580	0.0014	0.0158	0.44186047
contig_1581	0.0011	0.0052	0.73026316
contig_1584	0.0027	0.0122	0.57207207
contig_1585	0.001	0	1.1
contig_1587	0.0035	0.0173	0.4945055
contig_1589	0.0018	0.0424	0.22519084
contig_1591	0.0027	0.0072	0.73837209
contig_1599	0.0033	0.0135	0.56595745
contig_1600	0.0009	0.0167	0.4082397
contig_1606	0.004	0.014	0.58333333
contig_1610	0.0022	0.0154	0.48031496
contig_1611	0.0014	0.008	0.63333333
contig_1623	0.0026	0.0104	0.61764706
contig_1624	0.0013	0.0156	0.44140625
contig_1627	0.0028	0.0116	0.59259259
contig_1628	0.0145	0.0418	0.47297297
contig_163	0.0041	0.0143	0.58024691
contig_1630	0.0016	0.0078	0.65168539
contig_1631	0.0042	0.0049	0.95302013
contig_1636	0.0042	0.0159	0.54826255
contig_1640	0.0009	0.0118	0.5
contig_1642	0.0017	0.0204	0.38486842
contig_1644	0.0014	0.0087	0.60962567
contig_1647	0	0.0597	0.14347202

contig_1650	0.0075	0.0107	0.84541063
contig_1652	0.0044	0.017	0.53333333
contig_1654	0.0018	0.0201	0.39202658
contig_166	0.002	0.0099	0.60301508
contig_1662	0.0007	0	1.07
contig_1665	0.0016	0.0067	0.69461078
contig_1666	0.0022	0.011	0.58095238
contig_1667	0	0.0033	0.7518797
contig_1668	0.003	0.0233	0.39039039
contig_1669	0.0077	0.0116	0.81944444
contig_1670	0.0037	0.0173	0.5018315
contig_1674	0.0005	0.0092	0.546875
contig_1676	0.0046	0.009	0.76842105
contig_168	0.0017	0.0149	0.46987952
contig_1684	0.0037	0.0311	0.33333333
contig_1686	0	0.0097	0.50761421
contig_169	0.0017	0.0088	0.62234043
contig_1690	0.0028	0.0337	0.29290618
contig_1693	0	0.0071	0.58479532
contig_1695	0.012	0.0061	1.36645963
contig_1696	0	0.0086	0.53763441
contig_1697	0.0026	0.0111	0.5971564
contig_1698	0.0073	0.031	0.42195122
contig_170	0.0021	0.0118	0.55504587
contig_1701	0.0039	0.0051	0.9205298
contig_1709	0.0043	0.0236	0.42559524
contig_1713	0.0011	0.0203	0.36633663
contig_1715	0.0033	0.0386	0.27366255
contig_1718	0.001	0.013	0.47826087
contig_172	0.0021	0.0098	0.61111111
contig_1726	0	0.0082	0.54945055
contig_1727	0	0.0188	0.34722222
contig_1729	0.0023	0.0171	0.45387454
contig_1735	0.0004	0.012	0.47272727
contig_1737	0.0022	0.0177	0.44043321
contig_1739	0.0094	0.0171	0.71586716
contig_1741	0	0.0087	0.53475936
contig_1742	0.0008	0.0239	0.31858407
contig_1744	0	0.0131	0.43290043
contig_1745	0.0017	0.0283	0.30548303
contig_1746	0.0025	0.0151	0.49800797
contig_1747	0.0016	0.0438	0.21561338
contig_1752	0.0016	0.0174	0.42335766
contig_1753	0.0043	0.015	0.572
contig_1754	0.0044	0.0124	0.64285714
contig_1755	0	0.0111	0.47393365
contig_1757	0.0018	0.0145	0.48163265
contig_1758	0.0003	0.0067	0.61676647
contig_1760	0.0008	0.0141	0.44813278

contig_1761	0.0024	0.0116	0.57407407
contig_1762	0.0032	0.0067	0.79041916
contig_1763	0.0068	0.0082	0.92307692
contig_1764	0.0049	0.0102	0.73762376
contig_1766	0.0063	0.0127	0.71806167
contig_177	0.0013	0.0109	0.54066986
contig_1770	0.0104	0.0447	0.37294333
contig_1772	0.0034	0.0114	0.62616822
contig_1773	0	0.0536	0.1572327
contig_1774	0.0047	0.0208	0.47727273
contig_1775	0.0016	0.0257	0.32492997
contig_1778	0.0007	0.007	0.62941177
contig_178	0	0.0293	0.25445293
contig_1786	0.0024	0.0218	0.38993711
contig_1788	0	0.0292	0.25510204
contig_179	0	0.019	0.34482759
contig_1790	0.0028	0.0118	0.58715596
contig_1794	0.0029	0.0288	0.33247423
contig_1796	0.0021	0.0367	0.25910064
contig_180	0.0045	0.0113	0.68075117
contig_1801	0.0053	0.0185	0.53684211
contig_1803	0.0036	0.0083	0.7431694
contig_1807	0.0022	0.0302	0.30348259
contig_1808	0.001	0.0356	0.24122807
contig_1812	0.0015	0.0188	0.39930556
contig_1814	0.001	0.0056	0.70512821
contig_1821	0.0023	0.0196	0.41554054
contig_1824	0.002	0.0332	0.27777778
contig_1826	0.0024	0.0194	0.42176871
contig_1827	0.0004	0.0061	0.64596273
contig_183	0	0.004	0.71428571
contig_1830	0.001	0.0084	0.59782609
contig_184	0.0007	0.0136	0.45338983
contig_1841	0.0021	0.0047	0.82312925
contig_1843	0.0033	0.0107	0.64251208
contig_1851	0	0.0153	0.39525692
contig_1857	0	0.0203	0.330033
contig_1864	0.0083	0.0128	0.80263158
contig_1866	0.0114	0.0519	0.3457189
contig_187	0.0905	0.3207	0.30390082
contig_1876	0.0013	0.0073	0.65317919
contig_1879	0	0.0029	0.7751938
contig_188	0.0027	0.0441	0.23475046
contig_1880	0.0021	0.0053	0.79084967
contig_1882	0.0015	0.0114	0.53738318
contig_1883	0.002	0.0084	0.65217391
contig_1886	0	0.0099	0.50251256
contig_1887	0.0013	0.0097	0.57360406
contig_1888	0.0016	0.0153	0.45849802

contig_189	0.0046	0.0109	0.69856459
contig_1894	0.0018	0.0279	0.31134565
contig_1896	0.0004	0.0149	0.41767068
contig_19	0.0014	0.0146	0.46341463
contig_190	0.0008	0.0054	0.7012987
contig_1900	0.0051	0.0367	0.32334047
contig_1901	0.002	0.0426	0.22813688
contig_1903	0.0101	0.0357	0.43982495
contig_1905	0.0022	0.0264	0.33516484
contig_1906	0.0007	0.0186	0.37412587
contig_1908	0	0.017	0.37037037
contig_1913	0.0011	0.0178	0.39928058
contig_1918	0.0012	0.024	0.32941177
contig_1934	0.0026	0.0075	0.72
contig_1936	0.003	0.0035	0.96296296
contig_1938	0.0017	0.0228	0.35670732
contig_1940	0.002	0.002	1
contig_1941	0.0026	0.0089	0.66666667
contig_1943	0.0024	0.0188	0.43055556
contig_1948	0.0031	0.0107	0.63285024
contig_1950	0.0043	0.0242	0.41812866
contig_1952	0.0025	0.0261	0.34626039
contig_1953	0	0.0132	0.43103448
contig_1959	0.0018	0.0153	0.46640316
contig_1960	0.0008	0.0164	0.40909091
contig_1968	0.0096	0.0856	0.20502092
contig_1972	0.0009	0.0073	0.6300578
contig_1973	0.001	0.0203	0.3630363
contig_1977	0.012	0.0356	0.48245614
contig_1979	0.0019	0.0136	0.50423729
contig_1982	0.0075	0.0024	1.41129032
contig_1988	0	0.007	0.58823529
contig_1989	0.0051	0.021	0.48709677
contig_1992	0.0005	0.0179	0.37634409
contig_1993	0.0011	0.0059	0.69811321
contig_1994	0.0031	0.0154	0.51574803
contig_1996	0.0022	0.0177	0.44043321
contig_2005	0.0014	0.0061	0.70807453
contig_2007	0.0004	0.0109	0.49760766
contig_2008	0.0032	0.0184	0.46478873
contig_2009	0	0.0091	0.52356021
contig_201	0.0019	0.0019	1
contig_2011	0.0021	0.0078	0.67977528
contig_2014	0.0108	0.0585	0.30364964
contig_2016	0.006	0.0142	0.66115703
contig_2017	0.001	0.0107	0.53140097
contig_2018	0.0006	0.0088	0.56382979
contig_2019	0.0009	0.0117	0.50230415
contig_202	0.0012	0.0075	0.64

contig_2020	0.0009	0.0093	0.56476684
contig_2022	0.0125	0.0732	0.27043269
contig_2023	0.0116	0.07	0.27
contig_2028	0.0008	0.013	0.46956522
contig_203	0.0008	0.0149	0.43373494
contig_2032	0.0004	0.0036	0.76470588
contig_2034	0.002	0.0068	0.71428571
contig_2042	0.003	0.0203	0.4290429
contig_2045	0.0006	0.0116	0.49074074
contig_2048	0.0005	0.0181	0.37366548
contig_2051	0.0037	0.0128	0.60087719
contig_2052	0.0006	0.0086	0.56989247
contig_2055	0.0014	0.0138	0.4789916
contig_2060	0.0051	0.0419	0.29094412
contig_2062	0	0.0202	0.33112583
contig_2066	0	0.0041	0.70921986
contig_2068	0.0006	0.0205	0.34754098
contig_2070	0.0043	0.0179	0.5125448
contig_2071	0.0052	0.0353	0.33554084
contig_2074	0.0004	0.0404	0.20634921
contig_2075	0.0024	0.0253	0.35127479
contig_2076	0.0024	0.0113	0.58215962
contig_2078	0.0008	0.0104	0.52941177
contig_2080	0.0068	0.0662	0.22047244
contig_2081	0.0038	0.0115	0.64186047
contig_2083	0.0037	0.0107	0.66183575
contig_2087	0.0022	0.0218	0.3836478
contig_2088	0.0006	0.0075	0.60571429
contig_2089	0.0003	0.0158	0.39922481
contig_209	0.0037	0.0137	0.57805907
contig_2092	0.0006	0.0218	0.33333333
contig_2097	0.0016	0.0171	0.42804428
contig_2099	0.0031	0.0376	0.27521008
contig_21	0.0068	0.018	0.6
contig_2101	0.0013	0.0135	0.48085106
contig_2102	0.0006	0.009	0.55789474
contig_2106	0.0022	0.0128	0.53508772
contig_2107	0.0007	0.0398	0.21485944
contig_2108	0.0087	0.0513	0.3050571
contig_2109	0.002	0.0175	0.43636364
contig_2115	0.0017	0.0059	0.73584906
contig_2116	0.0105	0.0624	0.28314917
contig_2117	0	0.0072	0.58139535
contig_2118	0.0038	0.0121	0.62443439
contig_2119	0.0039	0.0126	0.61504425
contig_2120	0.0029	0.0126	0.57079646
contig_2121	0.0039	0.0167	0.52059925
contig_2124	0	0.0136	0.42372881
contig_2129	0.0036	0.0247	0.39193084

contig_213	0.0022	0.0098	0.61616162
contig_2131	0.002	0.0218	0.37735849
contig_2137	0.0009	0.0158	0.42248062
contig_2138	0.003	0.022	0.40625
contig_214	0.005	0.0299	0.37593985
contig_2145	0.003	0.0192	0.44520548
contig_215	0.0085	0.0046	1.26712329
contig_2150	0.0027	0.0131	0.54978355
contig_2151	0.0004	0.0638	0.14092141
contig_2153	0.0031	0.009	0.68947368
contig_2156	0.0012	0.011	0.53333333
contig_2157	0.0068	0.0262	0.4640884
contig_216	0.0037	0.0235	0.40895522
contig_2161	0.0008	0.0117	0.49769585
contig_2163	0.0021	0.0044	0.84027778
contig_2164	0.0023	0.0174	0.44890511
contig_2169	0.0004	0.0142	0.42975207
contig_217	0.0013	0.0043	0.79020979
contig_2170	0.002	0.0077	0.6779661
contig_2172	0.0059	0.0139	0.66527197
contig_2178	0.0006	0.0149	0.42570281
contig_2179	0.0039	0.0347	0.31096197
contig_218	0	0.014	0.41666667
contig_2182	0.0009	0.0124	0.48660714
contig_2187	0.0246	0.0535	0.54488189
contig_2189	0.0031	0.0238	0.38757396
contig_2196	0.0007	0.011	0.50952381
contig_2197	0.0077	0.0332	0.40972222
contig_2198	0.0042	0.0123	0.6367713
contig_2199	0.0166	0.0172	0.97794118
contig_220	0.0019	0.0269	0.32249323
contig_2201	0.0028	0.0146	0.5203252
contig_2202	0.0041	0.021	0.45483871
contig_2203	0.0007	0.0143	0.44032922
contig_2208	0.0045	0.0256	0.40730337
contig_2209	0.0007	0.0476	0.18576389
contig_221	0.0034	0.0401	0.26746507
contig_2214	0.0081	0.0231	0.5468278
contig_2216	0.0017	0.0211	0.37620579
contig_2217	0.0006	0.0145	0.43265306
contig_222	0.0008	0.0258	0.30167598
contig_2220	0.0039	0.0059	0.87421384
contig_2221	0.0004	0.0139	0.43514644
contig_2223	0	0.01	0.5
contig_2224	0.003	0.0075	0.74285714
contig_2225	0.0119	0.0646	0.29356568
contig_2226	0.0045	0.0116	0.6712963
contig_2228	0.0029	0.011	0.61428571
contig_2229	0.0061	0.0516	0.26136364

contig_2234	0.0013	0.0151	0.4501992
contig_2237	0.0047	0.0124	0.65625
contig_2239	0.0018	0.0321	0.28028504
contig_2240	0.0038	0.0034	1.02985075
contig_2242	0.0046	0.024	0.42941177
contig_2246	0.0013	0.0094	0.58247423
contig_2248	0.0009	0.0106	0.52912621
contig_2251	0.0009	0.0149	0.437751
contig_2252	0.001	0.0347	0.24608501
contig_2253	0	0.0116	0.46296296
contig_2257	0.0028	0.0153	0.50592885
contig_226	0.0058	0.0241	0.46334311
contig_2261	0.0006	0.0333	0.2448037
contig_2263	0.0023	0.0037	0.89781022
contig_2265	0.0053	0	1.53
contig_2268	0.0028	0.0141	0.53112033
contig_2270	0.0033	0.0077	0.75141243
contig_2272	0.001	0.0162	0.41984733
contig_2275	0.0012	0.0166	0.42105263
contig_2279	0.0031	0.027	0.35405405
contig_2280	0.0004	0.0065	0.63030303
contig_2285	0.0009	0.0092	0.56770833
contig_2286	0.0009	0.0123	0.48878924
contig_2287	0	0.0043	0.6993007
contig_2289	0.003	0.0317	0.3117506
contig_2290	0.0008	0.0116	0.5
contig_2293	0.0023	0.0071	0.71929825
contig_2296	0.0014	0.0257	0.31932773
contig_2298	0.0009	0.0201	0.36212625
contig_2299	0.0031	0.0113	0.61502347
contig_2302	0.0027	0.0694	0.15994962
contig_2303	0.001	0.0135	0.46808511
contig_2306	0.0061	0.0103	0.79310345
contig_2307	0.0012	0.0129	0.48908297
contig_2308	0.0012	0.0096	0.57142857
contig_2309	0.0013	0.0158	0.4379845
contig_2310	0.003	0.0128	0.57017544
contig_2318	0.0032	0.0131	0.57142857
contig_2323	0.0016	0.0078	0.65168539
contig_2325	0.003	0.0291	0.33248082
contig_2328	0.0024	0.0115	0.57674419
contig_233	0	0.004	0.71428571
contig_2330	0.0014	0.0113	0.53521127
contig_2331	0.0002	0.0092	0.53125
contig_2332	0.0003	0.0082	0.56593407
contig_2337	0.0019	0.0023	0.96747968
contig_2340	0.0013	0.0143	0.46502058
contig_2342	0.0038	0.0328	0.32242991
contig_2345	0.0021	0.0165	0.45660377

contig_2347	0.0006	0.0033	0.79699248
contig_2348	0.0024	0.0053	0.81045752
contig_2349	0	0.0122	0.45045045
contig_235	0.0074	0.0143	0.71604938
contig_236	0.0021	0.0135	0.51489362
contig_2362	0.0003	0.0075	0.58857143
contig_2363	0.0022	0.009	0.64210526
contig_2372	0.0008	0.0192	0.36986301
contig_2373	0.021	0.185	0.15897436
contig_2374	0.0026	0.0135	0.53617021
contig_2378	0.0002	0.0191	0.35051546
contig_2379	0.0021	0.0074	0.6954023
contig_2380	0	0.0133	0.42918455
contig_2381	0	0.0087	0.53475936
contig_2382	0.0004	0.0109	0.49760766
contig_2383	0.0095	0.0165	0.73584906
contig_2386	0.0004	0.0452	0.1884058
contig_2387	0.0024	0.0166	0.46616541
contig_2389	0.0015	0.0074	0.66091954
contig_2392	0.0026	0.0276	0.33510638
contig_2393	0.0076	0	1.76
contig_2396	0.0023	0.0212	0.39423077
contig_2397	0.0044	0.0353	0.3178808
contig_240	0.0029	0.0276	0.34308511
contig_2401	0.0012	0.0146	0.45528455
contig_2406	0.0024	0.0088	0.65957447
contig_2407	0.0016	0.0112	0.54716981
contig_2408	0.003	0.0077	0.73446328
contig_2410	0.0006	0.0055	0.68387097
contig_2411	0.0003	0.024	0.30294118
contig_2412	0.0036	0.0071	0.79532164
contig_2413	0.0088	0.0224	0.58024691
contig_2414	0.0026	0.0189	0.43598616
contig_2415	0.0003	0.011	0.49047619
contig_2417	0.0019	0.0163	0.45247148
contig_2418	0.0011	0.0119	0.50684932
contig_2420	0.0014	0.0224	0.35185185
contig_2422	0	0.0128	0.43859649
contig_2423	0.0028	0.0076	0.72727273
contig_2425	0.0031	0.0469	0.23022847
contig_2426	0	0.0139	0.41841004
contig_2427	0.0036	0.0344	0.30630631
contig_2429	0.0009	0.0116	0.50462963
contig_243	0.0047	0.0162	0.5610687
contig_2435	0.0023	0.0175	0.44727273
contig_2439	0.0013	0.008	0.62777778
contig_244	0	0.0045	0.68965517
contig_2443	0.0042	0.0322	0.33649289
contig_245	0.0019	0.0112	0.56132076

contig_2451	0.0038	0.0274	0.36898396
contig_2452	0.0026	0.0146	0.51219512
contig_2458	0.0034	0.0175	0.48727273
contig_2459	0.0018	0.0093	0.61139896
contig_2463	0.0018	0.0152	0.46825397
contig_2465	0.0006	0.0058	0.67088608
contig_247	0.0048	0.0112	0.69811321
contig_2470	0.0012	0.0066	0.6746988
contig_2476	0.0026	0.008	0.7
contig_2488	0.0014	0.0095	0.58461539
contig_2489	0.0011	0.0402	0.22111554
contig_249	0	0.009	0.52631579
contig_2492	0.0011	0.0322	0.26303318
contig_2493	0.003	0.0096	0.66326531
contig_2497	0.006	0.0496	0.26845638
contig_2498	0.0051	0.0313	0.36561743
contig_2499	0.0016	0.026	0.32222222
contig_250	0.0119	0.0227	0.66972477
contig_2501	0.0024	0.0139	0.51882845
contig_2502	0.0011	0.0244	0.32267442
contig_2503	0.0002	0.0055	0.65806452
contig_2504	0.0006	0.0043	0.74125874
contig_2505	0.0008	0.0094	0.55670103
contig_2506	0.0057	0.022	0.490625
contig_2508	0.0027	0.0138	0.53361345
contig_2510	0.0064	0.0104	0.80392157
contig_2512	0.0008	0.016	0.41538462
contig_2516	0.0054	0.005	1.02666667
contig_2517	0.0013	0.0168	0.42164179
contig_2518	0.0002	0.0035	0.75555556
contig_2519	0	0.0061	0.62111801
contig_2524	0	0.0043	0.6993007
contig_2533	0.0012	0.0265	0.30684932
contig_2540	0.0058	0.0135	0.67234043
contig_2541	0.0061	0.0096	0.82142857
contig_2542	0	0.0096	0.51020408
contig_2545	0.0032	0.0061	0.81987578
contig_2547	0.0026	0.0377	0.26415094
contig_2551	0.0002	0.0179	0.3655914
contig_2555	0.0094	0.2023	0.09138012
contig_2558	0.0042	0.0102	0.7029703
contig_2559	0.0014	0.0098	0.57575758
contig_256	0.0057	0.0226	0.48159509
contig_2567	0.0006	0.0177	0.38267148
contig_2568	0.001	0.0506	0.18151815
contig_2573	0.0028	0.0451	0.2323049
contig_2575	0	0.0108	0.48076923
contig_2576	0.0011	0.0709	0.13720643
contig_2577	0.0155	0.0313	0.61743341

contig_2578	0.0083	0.068	0.23461539
contig_258	0.0018	0.0195	0.4
contig_2581	0.0019	0.0074	0.68390805
contig_2584	0.001	0.0254	0.31073446
contig_2589	0.0021	0.0211	0.38906752
contig_259	0.0047	0.0041	1.04255319
contig_2591	0.0072	0.0335	0.3954023
contig_2594	0	0.0146	0.40650407
contig_26	0.001	0.0133	0.472103
contig_2600	0.0005	0.0084	0.57065217
contig_2602	0.0015	0.0083	0.6284153
contig_2608	0.0027	0.0243	0.37026239
contig_2611	0.0018	0.0044	0.81944444
contig_2612	0.0027	0.0029	0.98449612
contig_2614	0.006	0.0319	0.38186158
contig_2620	0.0063	0.0138	0.68487395
contig_2623	0.0062	0.0378	0.33891213
contig_263	0.0008	0.0286	0.27979275
contig_2632	0.0008	0.0282	0.28272251
contig_2636	0.0093	0.0267	0.52588556
contig_2639	0.0013	0.0123	0.50672646
contig_264	0	0.0072	0.58139535
contig_2640	0.0023	0.0084	0.66847826
contig_2642	0.0085	0.0569	0.27653214
contig_2643	0.0005	0.009	0.55263158
contig_2645	0.0006	0.0207	0.34527687
contig_2646	0.0083	0.0155	0.71764706
contig_2647	0.0034	0.0364	0.2887931
contig_2649	0.0039	0.011	0.66190476
contig_265	0.0016	0.0147	0.46963563
contig_2650	0.0009	0.011	0.51904762
contig_2655	0.0027	0.0363	0.27429806
contig_2656	0.0006	0.0138	0.44537815
contig_2661	0.0012	0.013	0.48695652
contig_2666	0	0.0211	0.32154341
contig_2668	0.0003	0.013	0.44782609
contig_2669	0.0024	0.0178	0.44604317
contig_2673	0.0143	0.0811	0.26673985
contig_2676	0.0019	0.0067	0.71257485
contig_268	0.0028	0.0257	0.35854342
contig_2680	0.0024	0.0235	0.37014925
contig_2683	0.0044	0.0202	0.47682119
contig_2688	0	0.0119	0.45662101
contig_269	0.002	0.0292	0.30612245
contig_2691	0.0072	0.0103	0.84729064
contig_2696	0.002	0.0199	0.40133779
contig_2699	0.001	0.007	0.64705882
contig_270	0.0011	0.0174	0.40510949
contig_2702	0.0024	0.015	0.496

contig_2704	0	0.0425	0.19047619
contig_2705	0.0047	0.0167	0.5505618
contig_2708	0	0.0287	0.25839793
contig_271	0	0.0334	0.23041475
contig_2713	0.0042	0.0146	0.57723577
contig_272	0.0012	0.0181	0.39857651
contig_2720	0.0041	0.0212	0.45192308
contig_2722	0.0308	0.0659	0.53754941
contig_2723	0.0003	0.0091	0.53926702
contig_2725	0	0.0135	0.42553192
contig_2728	0.0076	0.017	0.65185185
contig_2729	0.0006	0.0077	0.59887006
contig_273	0.0036	0.0198	0.45637584
contig_2736	0	0.0396	0.2016129
contig_2737	0.0013	0.0118	0.51834862
contig_2740	0.0009	0.0132	0.46982759
contig_2742	0.0018	0.0158	0.45736434
contig_2748	0.003	0.0088	0.69148936
contig_2749	0.0028	0.0191	0.43986254
contig_275	0	0.0074	0.57471264
contig_2751	0	0.0054	0.64935065
contig_2753	0.002	0.0215	0.38095238
contig_2754	0.0004	0.0083	0.56830601
contig_2757	0.0007	0.0076	0.60795455
contig_2761	0.0033	0.013	0.57826087
contig_2767	0.0089	0.0322	0.4478673
contig_2768	0.0054	0.0158	0.59689923
contig_277	0.0013	0.0041	0.80141844
contig_2771	0.0077	0.0023	1.43902439
contig_2773	0.0009	0.0265	0.29863014
contig_2776	0.0011	0.0038	0.80434783
contig_2778	0.0008	0.0212	0.34615385
contig_2779	0.0027	0.0214	0.4044586
contig_2780	0.0009	0.0057	0.69426752
contig_2781	0.0076	0.0804	0.19469027
contig_2783	0.0022	0.012	0.55454546
contig_2784	0.0006	0.0204	0.34868421
contig_2785	0.0026	0.0477	0.21837088
contig_2791	0.0051	0.0048	1.02027027
contig_2795	0.0013	0.0155	0.44313726
contig_2796	0.0022	0.0154	0.48031496
contig_2797	0.0095	0.0318	0.46650718
contig_2800	0.0015	0.0324	0.27122642
contig_2801	0.0031	0.0375	0.27578947
contig_2805	0.0014	0.0325	0.26823529
contig_2806	0.0015	0.0149	0.46184739
contig_2808	0.0018	0.0102	0.58415842
contig_281	0.0029	0.0424	0.24618321
contig_2810	0.0021	0.0155	0.4745098

contig_2811	0.0013	0.0344	0.25450451
contig_2812	0.0019	0.0233	0.35735736
contig_2813	0.0011	0.0197	0.37373737
contig_2816	0.0054	0.0146	0.62601626
contig_2818	0.0005	0.0161	0.40229885
contig_282	0.0023	0.0044	0.85416667
contig_2820	0.0026	0.0092	0.65625
contig_2823	0.0007	0.0163	0.40684411
contig_2831	0.0039	0.0133	0.59656652
contig_2834	0.0033	0.0202	0.44039735
contig_2835	0.0024	0.0111	0.58767773
contig_2837	0.0012	0.0128	0.49122807
contig_2839	0.0043	0.0238	0.42307692
contig_2842	0	0.0129	0.43668122
contig_2844	0	0.0238	0.29585799
contig_2845	0.0004	0.0181	0.37010676
contig_2850	0.005	0.015	0.6
contig_2853	0.0023	0.0148	0.49596774
contig_2858	0.002	0.004	0.85714286
contig_2860	0.0004	0.0223	0.32198142
contig_2865	0.0036	0.0117	0.62672811
contig_2870	0.003	0.0594	0.18731989
contig_2874	0.0083	0.0738	0.21837709
contig_2877	0.0031	0.0169	0.48698885
contig_2879	0.0011	0.0179	0.39784946
contig_2883	0.0014	0.007	0.67058824
contig_2887	0.001	0.035	0.24444444
contig_2888	0.0018	0.0339	0.26879271
contig_2892	0.0005	0.0108	0.50480769
contig_2894	0.0035	0.0179	0.48387097
contig_2895	0.0019	0.0078	0.66853933
contig_2896	0.0017	0.0068	0.69642857
contig_2898	0.0004	0.0105	0.50731707
contig_2900	0.0027	0.0358	0.27729258
contig_2901	0	0.0284	0.26041667
contig_2907	0.0016	0.0136	0.49152542
contig_2908	0.003	0.0123	0.58295964
contig_291	0.0069	0.0785	0.19096045
contig_2913	0.0014	0.0517	0.18476499
contig_2915	0.0012	0.0025	0.896
contig_2916	0.0134	0.0344	0.52702703
contig_2919	0.0032	0.012	0.6
contig_2920	0.001	0.008	0.61111111
contig_2921	0.004	0.0149	0.562249
contig_2926	0.0016	0.0092	0.60416667
contig_2927	0.0013	0.04	0.226
contig_2928	0.0026	0.0132	0.54310345
contig_2930	0.0007	0.0064	0.65243902
contig_2933	0.0056	0.0069	0.92307692

contig_2938	0.0012	0.0186	0.39160839
contig_294	0.0018	0.0156	0.4609375
contig_2940	0.0036	0.009	0.71578947
contig_2943	0.0112	0.0522	0.34083601
contig_2945	0.0029	0.0077	0.72881356
contig_2947	0.0018	0.0384	0.24380165
contig_2952	0.0024	0.0403	0.24652088
contig_2956	0	0.0219	0.31347962
contig_2959	0.0009	0.0125	0.48444444
contig_2963	0.0013	0.0276	0.30053192
contig_2964	0.0026	0.0215	0.4
contig_2968	0.0058	0.0532	0.25
contig_2973	0.0009	0.015	0.436
contig_2974	0	0.0047	0.68027211
contig_2975	0.0015	0.0236	0.34226191
contig_2977	0.0024	0.0579	0.1826215
contig_2980	0.0011	0.0067	0.66467066
contig_2983	0.0007	0.0035	0.79259259
contig_2985	0.0012	0.0263	0.30853995
contig_2989	0.0055	0.0123	0.69506727
contig_2992	0.0015	0.0175	0.41818182
contig_2995	0.0021	0.012	0.55
contig_2997	0.0168	0.0328	0.62616822
contig_3000	0.0005	0.0072	0.61046512
contig_3003	0.003	0.0498	0.2173913
contig_3005	0.0015	0.007	0.67647059
contig_3006	0.0038	0.022	0.43125
contig_3007	0.0006	0.0205	0.34754098
contig_3008	0.0019	0.0061	0.73913044
contig_3014	0.001	0.0173	0.4029304
contig_3017	0	0.0686	0.12722646
contig_3018	0.0036	0.0213	0.43450479
contig_3020	0	0.0172	0.36764706
contig_3024	0.0027	0.039	0.25918367
contig_3027	0.0012	0.0107	0.5410628
contig_3031	0.0022	0.0103	0.60098522
contig_3039	0.0031	0.0187	0.45644599
contig_3041	0.003	0.0386	0.26748971
contig_3042	0.0012	0.0121	0.50678733
contig_3043	0.0041	0.0266	0.3852459
contig_3047	0.0057	0.0549	0.24191063
contig_3049	0.0012	0.0425	0.21333333
contig_305	0.0013	0.0262	0.3121547
contig_3053	0.001	0.0105	0.53658537
contig_3054	0.0109	0.0168	0.77985075
contig_3057	0.0009	0.0256	0.30617978
contig_3062	0.0043	0.0358	0.31222707
contig_3064	0.0009	0.0126	0.48230089
contig_3065	0.0006	0.0064	0.64634146

contig_3067	0.0007	0.0086	0.57526882
contig_3070	0	0.0179	0.35842294
contig_3074	0.0039	0.0141	0.57676349
contig_3078	0.0024	0.012	0.56363636
contig_3080	0.0053	0.0225	0.47076923
contig_3081	0.0013	0.0122	0.50900901
contig_3085	0.0045	0.0088	0.7712766
contig_3086	0.0113	0.0266	0.58196721
contig_3091	0	0.0087	0.53475936
contig_3092	0.0073	0.0221	0.53894081
contig_3094	0.0067	0.0332	0.38657407
contig_3095	0.0034	0.0219	0.4200627
contig_3100	0.003	0.0061	0.80745342
contig_3102	0.0005	0.0193	0.35836178
contig_3111	0.0021	0.0251	0.34472935
contig_3117	0.0054	0.0238	0.4556213
contig_312	0	0.007	0.58823529
contig_3124	0.0029	0.0096	0.65816327
contig_3127	0.0045	0.0205	0.47540984
contig_3130	0.0018	0.0063	0.72392638
contig_3142	0.0029	0.0094	0.66494845
contig_3144	0.0045	0.0151	0.57768924
contig_3145	0	0.0055	0.64516129
contig_3148	0.0014	0.0115	0.53023256
contig_3154	0.0007	0.0114	0.5
contig_3156	0.007	0.016	0.65384615
contig_3157	0.0018	0.0128	0.51754386
contig_316	0.0012	0.0148	0.4516129
contig_3163	0.0066	0.01	0.83
contig_3165	0.0019	0.0242	0.34795322
contig_3167	0.0019	0.0104	0.58333333
contig_3169	0.0102	0.0233	0.60660661
contig_3171	0.0062	0.0465	0.28672566
contig_3176	0	0.0261	0.27700831
contig_3180	0.0004	0.0086	0.55913979
contig_3190	0.0008	0.0102	0.53465347
contig_3193	0.0028	0.0066	0.77108434
contig_3199	0	0.0194	0.34013605
contig_3201	0.0003	0.0123	0.46188341
contig_3205	0.0012	0.0133	0.4806867
contig_3208	0.0003	0.0109	0.49282297
contig_321	0	0.0226	0.30674847
contig_3210	0.0077	0.028	0.46578947
contig_3211	0.0018	0.0111	0.55924171
contig_3212	0.0012	0.0111	0.53080569
contig_3214	0.0014	0.0151	0.45418327
contig_3217	0.004	0.0042	0.98591549
contig_3218	0.0036	0.0219	0.42633229
contig_3219	0.0019	0.0144	0.48770492

contig_3224	0.002	0.0228	0.36585366
contig_3225	0.0012	0.0243	0.32653061
contig_3227	0	0.0315	0.24096386
contig_3228	0.0014	0.0026	0.90476191
contig_3229	0.0041	0.0075	0.80571429
contig_3231	0.0021	0.0199	0.40468227
contig_3232	0.0015	0.0097	0.58375635
contig_3233	0.015	0.0324	0.58962264
contig_324	0.0022	0.0046	0.83561644
contig_3244	0.0014	0.0203	0.37623762
contig_3245	0.0022	0.0125	0.54222222
contig_3246	0.0039	0.0133	0.59656652
contig_3248	0.0048	0.0175	0.53818182
contig_3249	0.0016	0.023	0.35151515
contig_325	0.0006	0.0083	0.57923497
contig_3250	0.0044	0.0195	0.48813559
contig_3251	0.005	0.0312	0.36407767
contig_3252	0.0009	0.0074	0.62643678
contig_3253	0.0027	0.0167	0.47565543
contig_3257	0.001	0.0067	0.65868264
contig_3260	0.001	0.0068	0.65476191
contig_3265	0.0006	0.0062	0.65432099
contig_3267	0.0021	0.0336	0.27752294
contig_3270	0.0109	0.0425	0.39809524
contig_3271	0.0024	0.0203	0.40924092
contig_3273	0.0045	0.0251	0.41310541
contig_3274	0.003	0.0088	0.69148936
contig_3275	0.0014	0.022	0.35625
contig_3277	0.0039	0.0097	0.70558376
contig_3279	0.0043	0.0159	0.55212355
contig_3282	0.0076	0.0263	0.48484849
contig_3283	0.0015	0.0086	0.61827957
contig_3292	0.0012	0.0252	0.31818182
contig_3294	0.0031	0.0135	0.55744681
contig_3297	0.0224	0.0942	0.3109405
contig_3300	0.01	0.012	0.90909091
contig_3302	0.0019	0.0096	0.60714286
contig_3303	0.0005	0.0664	0.13743456
contig_3306	0.0102	0.0629	0.27709191
contig_3309	0	0.0552	0.15337423
contig_331	0	0.0156	0.390625
contig_3311	0.001	0.0094	0.56701031
contig_3323	0	0.0338	0.2283105
contig_3327	0.0014	0.0064	0.69512195
contig_3333	0.0001	0.0202	0.33443709
contig_3337	0.0028	0.0176	0.46376812
contig_3348	0.0047	0.0495	0.24705882
contig_3349	0.0003	0.0204	0.33881579
contig_335	0.0135	0.0454	0.42418773

contig_3350	0.0163	0.0444	0.48345588
contig_3366	0.003	0.0155	0.50980392
contig_3372	0.0018	0.0157	0.45914397
contig_3375	0.0013	0.0136	0.47881356
contig_3377	0	0.0432	0.18796993
contig_3379	0.0029	0.013	0.56086957
contig_3380	0.0003	0.0187	0.35888502
contig_3384	0.0014	0.0193	0.3890785
contig_3387	0.0041	0.0353	0.31125828
contig_339	0.0024	0.0073	0.71676301
contig_3390	0	0.0229	0.30395137
contig_3394	0.0023	0.0127	0.54185022
contig_3397	0.0066	0.0212	0.53205128
contig_3400	0.0008	0.0185	0.37894737
contig_3401	0.0001	0.0102	0.5
contig_3407	0	0.0131	0.43290043
contig_3408	0.0003	0.0074	0.59195402
contig_341	0	0.008	0.55555556
contig_3418	0.002	0.0069	0.71005917
contig_3419	0.0012	0.0217	0.3533123
contig_3421	0.0014	0.0137	0.48101266
contig_343	0.0025	0.0135	0.53191489
contig_3430	0.0004	0.0062	0.64197531
contig_3431	0.0032	0.016	0.50769231
contig_3433	0.0024	0.0051	0.82119205
contig_3443	0.0008	0.01	0.54
contig_3444	0.0034	0.021	0.43225807
contig_3445	0	0.018	0.35714286
contig_3446	0.0021	0.0227	0.37003058
contig_3448	0.0063	0.0259	0.454039
contig_3452	0.0027	0.0109	0.6076555
contig_3453	0.0038	0.0057	0.87898089
contig_346	0.0142	0.0447	0.44241316
contig_3460	0.0059	0.0402	0.31673307
contig_3462	0.0019	0.0248	0.34195402
contig_3463	0	0.01	0.5
contig_3467	0.0011	0.0065	0.67272727
contig_347	0.0036	0.0087	0.72727273
contig_3471	0.0034	0.015	0.536
contig_3475	0	0.0241	0.29325513
contig_3479	0.0156	0.0502	0.42524917
contig_3483	0.0007	0.0349	0.23830735
contig_3488	0.0042	0.0337	0.32494279
contig_349	0.0015	0.0068	0.68452381
contig_3496	0.0023	0.0381	0.25571726
contig_3498	0.0025	0.0076	0.71022727
contig_3501	0.0025	0.0345	0.28089888
contig_3505	0.0019	0.0114	0.55607477
contig_3510	0	0.0064	0.6097561

contig_3512	0.0013	0.0256	0.31741573
contig_3514	0.0078	0.028	0.46842105
contig_3515	0.0088	0.0342	0.42533937
contig_3517	0.0008	0.0115	0.50232558
contig_352	0.0026	0.0034	0.94029851
contig_3522	0	0.0111	0.47393365
contig_3528	0.0065	0.0151	0.65737052
contig_3530	0.0045	0.0109	0.6937799
contig_3531	0.0033	0.0507	0.21911038
contig_3532	0	0.0259	0.27855153
contig_3534	0.0007	0.0023	0.8699187
contig_3538	0.0009	0.0162	0.41603053
contig_354	0.0045	0.0158	0.5620155
contig_3543	0.0013	0.0043	0.79020979
contig_3544	0.0019	0.0095	0.61025641
contig_3546	0.0025	0.0072	0.72674419
contig_3547	0.0377	0.2068	0.22001845
contig_355	0	0.01	0.5
contig_3553	0.0025	0.0126	0.55309735
contig_3556	0.0039	0.0246	0.4017341
contig_3558	0.0026	0.1056	0.10899654
contig_3560	0.0096	0.0082	1.07692308
contig_3562	0.0012	0.0079	0.62569832
contig_3564	0.0032	0.0154	0.51968504
contig_3567	0.0041	0.0202	0.46688742
contig_3570	0.001	0.0122	0.4954955
contig_3571	0.0015	0.0086	0.61827957
contig_3574	0.0033	0.0529	0.21144674
contig_3576	0.0013	0.0027	0.88976378
contig_3578	0.0594	0.1371	0.4717879
contig_358	0.002	0.0078	0.6741573
contig_3580	0.0045	0.0143	0.59670782
contig_3584	0.0043	0.0335	0.32873563
contig_3587	0.0018	0.0048	0.7972973
contig_3590	0.0023	0.0117	0.56682028
contig_3591	0.0011	0.0227	0.33944954
contig_3597	0	0.0222	0.31055901
contig_3601	0.013	0.0366	0.49356223
contig_3602	0.0063	0.0037	1.18978102
contig_3605	0.0048	0.0276	0.39361702
contig_3607	0.0015	0.0048	0.77702703
contig_3608	0.002	0.0099	0.60301508
contig_3609	0.0011	0.0064	0.67682927
contig_361	0.0039	0.0215	0.44126984
contig_3610	0.0042	0.0045	0.97931035
contig_3611	0.001	0.0174	0.40145985
contig_3612	0.0013	0.0047	0.76870748
contig_3613	0.0026	0.0373	0.26638478
contig_3616	0	0.0038	0.72463768

contig_3617	0.0029	0.0131	0.55844156
contig_3619	0.0058	0.0149	0.63453815
contig_3623	0.0004	0.0084	0.56521739
contig_3628	0.0019	0.0054	0.77272727
contig_3634	0.0057	0.0066	0.94578313
contig_3635	0.0068	0.0296	0.42424242
contig_3636	0.01	0.0357	0.43763676
contig_364	0.0042	0.0485	0.24273504
contig_3642	0.0034	0.0086	0.72043011
contig_3643	0	0.0207	0.3257329
contig_3648	0.0056	0.0292	0.39795918
contig_3658	0.0075	0.0168	0.65298508
contig_3659	0	0.0321	0.23752969
contig_3660	0.0011	0	1.11
contig_3661	0.0402	0.0391	1.02240326
contig_3664	0.0118	0.0154	0.85826772
contig_3671	0.0028	0.0196	0.43243243
contig_3673	0.0065	0.0448	0.30109489
contig_3677	0.0135	0.0962	0.2212806
contig_3678	0.0306	0.0731	0.48856799
contig_3680	0.0082	0.0381	0.37837838
contig_3685	0.0044	0.0043	1.00699301
contig_3686	0.0044	0.0159	0.55598456
contig_3689	0.0047	0.0543	0.22861586
contig_3691	0.0028	0.016	0.49230769
contig_3695	0.0017	0.0015	1.0173913
contig_3696	0.0013	0.0309	0.27628362
contig_3699	0.0029	0.0211	0.414791
contig_370	0	0.0602	0.14245014
contig_3701	0	0.0115	0.46511628
contig_3706	0.0052	0.0146	0.61788618
contig_371	0.0125	0.0193	0.76791809
contig_3710	0.0069	0.0249	0.48424069
contig_3713	0	0.009	0.52631579
contig_3716	0.0188	0.0569	0.43049327
contig_3717	0.0033	0.0087	0.71122995
contig_3724	0	0.0196	0.33783784
contig_3725	0.0029	0.0236	0.38392857
contig_3727	0.0032	0.0092	0.6875
contig_3728	0.0019	0.0029	0.92248062
contig_3729	0.0008	0.0028	0.84375
contig_373	0.0011	0.0083	0.60655738
contig_3730	0.0076	0.2115	0.07945824
contig_3731	0.0077	0.0574	0.26261128
contig_3734	0.0014	0.0013	1.00884956
contig_3735	0.013	0.0124	1.02678571
contig_3738	0	0.0093	0.51813472
contig_3739	0.0027	0.0145	0.51836735
contig_374	0.0052	0.0439	0.28200371

contig_3741	0.0072	0.0207	0.56026059
contig_3742	0.0045	0.0777	0.16533637
contig_3743	0.0023	0.0151	0.49003984
contig_3744	0.0105	0.0217	0.6466877
contig_3746	0.0071	0.0301	0.42643392
contig_3748	0	0.0152	0.3968254
contig_375	0	0.0226	0.30674847
contig_3753	0.0017	0.0337	0.26773455
contig_3756	0.0027	0.01	0.635
contig_3759	0.0019	0.0193	0.40614335
contig_3760	0.0062	0.0255	0.45633803
contig_3761	0	0.0146	0.40650407
contig_3762	0.0035	0.0268	0.36684783
contig_3767	0.0009	0.0548	0.16820988
contig_3768	0	0.0186	0.34965035
contig_3769	0.0009	0.0053	0.7124183
contig_3774	0	0.0264	0.27472528
contig_3779	0.0073	0.0263	0.47658402
contig_3782	0.0012	0.0243	0.32653061
contig_3783	0.0045	0.0236	0.43154762
contig_3786	0.0036	0.0184	0.47887324
contig_3787	0.0073	0.0456	0.31115108
contig_3789	0.003	0.0055	0.83870968
contig_3793	0.0019	0.0338	0.2716895
contig_3795	0.0014	0.0473	0.19895288
contig_3796	0.0035	0.0227	0.41284404
contig_380	0.0018	0.0099	0.59296482
contig_3802	0.0006	0.0114	0.4953271
contig_3807	0.0138	0.0603	0.33854908
contig_3809	0.0009	0.0117	0.50230415
contig_3812	0.0143	0.004	1.73571429
contig_3814	0.0049	0.0458	0.26702509
contig_3815	0.0073	0.0033	1.30075188
contig_3816	0.0025	0.0104	0.6127451
contig_3818	0.0012	0.0149	0.4497992
contig_3825	0.0274	0.0803	0.41417497
contig_3826	0.0024	0.004	0.88571429
contig_3827	0.0261	0.4181	0.08432609
contig_3828	0.0036	0.0196	0.45945946
contig_3831	0.0104	0.0249	0.58452722
contig_3834	0	0.0042	0.70422535
contig_3845	0.0012	0.0146	0.45528455
contig_3846	0.0031	0.011	0.62380952
contig_3848	0.0047	0.0563	0.22171946
contig_3849	0.0012	0.0377	0.23480084
contig_385	0.0051	0.0174	0.55109489
contig_3850	0.0003	0.0023	0.83739837
contig_3852	0.0008	0.0329	0.25174825
contig_3853	0	0.0676	0.12886598

contig_3855	0.0212	0.0696	0.3919598
contig_3857	0.0008	0.0098	0.54545455
contig_3860	0.0062	0.0257	0.45378151
contig_3861	0.0023	0.0078	0.69101124
contig_3866	0.0019	0.0115	0.55348837
contig_3867	0.0024	0.022	0.3875
contig_3868	0	0.0161	0.38314176
contig_3869	0.0103	0.0287	0.5245478
contig_387	0.0036	0.0067	0.81437126
contig_3870	0.0011	0.0078	0.62359551
contig_3872	0.0009	0.0528	0.17356688
contig_3874	0.0006	0.0573	0.15750372
contig_3877	0	0.0119	0.45662101
contig_3880	0.0012	0.0111	0.53080569
contig_3881	0.027	0.0176	1.34057971
contig_3882	0.0168	0.0419	0.51637765
contig_3884	0.0004	0.0102	0.51485149
contig_3886	0.005	0.0409	0.29469548
contig_3887	0	0.0282	0.26178011
contig_3891	0.0018	0.028	0.31052632
contig_3892	0.0106	0.019	0.71034483
contig_3899	0.0022	0.021	0.39354839
contig_3901	0.0059	0.0213	0.50798722
contig_3902	0.0006	0.0269	0.28726287
contig_3905	0.0068	0.0462	0.29893238
contig_3908	0.0004	0.0077	0.58757062
contig_391	0.0032	0.0109	0.63157895
contig_3910	0.0017	0.0096	0.59693878
contig_3911	0.0003	0.0085	0.55675676
contig_3913	0.0017	0.0274	0.31283423
contig_3914	0	0.0296	0.25252525
contig_3916	0.0014	0.0091	0.59685864
contig_3922	0.0008	0.0073	0.62427746
contig_3923	0.002	0.0103	0.59113301
contig_3924	0.0014	0.0371	0.24203822
contig_3925	0.0068	0.0202	0.55629139
contig_3926	0.0037	0.0165	0.51698113
contig_3927	0.0019	0.005	0.79333333
contig_3930	0.004	0.0119	0.63926941
contig_3931	0.0491	0.1869	0.30015236
contig_3933	0.0022	0.0092	0.63541667
contig_3936	0.0018	0.0279	0.31134565
contig_3937	0.0187	0.0787	0.32356257
contig_3938	0.0023	0.0147	0.49797571
contig_3939	0	0.0535	0.15748032
contig_394	0.001	0.0151	0.43824701
contig_3940	0.0033	0.0249	0.38108883
contig_3942	0	0.0073	0.57803468
contig_3944	0.0029	0.0119	0.5890411

contig_3947	0	0.059	0.14492754
contig_3948	0.0008	0.0111	0.51184834
contig_3949	0.0028	0.0385	0.26391753
contig_395	0.001	0.0091	0.57591623
contig_3950	0	0.0179	0.35842294
contig_3955	0.0029	0.0247	0.37175793
contig_396	0.0075	0.0024	1.41129032
contig_3961	0	0.017	0.37037037
contig_3963	0.006	0.0135	0.68085106
contig_3968	0.0059	0.0207	0.51791531
contig_3970	0.0031	0.0197	0.44107744
contig_3971	0.0012	0.0154	0.44094488
contig_3974	0.0071	0.0185	0.6
contig_3977	0.0016	0.0092	0.60416667
contig_3978	0.0009	0.0117	0.50230415
contig_3979	0.0016	0.0258	0.32402235
contig_3981	0.0071	0.0683	0.21839081
contig_3982	0.0027	0.0106	0.61650485
contig_3984	0.0042	0.0529	0.22575517
contig_3986	0.0024	0.0281	0.32545932
contig_3989	0.0054	0.0129	0.67248908
contig_399	0.0008	0.0411	0.21135029
contig_3992	0.0163	0.0516	0.42694805
contig_3993	0.0063	0.0224	0.50308642
contig_3999	0.0004	0.0119	0.47488585
contig_4000	0	0.0269	0.27100271
contig_4002	0.0014	0.0204	0.375
contig_4004	0.0019	0.0102	0.58910891
contig_4007	0	0.0132	0.43103448
contig_4009	0	0.0089	0.52910053
contig_4011	0.0024	0.0122	0.55855856
contig_4013	0.0076	0.0159	0.67953668
contig_4014	0.0066	0.034	0.37727273
contig_4015	0.0021	0.0288	0.31185567
contig_4016	0.002	0.0305	0.2962963
contig_4017	0.0007	0.0082	0.58791209
contig_4018	0.0045	0.0412	0.28320313
contig_4020	0.0045	0.0441	0.26802218
contig_4027	0.0026	0.0094	0.64948454
contig_4028	0.0046	0.0139	0.61087866
contig_4030	0	0.0203	0.330033
contig_4050	0.0041	0.0174	0.51459854
contig_4051	0.0012	0.0412	0.21875
contig_4055	0.0005	0.0117	0.48387097
contig_4057	0	0.0147	0.4048583
contig_406	0.0028	0.0081	0.70718232
contig_407	0.0032	0.0179	0.47311828
contig_4070	0.0012	0.0357	0.24507659
contig_4079	0.0049	0.0043	1.04195804

contig_408	0.0043	0.0123	0.64125561
contig_4081	0.0029	0.0119	0.5890411
contig_4082	0.0043	0.0141	0.593361
contig_4084	0.0026	0.0139	0.52719665
contig_4086	0.0075	0.0166	0.65789474
contig_4087	0.0013	0.009	0.59473684
contig_409	0.0013	0.0108	0.54326923
contig_4090	0.0029	0.0076	0.73295455
contig_4099	0.0048	0.0132	0.63793103
contig_410	0.0075	0.0259	0.48746518
contig_4105	0.0437	0.0722	0.65328467
contig_4106	0.0029	0.0127	0.56828194
contig_4107	0.0016	0.0197	0.39057239
contig_4108	0.0009	0.0137	0.45991561
contig_411	0.0106	0.0469	0.36203866
contig_4119	0	0.0053	0.65359477
contig_4120	0.0066	0.0183	0.58657244
contig_4122	0.0093	0.029	0.4948718
contig_4123	0.0006	0.002	0.88333333
contig_4126	0.0057	0.0373	0.33192389
contig_4129	0.0083	0.0209	0.59223301
contig_4133	0.0007	0.008	0.59444444
contig_4134	0.0081	0.0252	0.51420455
contig_4135	0	0.1125	0.08163265
contig_4136	0.015	0.0742	0.29691211
contig_4138	0.0011	0	1.11
contig_4140	0	0.0704	0.12437811
contig_4141	0.0011	0.0099	0.55778895
contig_4144	0.0011	0.0592	0.16040462
contig_4145	0.0047	0.0242	0.42982456
contig_4147	0.004	0.021	0.4516129
contig_415	0.0115	0.0216	0.68037975
contig_4150	0.003	0.011	0.61904762
contig_4152	0.0037	0.0172	0.50367647
contig_4153	0.0019	0.0156	0.46484375
contig_4154	0	0.0172	0.36764706
contig_4155	0.0015	0.0179	0.41218638
contig_4156	0.0068	0.0103	0.82758621
contig_4157	0.0109	0.0135	0.8893617
contig_4159	0.0616	0.3984	0.17531832
contig_4161	0.009	0.0532	0.30063291
contig_4162	0.0037	0.0351	0.3037694
contig_4167	0.0025	0.0415	0.24271845
contig_4169	0.0006	0.0202	0.35099338
contig_417	0	0.0139	0.41841004
contig_4170	0.0106	0.0941	0.19788665
contig_4171	0.0096	0.0102	0.97029703
contig_418	0.0036	0.0223	0.42105263
contig_419	0	0.0044	0.69444444

contig_420	0.0018	0.0033	0.88721805
contig_424	0.001	0.0191	0.37800687
contig_426	0.001	0.012	0.5
contig_429	0.0028	0.0079	0.7150838
contig_430	0.0021	0.0098	0.61111111
contig_432	0.001	0	1.1
contig_433	0.0016	0.0329	0.27039627
contig_438	0.0067	0.0263	0.4600551
contig_44	0.0048	0.0149	0.59437751
contig_442	0.0009	0	1.09
contig_443	0.0049	0.0883	0.15157681
contig_445	0.0012	0.0136	0.47457627
contig_446	0.0041	0.0084	0.76630435
contig_453	0.0045	0.0223	0.44891641
contig_459	0	0.0365	0.21505376
contig_46	0.0047	0.0342	0.33257919
contig_460	0.0042	0.02	0.47333333
contig_461	0.0031	0.0173	0.47985348
contig_463	0.0006	0.0197	0.35690236
contig_464	0.0015	0.0204	0.37828947
contig_466	0.0022	0.0064	0.74390244
contig_468	0	0.0014	0.87719298
contig_470	0	0.0105	0.48780488
contig_474	0.0048	0.03	0.37
contig_476	0.0083	0.0468	0.3221831
contig_484	0.0055	0.0542	0.24143302
contig_485	0.0022	0.0093	0.63212435
contig_486	0.0032	0.0116	0.61111111
contig_488	0.0037	0.0141	0.56846473
contig_489	0.0057	0.0162	0.59923664
contig_497	0.009	0.0303	0.47146402
contig_50	0.0012	0.0109	0.53588517
contig_500	0.0029	0.0261	0.35734072
contig_502	0.0011	0.0054	0.72077922
contig_506	0.0041	0.0183	0.49823322
contig_507	0.0003	0.0106	0.5
contig_508	0.0126	0.0187	0.78745645
contig_509	0.0059	0.0188	0.55208333
contig_510	0.0054	0.0055	0.99354839
contig_516	0.0054	0.0071	0.9005848
contig_518	0.0025	0.0193	0.42662116
contig_519	0.002	0.0194	0.40816327
contig_52	0.0009	0.0091	0.57068063
contig_521	0.0025	0.0181	0.44483986
contig_523	0.0034	0.0097	0.68020305
contig_529	0.0025	0.0178	0.44964029
contig_532	0.0013	0.0042	0.79577465
contig_534	0.0022	0.0132	0.52586207
contig_536	0.0128	0.0357	0.49890591

contig_539	0.0006	0.0108	0.50961539
contig_546	0.0092	0.0717	0.23500612
contig_548	0	0.0183	0.35335689
contig_549	0.0043	0.0091	0.7486911
contig_550	0.0062	0.0027	1.27559055
contig_558	0	0.0091	0.52356021
contig_560	0.0015	0.0102	0.56930693
contig_565	0.0006	0.0244	0.30813954
contig_566	0	0.0189	0.34602076
contig_57	0.0026	0.0181	0.44839858
contig_571	0.0015	0.0134	0.49145299
contig_573	0.0004	0.0029	0.80620155
contig_574	0.0152	0.0962	0.23728814
contig_575	0.0019	0.0194	0.40476191
contig_576	0.0468	0.3653	0.15134559
contig_578	0.0021	0.0078	0.67977528
contig_580	0.0014	0.0068	0.67857143
contig_583	0.0012	0	1.12
contig_584	0	0.0252	0.28409091
contig_587	0.0003	0.0061	0.63975155
contig_589	0.0013	0.0063	0.69325153
contig_590	0	0.0056	0.64102564
contig_592	0.0043	0.015	0.572
contig_593	0.0009	0.0169	0.40520446
contig_594	0.0115	0.0148	0.86693548
contig_595	0.0036	0.0169	0.50557621
contig_596	0.0102	0.0144	0.82786885
contig_60	0.0039	0.0486	0.23720137
contig_603	0.0019	0.0044	0.82638889
contig_607	0.0043	0.0068	0.85119048
contig_614	0.003	0.0163	0.49429658
contig_619	0.003	0.0062	0.80246914
contig_62	0.0014	0.0114	0.53271028
contig_622	0	0.0065	0.60606061
contig_625	0.0051	0.0411	0.29549902
contig_626	0	0.0525	0.16
contig_628	0	0.0096	0.51020408
contig_63	0.0165	0.0268	0.7201087
contig_631	0	0.014	0.41666667
contig_633	0.0021	0.0022	0.99180328
contig_634	0.0032	0.0064	0.80487805
contig_643	0.0043	0.0249	0.40974212
contig_644	0.0072	0.0098	0.86868687
contig_645	0	0.0041	0.70921986
contig_647	0.0023	0.0213	0.39297125
contig_648	0.0032	0.0161	0.50574713
contig_651	0.001	0.0236	0.32738095
contig_653	0.0094	0.0212	0.62179487
contig_654	0.0007	0.0231	0.32326284

contig_657	0.0013	0.0139	0.47280335
contig_658	0	0.0204	0.32894737
contig_662	0	0.0317	0.23980815
contig_669	0.0055	0.0295	0.39240506
contig_67	0.0012	0.0144	0.45901639
contig_673	0.0035	0.0108	0.64903846
contig_674	0.0022	0.0127	0.53744493
contig_675	0.0036	0.0059	0.85534591
contig_683	0.0073	0.0152	0.68650794
contig_684	0.0017	0.0142	0.48347107
contig_686	0.0022	0.0114	0.57009346
contig_690	0.0043	0.0561	0.21633888
contig_693	0.0073	0.0421	0.33205374
contig_694	0.0024	0.0075	0.70857143
contig_697	0.0029	0.0187	0.44947735
contig_699	0.0095	0.0683	0.24904215
contig_70	0.0105	0.0161	0.78544061
contig_700	0	0.0075	0.57142857
contig_701	0.0004	0.0126	0.46017699
contig_703	0.0045	0.0119	0.66210046
contig_706	0.0011	0.0056	0.71153846
contig_712	0.006	0.0139	0.66945607
contig_713	0.0073	0.0158	0.67054264
contig_716	0.0031	0.0195	0.4440678
contig_718	0.0024	0.0039	0.89208633
contig_719	0.0036	0.0117	0.62672811
contig_72	0.0004	0.0153	0.41106719
contig_720	0.0085	0.0323	0.43735225
contig_721	0.0061	0.0253	0.45609065
contig_722	0.0036	0.0087	0.72727273
contig_723	0.0012	0.0112	0.52830189
contig_724	0.003	0.0262	0.35911602
contig_726	0.0013	0.0417	0.21856867
contig_728	0.0054	0.0558	0.23404255
contig_734	0.0025	0.0077	0.70621469
contig_737	0.0051	0.0177	0.54512635
contig_739	0.0007	0.0144	0.43852459
contig_740	0.0053	0.0108	0.73557692
contig_743	0.0038	0.0085	0.74594595
contig_747	0.0016	0.0093	0.60103627
contig_748	0.0014	0.01	0.57
contig_749	0	0.03	0.25
contig_751	0.003	0.0076	0.73863636
contig_754	0.0055	0.0064	0.94512195
contig_758	0.0031	0	1.31
contig_76	0.0011	0.0282	0.29057592
contig_760	0.0047	0.0098	0.74242424
contig_761	0.0007	0.0145	0.43673469
contig_766	0.0008	0.0749	0.12720848

contig_771	0.0005	0.0253	0.29745043
contig_772	0	0.0141	0.41493776
contig_773	0	0.0141	0.41493776
contig_774	0.0017	0.0175	0.42545455
contig_775	0.0013	0.0462	0.20106762
contig_777	0.0038	0.0199	0.46153846
contig_778	0	0.0066	0.60240964
contig_779	0.0056	0	1.56
contig_781	0.0043	0.1136	0.11569579
contig_786	0.0015	0.0216	0.36392405
contig_787	0	0.0104	0.49019608
contig_789	0.0029	0.0175	0.46909091
contig_791	0.0018	0.0042	0.83098592
contig_794	0	0.0047	0.68027211
contig_797	0.0035	0.0059	0.8490566
contig_799	0	0.0129	0.43668122
contig_800	0.0022	0.0138	0.51260504
contig_801	0	0.0151	0.39840638
contig_803	0.0008	0.014	0.45
contig_806	0.0046	0.0398	0.29317269
contig_807	0	0.0073	0.57803468
contig_810	0.0026	0.0109	0.60287081
contig_811	0.0037	0.0114	0.64018692
contig_816	0.0014	0.0081	0.62983425
contig_821	0.0019	0.0065	0.72121212
contig_824	0.0024	0.0514	0.2019544
contig_831	0.0015	0.0067	0.68862275
contig_834	0.0028	0.0205	0.41967213
contig_837	0.0024	0	1.24
contig_838	0.0055	0.0161	0.59386973
contig_839	0.001	0.0109	0.52631579
contig_84	0.0014	0.0074	0.65517241
contig_840	0.0013	0.062	0.15694444
contig_843	0.0018	0.006	0.7375
contig_845	0.0036	0.0125	0.60444444
contig_849	0.004	0.0342	0.31674208
contig_85	0.0018	0.0056	0.75641026
contig_857	0.0016	0.0126	0.51327434
contig_859	0.0023	0.0069	0.72781065
contig_863	0.0032	0.0069	0.78106509
contig_864	0.0027	0	1.27
contig_867	0.0044	0.0106	0.69902913
contig_87	0.002	0.0047	0.81632653
contig_870	0.0013	0.0117	0.52073733
contig_872	0.0009	0.0133	0.46781116
contig_874	0.0008	0.0053	0.70588235
contig_879	0.0013	0.0312	0.27427185
contig_88	0	0.017	0.37037037
contig_880	0.003	0.0053	0.8496732

contig_882	0.0023	0.0181	0.43772242
contig_886	0.0022	0.0134	0.52136752
contig_887	0.0006	0.0168	0.39552239
contig_890	0	0.0062	0.61728395
contig_892	0.0008	0.0172	0.39705882
contig_896	0.0018	0.0069	0.69822485
contig_902	0.0032	0.0142	0.54545455
contig_905	0.0035	0.0061	0.83850932
contig_910	0.0009	0.0108	0.52403846
contig_913	0.0015	0.0341	0.26077098
contig_914	0.0009	0.0127	0.48017621
contig_915	0.0056	0.0238	0.46153846
contig_918	0.0008	0.0106	0.52427185
contig_919	0	0.0309	0.24449878
contig_925	0	0.0115	0.46511628
contig_928	0.0099	0.0287	0.51421189
contig_929	0	0.0142	0.41322314
contig_930	0.001	0.0119	0.50228311
contig_931	0.0018	0.0161	0.45210728
contig_935	0.0005	0.0126	0.46460177
contig_936	0.003	0.0409	0.25540275
contig_937	0.0009	0.0102	0.53960396
contig_938	0.0005	0.0087	0.56149733
contig_939	0.0014	0.0131	0.49350649
contig_941	0.0048	0.0193	0.50511945
contig_944	0.002	0.0358	0.26200873
contig_945	0.0011	0.0365	0.23870968
contig_95	0	0.0035	0.74074074
contig_950	0.0064	0.0525	0.2624
contig_953	0.0008	0.0132	0.46551724
contig_957	0.0027	0.0072	0.73837209
contig_960	0.0025	0.0217	0.39432177
contig_962	0.0024	0.0089	0.65608466
contig_965	0.0014	0.0113	0.53521127
contig_968	0.0024	0.0178	0.44604317
contig_97	0	0.0028	0.78125
contig_970	0.0314	0.053	0.65714286
contig_979	0	0.0182	0.35460993
contig_980	0.005	0.0282	0.39267016
contig_981	0.008	0.0263	0.49586777
contig_982	0.0025	0.014	0.52083333
contig_983	0.0025	0.0073	0.72254335
contig_984	0	0.0038	0.72463768
contig_985	0.0037	0.011	0.65238095
contig_986	0.001	0.0058	0.69620253
contig_989	0.0069	0.0423	0.32313576
contig_990	0.0007	0.0045	0.73793103
contig_992	0.0009	0.0166	0.40977444
contig_994	0.0025	0.0154	0.49212598

contig_996	0.0039	0.0164	0.52651515
contig_998	0.0006	0.04	0.212
