# Supplementary material for: Evolution of Transcriptomes in Early-Generation Hybrids of the Apomictic Ranunculus auricomus Complex (Ranunculaceae)
Source: Int J Mol Sci. 2022 Nov 10;23(22):13881. doi: 10.3390/ijms232213881 (PMC9697309; doi:10.3390/ijms232213881)
Supplement: Supplementary file 1 [file ijms-23-13881-s001.zip › Supplemental Table S5_dNdS_revised.pdf]

Supplementary Table S5. Pairwise dN/dS ratios of 79 annotated genes with ratios >1.0 in the average of all six pairwise comparisons, after filtering out parental, intraspecific and hybrid-hybrid polymorphisms (see Table 2). * = average hybrid-parent <1.0, but found in GO terms Pellino et al. 2013 (see discussion); red = same GO terms as in Pellino et al. 2013.

		**parent-hybrid comparisons**						**Average**
	note	**10137-3 vs. F10**	**10137-3 vs. C4**	**10137-8 vs. C4**	**10137-8 vs. F10**	**P15-02 vs. C4**	**P15-02 vs. F10**	
contig_52	1	3.70	0.10	0.10	3.70	0.04	0.19	1.30
contig_118	1	9.00	1.30	0.12	0.59	0.22	0.22	1.91
contig_121	1	0.29	8.30	4.70	0.29	0.27	0.42	2.38
contig_136	1	0.39	1.00	3.80	0.67	0.10	0.20	1.03
contig_145	1	0.19	0.41	0.25	0.22	5.20	0.36	1.10
contig_172	1	5.90	3.50	0.33	0.55	0.17	0.29	1.79
contig_180	1	3.00	0.05	0.05	3.00	0.25	1.18	1.25
contig_187	1	20.70	0.60	0.31	1.17	0.27	0.26	3.88
contig_213	1	4.40	0.05	0.41	4.40	0.14	0.21	1.60
contig_247	1	0.20	5.60	0.65	0.38	0.49	0.66	1.33
contig_432	1	1.00	3.90	3.90	1.00	3.90	1.00	2.45
contig_446	1	0.65	2.60	0.17	0.16	0.25	8.80	2.10
contig_466	1	0.34	0.64	3.20	0.38	0.38	3.20	1.36
contig_532	1	3.60	0.24	0.44	3.60	0.24	0.69	1.47
contig_576	1	0.90	0.32	0.19	9.90	0.19	9.90	3.56
contig_583	1	4.70	1.00	1.00	4.70	1.00	4.70	2.85
contig_669	1	0.19	3.30	3.30	0.11	3.30	0.11	1.72
contig_675	1	3.70	3.70	1.00	0.65	0.38	0.38	1.63
contig_683	1	0.43	4.00	0.37	0.32	1.22	0.49	1.14
contig_703	1	3.90	1.00	0.60	0.86	0.31	0.45	1.19
contig_713	1	0.10	0.10	12.60	0.78	12.60	0.78	4.49
contig_748	1	0.20	3.60	3.60	0.20	0.39	0.11	1.35
contig_751	1	0.65	0.20	0.38	5.30	0.12	0.34	1.17
contig_831	1	1.00	3.80	3.80	1.00	0.09	0.35	1.67

contig_845	1	0.13	0.09	0.09	1.00	7.30	0.19	1.47
contig_867	1	0.40	0.12	0.67	1.13	0.51	4.70	1.26
contig_880	1	0.11	8.60	8.60	0.39	6.10	0.06	3.98
contig_928	1	0.50	0.14	0.93	4.80	0.65	0.41	1.24
contig_1036	1	0.50	0.68	1.00	0.42	0.69	2.80	1.01
contig_1064	1	3.50	3.50	0.47	0.47	0.26	0.26	1.41
contig_1106	1	0.30	0.17	0.58	0.29	7.30	4.20	2.14
contig_1127	1	0.63	0.24	0.38	8.00	0.24	8.00	2.92
contig_1263	1	0.19	1.00	1.00	0.19	0.33	4.50	1.20
contig_1275	1	1.00	1.00	1.00	1.00	1.00	1.00	1.00
contig_1282	1	0.13	4.80	0.11	0.15	0.18	2.90	1.38
contig_1333	1	4.00	5.50	0.08	0.11	0.08	0.11	1.65
contig_1423	1	0.05	0.16	6.40	0.84	0.60	1.37	1.57
contig_1451	1	0.37	0.12	0.02	8.30	0.09	0.25	1.53
contig_1506	1	0.61	6.40	0.37	1.00	8.20	0.37	2.83
contig_1591	1	0.92	0.38	0.20	0.24	0.44	4.60	1.13
contig_1676	1	0.73	0.40	5.00	1.79	0.64	0.60	1.53
contig_1701	1	0.43	1.00	0.71	6.70	0.52	8.60	2.99
contig_1763	1	9.30	0.10	0.51	0.78	0.82	1.18	2.12
contig_1803	1	0.23	0.39	0.72	5.20	0.54	0.39	1.24
contig_1905	*	0.06	0.12	0.25	0.08	0.19	0.09	0.13
contig_2172	1	0.14	8.40	0.51	0.45	0.15	0.14	1.63
contig_2299	1	0.14	2.70	0.14	0.11	0.86	4.40	1.39
contig_2337	1	3.60	0.68	0.68	0.43	0.68	0.43	1.08
contig_2413	1	0.26	0.36	0.38	0.39	5.20	18.20	4.13
contig_2439	1	0.08	0.11	0.11	0.08	4.90	0.98	1.04
contig_2510	1	0.93	6.50	0.26	0.46	0.80	1.90	1.81
contig_2558	1	0.61	0.62	3.40	0.50	0.63	0.43	1.03
contig_2785	1	0.05	2.50	2.50	1.00	2.50	0.05	1.43

contig_2835	1	0.38	0.05	0.94	0.13	0.45	5.70	1.28
contig_2874	1	0.12	3.20	3.20	0.12	0.12	0.10	1.14
contig_2896	1	3.20	2.10	0.17	0.11	0.36	0.65	1.10
contig_2940	1	0.36	0.21	0.39	0.68	0.68	5.70	1.34
contig_3193	1	0.34	0.34	5.60	0.46	0.63	0.15	1.25
contig_3277	1	0.35	0.40	4.90	0.93	0.50	1.00	1.35
contig_3282	1	0.23	0.29	7.70	0.61	0.34	0.08	1.54
contig_3431	1	0.17	0.33	0.17	0.17	8.40	0.20	1.57
contig_3535	1	0.39	5.10	0.55	0.39	0.28	0.24	1.16
contig_3562	1	4.50	0.08	1.00	1.00	0.08	0.08	1.12
contig_3609	1	0.43	0.43	2.70	2.70	0.23	0.23	1.12
contig_3685	1	1.01	0.73	1.00	1.00	3.20	1.01	1.33
contig_3710	1	0.08	0.83	7.00	0.17	0.28	1.00	1.56
contig_3717	1	0.90	3.10	0.38	3.10	0.12	0.34	1.32
contig_3729	1	3.40	0.11	0.11	3.40	0.11	3.40	1.75
contig_3741	*	0.22	0.35	0.30	0.81	0.56	0.13	0.40
contig_3746	1	0.31	0.13	0.05	9.50	0.29	0.93	1.87
contig_3769	1	0.33	1.00	0.09	3.60	0.09	3.60	1.45
contig_3783	1	5.20	0.03	1.00	0.16	0.32	0.02	1.12
contig_3816	1	0.06	0.04	3.50	3.50	0.67	0.67	1.41
contig_3826	1	4.60	2.80	0.14	0.40	0.66	0.91	1.59
contig_3933	1	0.22	0.15	0.67	0.22	0.67	4.60	1.09
contig_3938	*	0.31	0.55	0.34	0.23	0.22	0.33	0.33
contig_3970	1	0.09	0.35	5.90	0.22	0.09	1.00	1.28
contig_3989	1	1.13	6.90	1.13	0.41	0.60	0.50	1.78
contig_4086	1	0.36	1.00	0.33	0.92	0.36	7.60	1.76
contig_4129	1	0.62	0.20	0.62	10.70	0.32	0.53	2.17
contig_4136	1	2.50	2.50	0.29	0.29	1.00	0.17	1.12
contig_4171	1	0.70	2.45	1.86	0.25	1.57	0.68	1.25
