# Supplementary material for: Evolution of Transcriptomes in Early-Generation Hybrids of the Apomictic Ranunculus auricomus Complex (Ranunculaceae)
Source: Int J Mol Sci. 2022 Nov 10;23(22):13881. doi: 10.3390/ijms232213881 (PMC9697309; doi:10.3390/ijms232213881)

Supplemental Figure S1. Venn diagrams showing the number of contigs from dN/dS analysis that showed sequence variability within hybrid (olive) and parental (red) taxa, respectively (A) and genes, that did not show variability (B). In addition, the variability is shown within the parental taxa, comparing all three individuals (cyan) and the two *R. notabilis* (pink) samples (C, D).

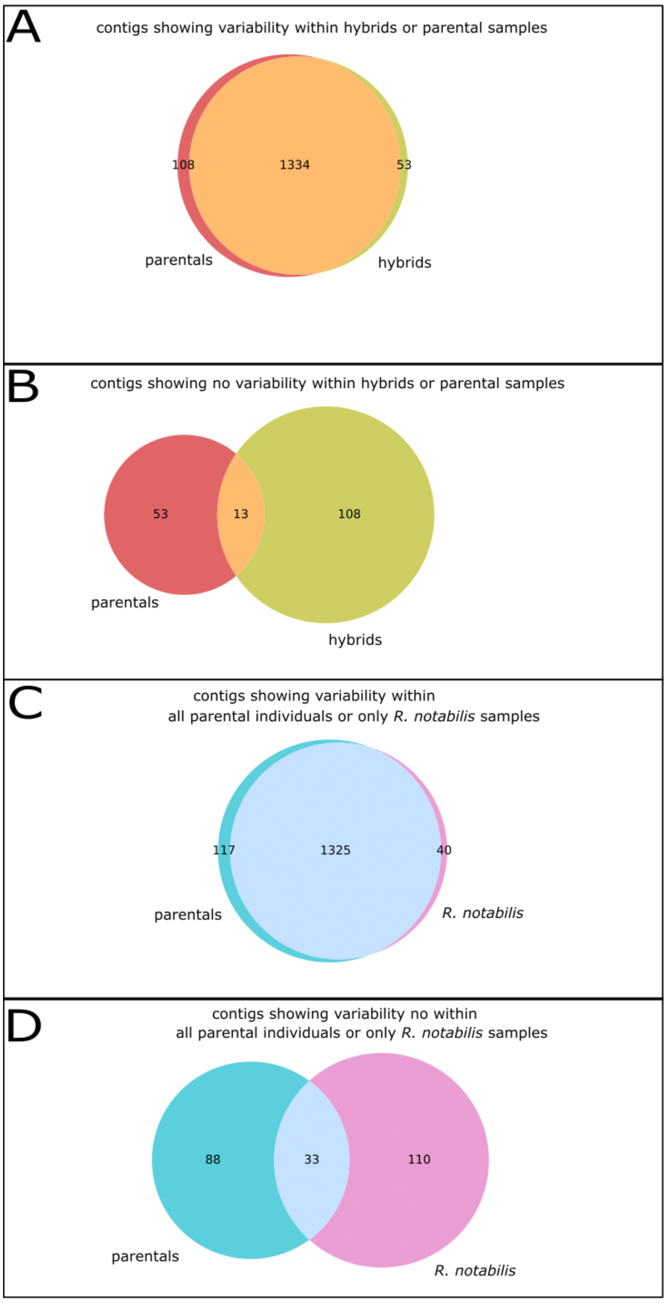

Supplemental Figure S2. (A–F) Hybridization Networks. The si minimum hybridization networks reconstructed from the 1,514 alignments comprising at least 30 ungapped amino acids across all samples used for calculation of dN/dS ratios.

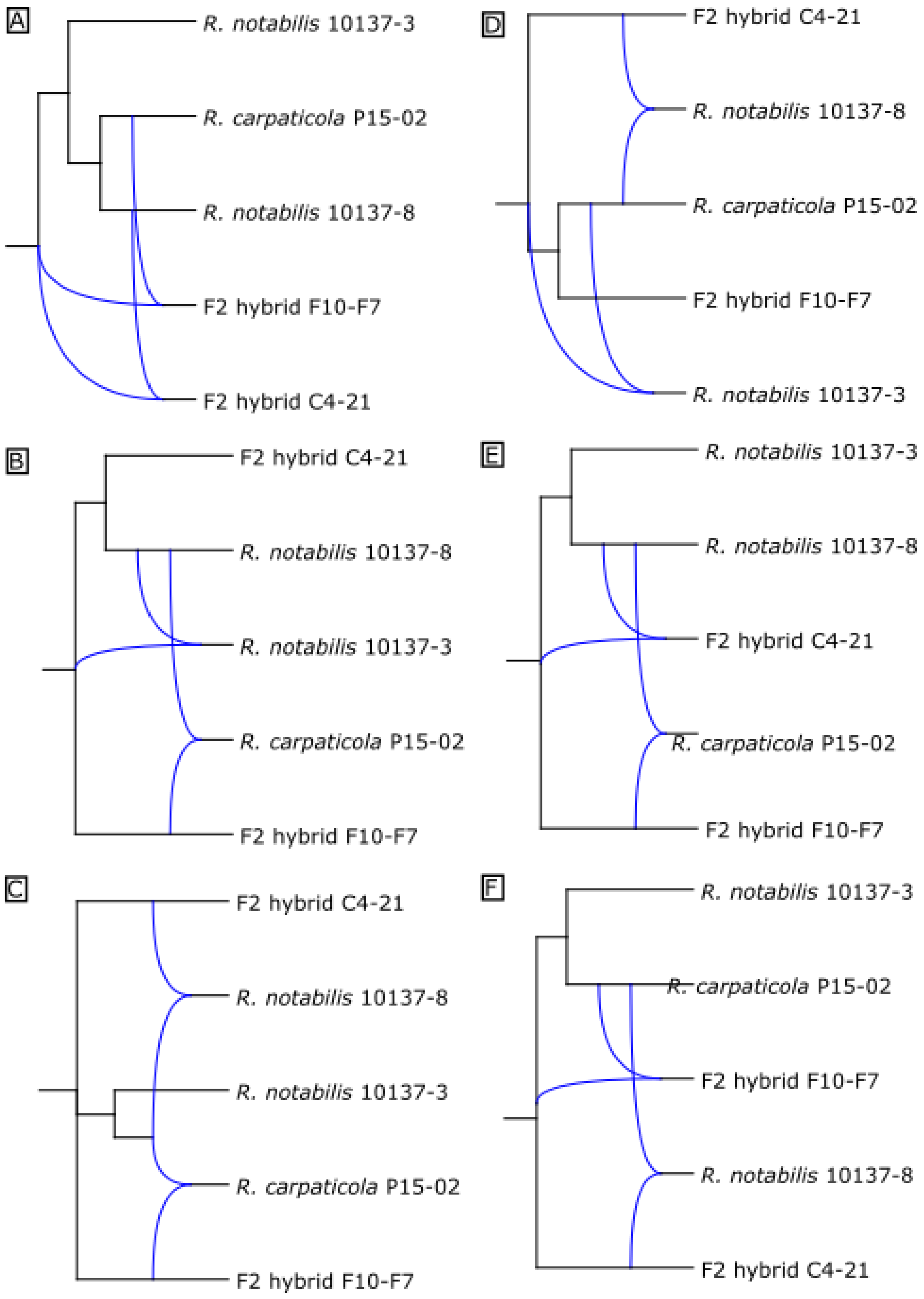

Supplementary Figure S3. Boxplot showing pairwise dN/dS ratios across the longest ungapped section of the putative nuclear single copy loci as resolved by the Nej-Gojobori algorithm as implemented in Bioperl. Parent-hyrid comparisons marked in bold.

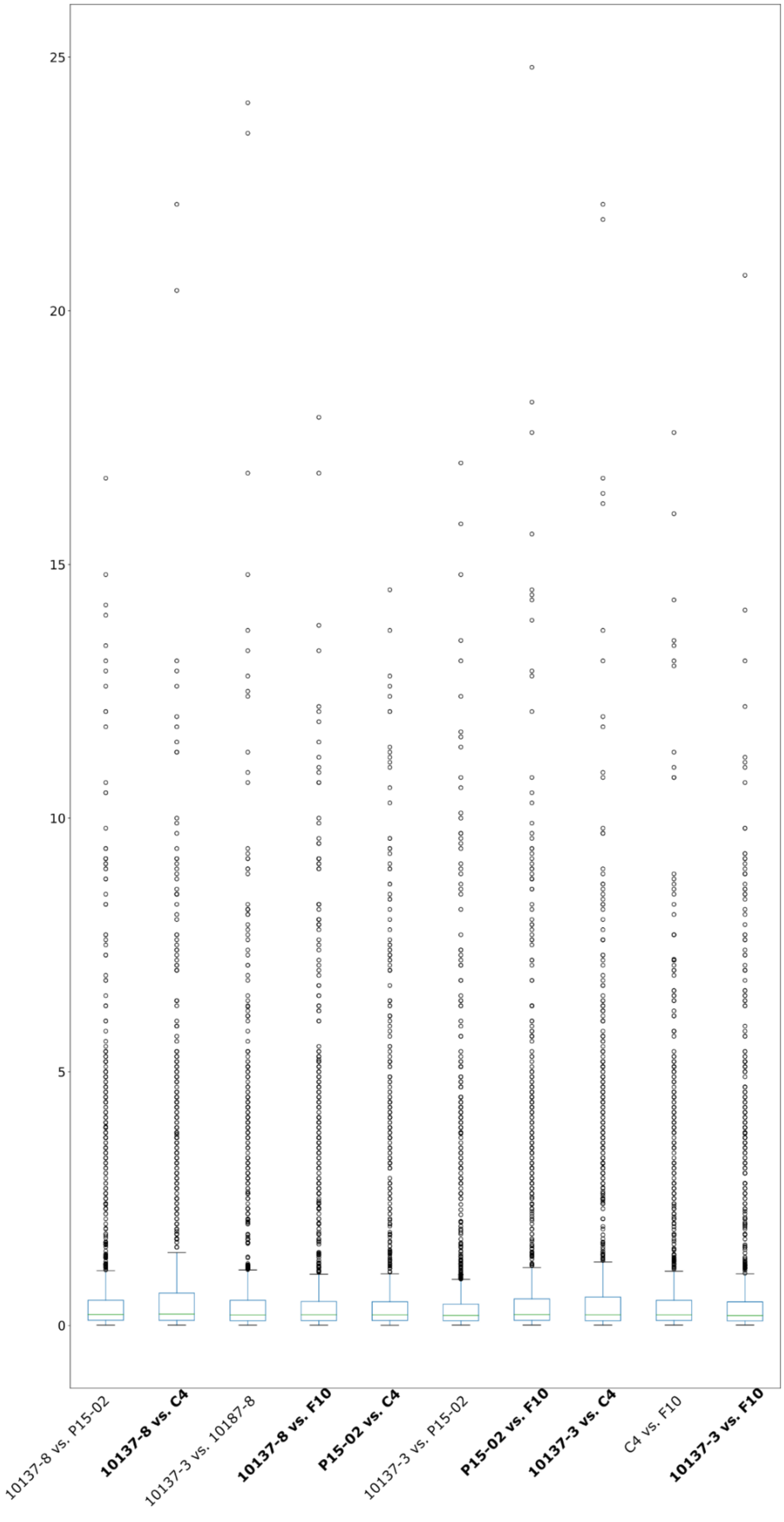

Supplementary Figure S4. Biological Process Top level Gene Ontology for all putative nuclear single copy loci (top), of all putative nuclear single copy loci linked to reproduction (middle) and in the putative single copy loci under diversifying selection in the F2 hybrids only, which are involved in the reproductive process.

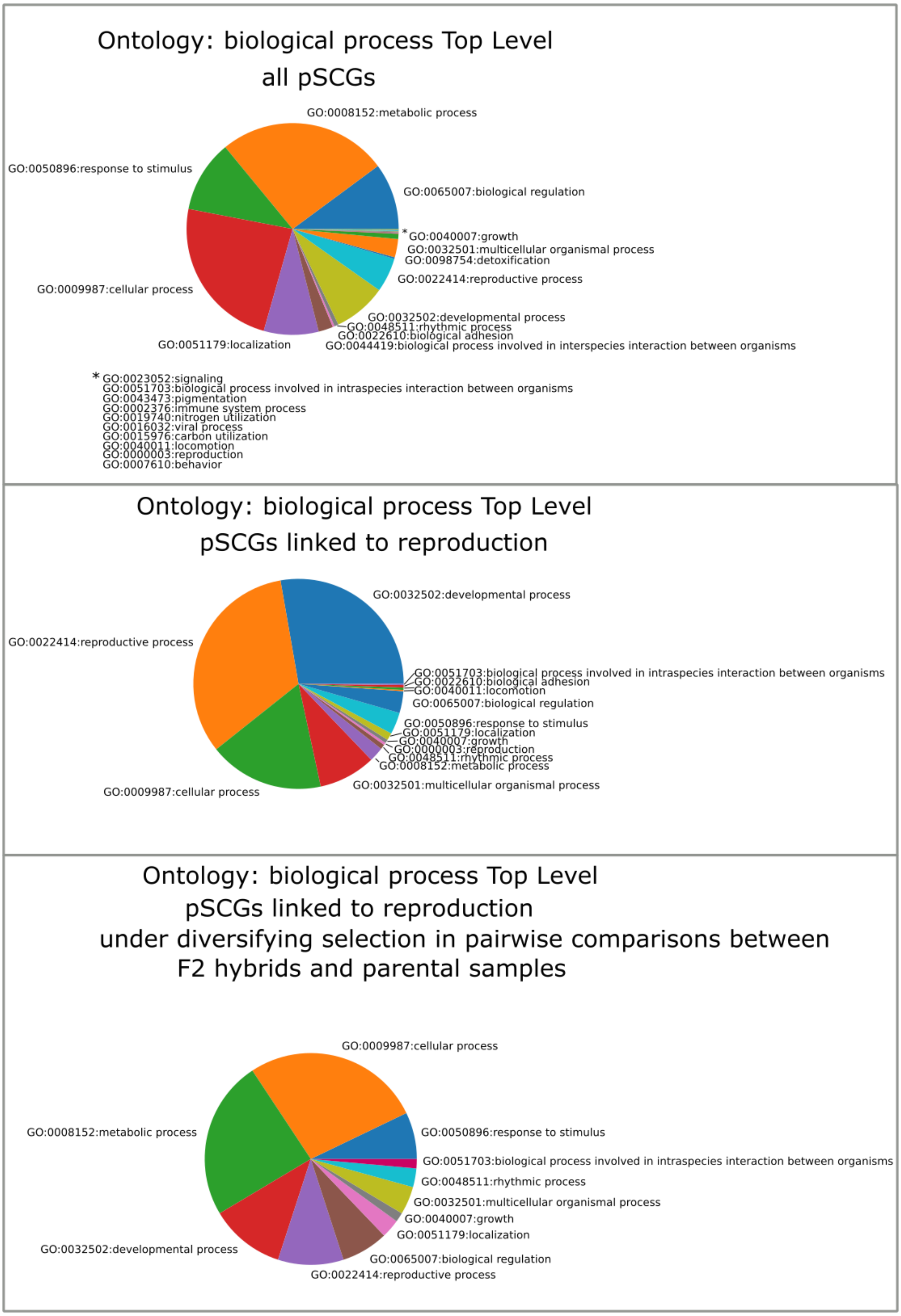

Supplemental Figure S5. Functional involvement in the reproductive process of all putative single copy genes. "Onset" means genes ivolved in onset of the reproductive process.

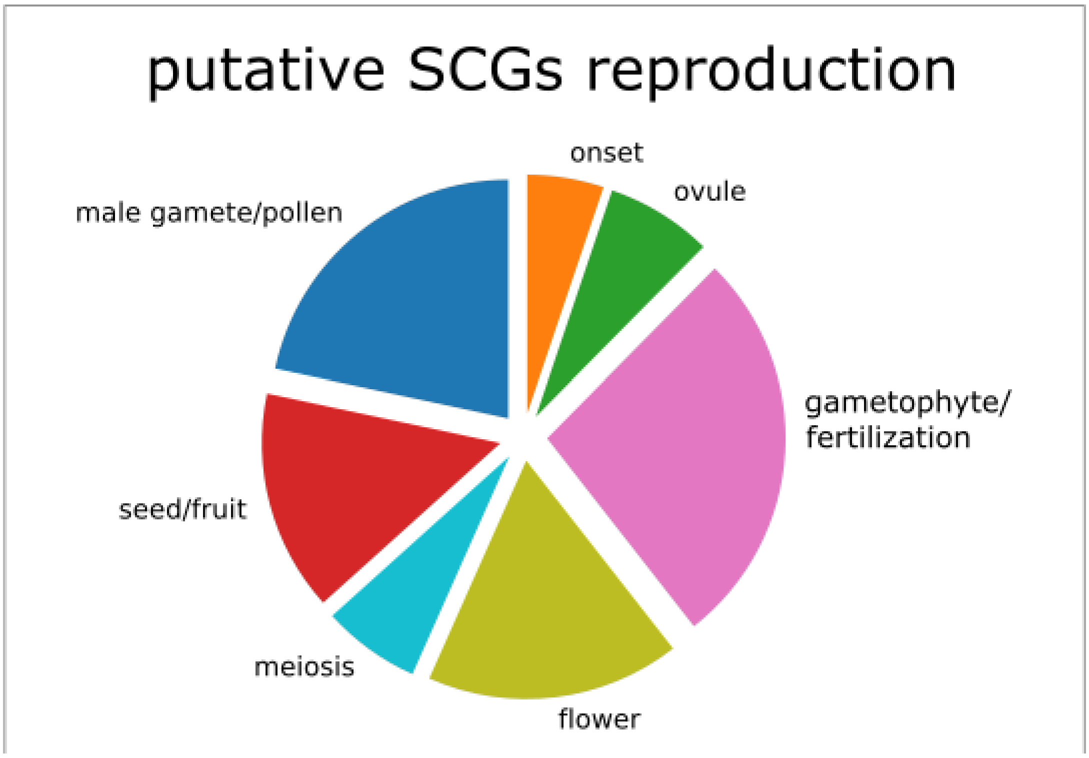

Supplement: Supplementary file 1 [file ijms-23-13881-s001.zip › Supplementary Figures S1-S5.pdf]
